# MUC1-C dependence in treatment-resistant prostate cancer uncovers a target for antibody-drug conjugate therapy

**DOI:** 10.1172/jci.insight.190924

**Published:** 2025-06-24

**Authors:** Keisuke Shigeta, Tatsuaki Daimon, Hiroshi Hongo, Sheng-Yu Ku, Hiroki Ozawa, Naoki Haratake, Atsushi Fushimi, Ayako Nakashoji, Atrayee Bhattacharya, Shinkichi Takamori, Michihisa Kono, Masahiro Rokugo, Yuto Baba, Takeo Kosaka, Mototsugu Oya, Justine Jacobi, Mark D. Long, Himisha Beltran, Donald Kufe

**Affiliations:** 1 Dana-Farber Cancer Institute, Harvard Medical School, Boston, Massachusetts, USA.; 2 Department of Urology, Keio University School of Medicine, Tokyo, Japan.; 3 Department of Biostatistics & Bioinformatics, Roswell Park Comprehensive Cancer Center, Buffalo, New York, USA.

**Keywords:** Oncology, Therapeutics, Cancer, Drug therapy, Urology

## Abstract

Androgen receptor–positive prostate cancer (PC), castration-resistant prostate cancer (CRPC), and neuroendocrine prostate cancer (NEPC) invariably become resistant to treatment with targeted and cytotoxic agents. Multiple pathways have been identified as being responsible for these pleiotropic mechanisms of resistance. The mucin 1 (*MUC1*) gene is aberrantly expressed in CRPC/NEPC in association with poor clinical outcomes; however, it is not known if the oncogenic MUC1-C/M1C protein drives treatment resistance. We demonstrated that MUC1-C is necessary for resistance of (i) PC cells to enzalutamide (ENZ) and (ii) CRPC and NEPC cells to docetaxel (DTX). Our results showed that MUC1-C–mediated resistance is conferred by upregulation of aerobic glycolysis and suppression of reactive oxygen species necessary for self-renewal. Dependence of these resistant phenotypes on MUC1-C for the cancer stem cell (CSC) state identified a potential target for treatment. In this regard, we further demonstrated that targeting MUC1-C with an M1C antibody-drug conjugate (ADC) is highly effective in suppressing (i) self-renewal of drug-resistant CRPC/NEPC CSCs and (ii) growth of treatment-emergent NEPC tumor xenografts derived from drug-resistant cells and a patient with refractory disease. These findings uncovered a common MUC1-C–dependent pathway in treatment-resistant CRPC/NEPC progression and identified MUC1-C as a target for their therapy with an M1C ADC.

## Abstract 2

Our findings revealed MUC1-C as a new therapeutical target particluarly for drug-resistant CRPC/NEPC treatment with an MUC1-C targeting ADC.

## Graphical abstract

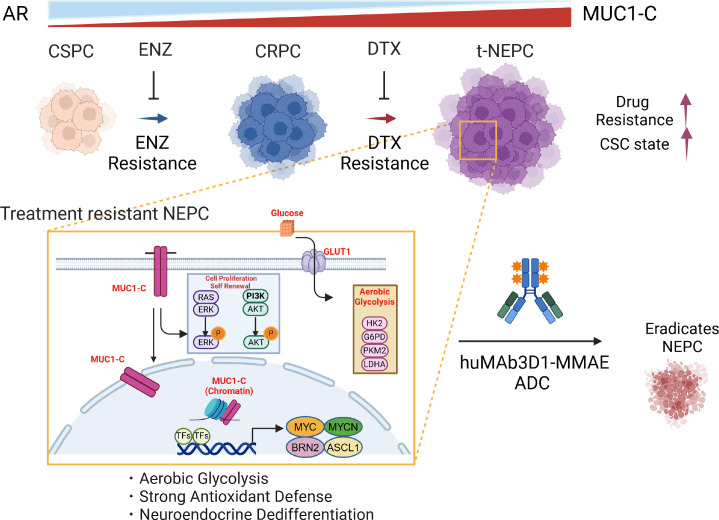

## Introduction

Prostate cancer (PC) arises as an androgen receptor–driven (AR-driven) disease (1). Hormonal therapies targeting the AR signaling pathway are a mainstay of therapy for patients with advanced disease; however, acquired resistance to these agents is an inevitable outcome (2, 3). Approved therapies for castration-resistant PC (CRPC) include agents that target AR signaling (e.g., enzalutamide, abiraterone acetate), chemotherapy (e.g., docetaxel, cabazitaxel), radionuclide therapy (e.g., radium-223, LuPSMA-617), and genomic driven therapies, such as poly(ADP-ribose) polymerase inhibitors for breast cancer gene–mutated PC (2–4). Patients demonstrate variable responses to these therapies, and, increasingly, aggressive variants emerge, including those that are AR negative with neuroendocrine features (5–8). Pathologically, both de novo and treatment-emergent neuroendocrine prostate cancer (t-NEPC) share similar characteristics with small cell lung cancer and other types of small cell carcinomas (9, 10). Patients who progress to t-NEPC have a median overall survival of less than 1 year (7, 8, 11). Despite significant advances in PC management over the last decade, CRPC and t-NEPC are often lethal, and PC remains a leading cause of cancer death worldwide. Of note is that there is no recognized common pathway in the progression of treatment-resistant AR-positive PC, CRPC, and t-NEPC.

The mucin 1 (*MUC1*) gene is aberrantly expressed in advanced CRPC and NEPC (12). *MUC1* expression is significantly increased in CRPC tumors compared with localized, hormone-naive PCs as evidenced by median log_2_ values (12). *MUC1* is amplified in 30% of CRPCs with NEPC characteristics as compared with 6% in the Stand Up 2 Cancer CRPC and 2% in The Cancer Genome Atlas primary PC cohorts (12). Upregulation of MUC1 in advanced CRPC is associated with aggressive disease (13–18). *MUC1* encodes an oncogenic C-terminal transmembrane subunit (MUC1-C) (19–21). In androgen-dependent LNCaP PC cells, MUC1-C suppresses AR signaling and induces the neural BRN2 transcription factor in association with induction of MYCN, EZH2, and NE differentiation markers (ASCL1, AURKA, and SYP) linked to NEPC progression (12). Furthermore, MUC1-C activates the BAF (SWI/SNF) and poly-bromo BAF chromatin-remodeling complexes in CRPC/NEPC cells (22, 23). In this way, MUC1-C induces changes in chromatin accessibility and gene expression in association with driving the PC cancer stem cell (CSC) state (24). Of potential translational importance, targeting MUC1-C suppresses PC self-renewal capacity and tumorigenicity (12).

There is no known common effector of PC treatment resistance. The present studies demonstrate that LNCaP cells selected for resistance to the AR antagonist enzalutamide (ENZ) are dependent on MUC1-C for the drug-resistant phenotype. Our results further demonstrate that AR-negative DU-145 CRPC and H660 NEPC cells are dependent on MUC1-C for resistance to docetaxel (DTX). We report that MUC1-C confers treatment resistance in CRPC and NEPC progression by a mechanism involving regulation of aerobic glycolysis and redox balance necessary for maintaining the CSC state. Consistent with this MUC1-C dependence, we demonstrate that an anti–MUC1-C (M1C) antibody-drug conjugate (ADC) is highly effective for the treatment of drug-resistant CRPC and NEPC. The potential clinical relevance resides in the findings that MUC1-C is a common effector of treatment resistance in PC progression and is a potential target in this advanced disease setting.

## Results

### AR-positive LNCaP cells are dependent on MUC1-C for acquired ENZ resistance.

**Figure 1:**
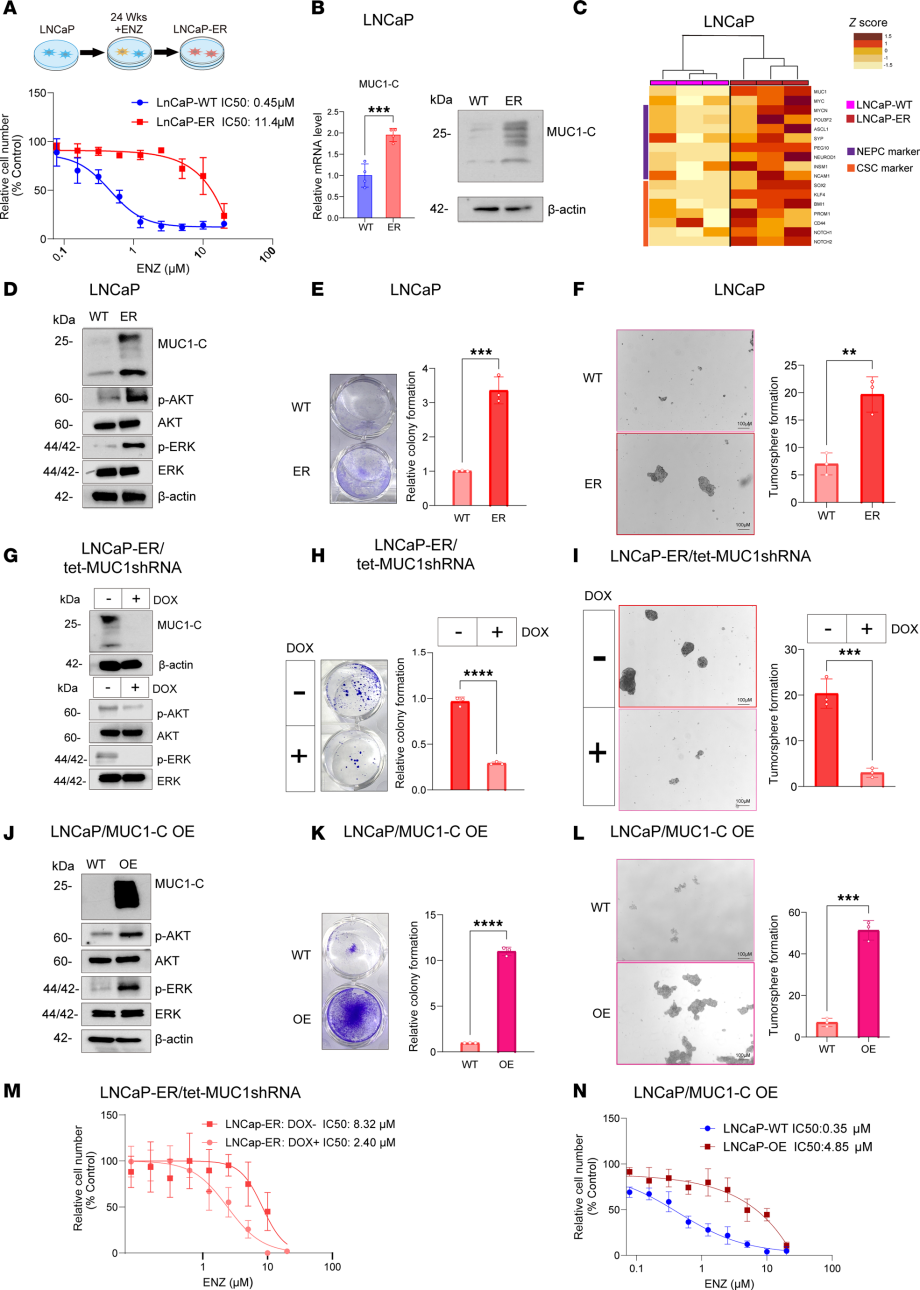
LNCaP-ER cells are dependent on MUC1-C for ENZ resistance and self-renewal. (**A**) Parental LNCaP and LNCaP-ER cells treated with ENZ for 3 days were analyzed for cell viability by Alamar blue staining. (**B**) LNCaP and LNCaP-ER cells were analyzed for MUC1-C transcripts by quantitative reverse-transcription PCR (qRT-PCR) (left). Immunoblot analysis of lysates from LNCaP and LNCaP-ER cells (right). (**C**) Heatmap of NE and CSC marker gene expression from qRT-PCR analysis of biological triplicates of LNCaP-WT and LNCaP-ER cells. (**D**) Immunoblot analysis of lysates from LNCaP and LNCaP-ER cells run contemporaneously in parallel. (**E**) LNCaP and LNCaP-ER cells treated with 10 μM ENZ for 3 days were analyzed for colony formation. Representative photomicrographs of stained colonies (left). Results (mean ± SD of 3 determinations) expressed as relative colony number compared with untreated cells (assigned a value of 1) (*t* test; *n* = 3) (right). (**F**) LNCaP and LNCaP-ER cells were analyzed for tumorsphere formation. Representative photomicrographs of tumorspheres (left). Results (mean ± SD of 3 determinations) expressed as tumorsphere number (*t* test; *n* = 3) (right). (**G**) Immunoblot analysis of lysates from LNCaP-ER/tet-MUC1shRNA cells treated with vehicle or doxycycline (DOX) for 7 days run at different times. (**H**) LNCaP-ER/tet-MUC1shRNA cells treated with vehicle or DOX for 7 days were analyzed for colony formation (*t* test; *n* = 3). (**I**) LNCaP-ER/tet-MUC1shRNA cells treated with vehicle or DOX for 7 days were analyzed for tumorsphere formation (*t* test; *n* = 3). (**J**) Immunoblot analysis of lysates from LNCaP and LNCaP/MUC1-C OE cells run contemporaneously in parallel. (**K**) LNCaP and LNCaP/MUC1-C OE cells were analyzed for colony formation (*t* test; *n* = 3). (**L**) LNCaP and LNCaP/MUC1-C OE cells were analyzed for tumorsphere formation (*t* test; *n* = 3). (**M**) LNCaP-ER/tet-MUC1shRNA cells treated with vehicle or DOX for 7 days and then with ENZ for 3 days were analyzed for cell viability. (**N**) LNCaP and LNCaP/MUC1-C OE cells were treated with ENZ for 3 days and analyzed for cell viability. **P < 0.01, ***P < 0.001, and ****P < 0.0001.

Selection of AR-positive LNCaP PC cells for androgen-independent growth is associated with upregulation of MUC1-C and suppression of the AR signaling axis (12). Here, selection of LNCaP cells for resistance to ENZ (Figure 1A) demonstrated that ENZ-resistant LNCaP-ER cells had increased levels of MUC1-C transcripts and protein (Figure 1B). Analysis of LNCaP-ER versus LNCaP cells demonstrated that MUC1-C associated with upregulation of genes encoding markers of NE dedifferentiation and the CSC state (Figure 1C). The MUC1-C cytoplasmic domain functions as a scaffold for activating the PI3K/AKT and MEK/ERK pathways (25, 26). Analysis of LNCaP-ER versus LNCaP cells further demonstrated increases in phosphorylated (p-) AKT and p-ERK levels (Figure 1D), clonogenic survival (Figure 1E), and self-renewal capacity as defined by the ability of a CSC to replicate itself and as evidenced by tumorsphere formation (Figure 1F). Treatment of LNCaP-ER/tet-MUC1shRNA cells with DOX to upon induction silence MUC1-C resulted in suppression of (i) p-AKT and p-ERK (Figure 1G), (ii) colony formation (Figure 1H), and (iii) self-renewal capacity (Figure 1I). In extending these results, treatment of LNCaP-ER cells with the GO-203 inhibitor, which targets the MUC1-C at a CQC motif in the cytoplasmic domain necessary for dimerization, suppressed colony formation (Supplemental Figure 1A; supplemental material available online with this article; https://doi.org/10.1172/jci.insight.190924DS1) and self-renewal capacity (Supplemental Figure 1B). RNA-Seq further demonstrated that silencing MUC1-C in LNCaP-ER cells was associated with downregulation of the HALLMARK G2M CHECKPOINT and HALLMARK E2F TARGETS gene signatures (Supplemental Figure 1, C–E). For a gain-of-function model, LNCaP/MUC1-C OE cells overexpressing exogenous MUC1-C were analyzed by RNA-Seq, which demonstrated that MUC1-C was associated with upregulation of the HALLMARK G2M CHECKPOINT and HALLMARK E2F TARGETS gene signatures (Supplemental Figure 1, F–H). LNCaP/MUC1-C OE cells exhibited upregulation of p-AKT and p-ERK (Figure 1J), colony formation (Figure 1K), and self-renewal capacity (Figure 1L). Noteworthy is that silencing MUC1-C in LNCaP-ER cells with different MUC1shRNAs suppressed ENZ resistance (Figure 1M and Supplemental Figure 1I), and upregulation of MUC1-C in LNCaP/MUC1-C OE cells conferred ENZ resistance (Figure 1N). These findings indicate that MUC1-C is necessary for activation of a proliferative ENZ-resistant phenotype that is associated with self-renewal and the CSC state.

### MUC1-C/MYC–driven aerobic glycolysis is necessary for the LNCaP-ER resistant phenotype and CSC state.

**Figure 2:**
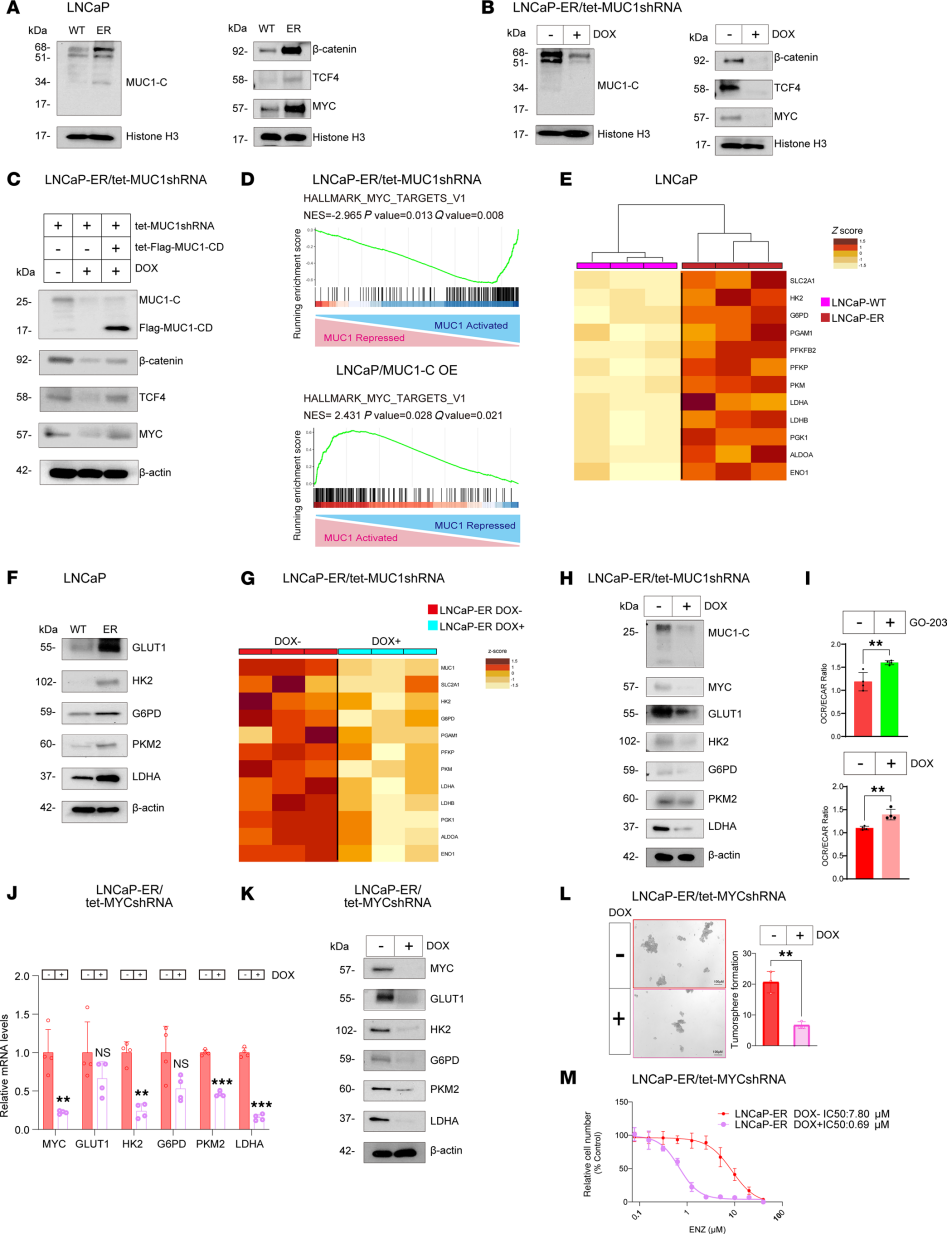
MUC1-C/MYC pathway regulates aerobic glycolysis, ENZ resistance, and the CSC state in LNCaP-ER cells. (**A** and **B**) Immunoblot analysis of chromatin from LNCaP and LNCaP-ER cells (**A**) and LNCaP-ER/tet-MUC1shRNA (**B**) cells treated with DOX for 7 days each run contemporaneously in parallel. (**C**) Immunoblot analysis of lysates from LNCaP-ER cells expressing the indicated vectors treated with DOX for 7 days run contemporaneously in parallel. (**D**) GSEA of RNA-Seq data from LNCaP-ER cells with MUC1-C silencing and LNCaP/MUC1-C OE cells using the HALLMARK MYC TARGETS V1 signature. NES, normalized enrichment score. (**E**) Heatmap of glycolysis gene expression in LNCaP and LNCaP-ER cells. (**F**) Immunoblot analysis of lysates from LNCaP and LNCaP-ER cells run contemporaneously in parallel. (**G**) Heatmap of glycolysis gene expression of LNCaP-ER/tet-MUC1shRNA cells treated with DOX for 7 days. (**H**) Immunoblot analysis of lysates from LNCaP-ER/tet-MUC1shRNA cells treated with DOX for 7 days run contemporaneously in parallel. (**I**) LNCaP-ER cells treated with 3 μM GO-203 (upper) and LNCaP-ER/tet-MUC1shRNA cells treated with DOX for 7 days (lower) were assayed for OCR and ECAR. The OCR/ECAR results (mean ± SD of 4 determinations) are expressed as the relative ratio compared with untreated cells (*t* test; *n* = 3). (**J**) LNCaP-ER/tet-MYCshRNA cells treated with DOX for 7 days were analyzed for the indicated transcripts by qRT-PCR. The results (mean ± SD of 4 determinations) are expressed as relative levels compared with untreated cells (assigned a value of 1) (*t* test; *n* = 3). (**K**) Immunoblot analysis of lysates from LNCaP-ER/tet-MYCshRNA cells treated with DOX for 7 days run contemporaneously in parallel. (**L**) LNCaP-ER/tet-MYCshRNA cells treated with vehicle or DOX for 7 days were analyzed for tumorsphere formation. Representative photomicrographs of tumorspheres (left). Results (mean ± SD of 3 determinations) expressed as tumorsphere number (*t* test; *n* = 3). (**M**) LNCaP-ER/tet-MYCshRNA cells treated with DOX for 7 days and then ENZ for 3 days were analyzed for cell viability. **P < 0.01, and ***P < 0.001.

MUC1-C is expressed as ~25 kDa glycosylated and 17 kDa unglycosylated proteins (25, 26). The MUC1-C 72 aa cytoplasmic domain (MUC1-CD) is an intrinsically disordered region that functions as a scaffold in integrating intracellular signaling pathways that, among others, include TCF4/β-catenin–driven MYC expression (Supplemental Figure 2A) (25, 27, 28). TCF4, β-catenin, and MYC, a driver of the NE phenotype (12, 29), were upregulated in LNCaP-ER versus LNCaP cells (Supplemental Figure 2B) and downregulated in LNCaP-ER cells with MUC1-C silencing (Supplemental Figure 2C), consistent with MUC1-C dependency in regulating MYC signaling. The 17 kDa MUC1-C protein localizes to chromatin as homodimers and higher order multimers, where it interacts with transcription factors, such as MYC, and effectors of the epigenome (25, 30, 31). Chromatin levels of MUC1-C, TCF4, β-catenin, and MYC were increased in LNCaP-ER versus LNCaP cells (Figure 2A). Furthermore, silencing MUC1-C in LNCaP-ER cells decreased expression of TCF4, β-catenin, and MYC in chromatin (Figure 2B). These results were verified by rescue of MUC1-C silencing with MUC1-CD (Figure 2C), supporting a central role for MUC1-C in regulating the MYC pathway. Gene set enrichment analysis (GSEA) further uncovered that silencing MUC1-C in LNCaP-ER cells and overexpression of MUC1-C in LNCaP/MUC1-C OE cells are associated with regulation of the HALLMARK MYC TARGETS V1 gene signature (Figure 2D). From these analyses, we found that expression of multiple glycolytic pathway genes encoding GLUT1, HK2, G6PD, PKM2, and LDHA were upregulated in LNCaP-ER versus LNCaP cells (Figure 2, E and F) and suppressed in LNCaP-ER cells with MUC1-C silencing using different MUC1 shRNAs (Figure 2, G and H, and Supplemental Figure 2D). By extension, analysis of glycolysis using the Seahorse assay that measures oxygen consumption rate (OCR) and extracellular acidification rate (ECAR) demonstrated that targeting MUC1-C genetically and pharmacologically increases the OCR/ECAR ratio in support of suppressing the glycolytic pathway (Figure 2I and Supplemental Figure 2, E and F). MUC1-C expression was also associated with regulation of the Gene Ontology Biological Process (GOBP) GLUCOSE METABOLIC PROCESS gene signature (Supplemental Figure 2G). DOX treatment of LNCaP-ER/tet-MYCshRNA cells further demonstrated that MYC was necessary for expression of glycolytic pathway genes (Figure 2, J and K). Moreover, we found that, like MUC1-C, MYC was necessary for conferring self-renewal capacity (Figure 2L) and ENZ resistance (Figure 2M). These results indicate that MUC1-C/MYC signaling drives the aerobic glycolytic pathway in association with the ENZ-resistant phenotype and CSC state.

### AR-negative DU-145 PC cells are dependent on MUC1-C for DTX resistance.

**Figure 3:**
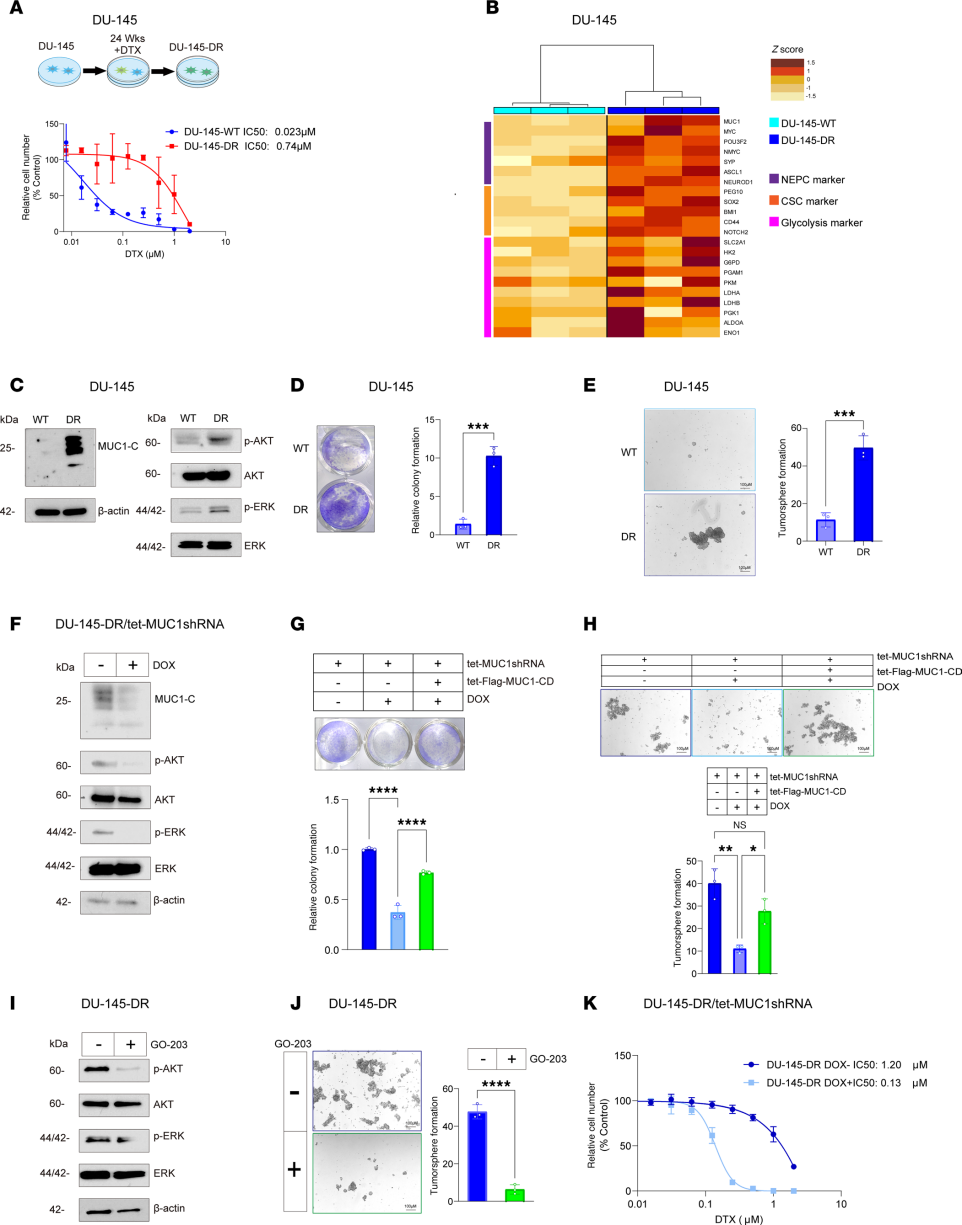
DU-145-DR cells are dependent on MUC1-C for DTX resistance and self-renewal capacity. (**A**) Parental DU-145 and DU-145-DR cells treated with DTX for 3 days were analyzed for cell viability. (**B**) Heatmap of NE, CSC, and glycolytic gene expression from qRT-PCR analysis of biological triplicates of DU-145 and DU-145-DR cells. (**C**) Immunoblot analysis of DU-145 and DU-145-DR cell lysates run at different times. (**D**) DU-145 and DU-145-DR cells were analyzed for colony formation. Representative photomicrographs of stained colonies (left). Results (mean ± SD of 3 determinations) expressed as relative colony number compared with DU-145 cells (assigned a value of 1) (*t* test; *n* = 3) (right). (**E**) DU-145 and DU-145-DR cells were analyzed for tumorsphere formation (*t* test; *n* = 3). (**F**) Immunoblot analysis of lysates from DU-145-DR/tet-MUC1shRNA cells treated with DOX for 7 days run contemporaneously in parallel. (**G**) DU-145-DR cells expressing tet-MUC1shRNA and/or tet-MUC1-CD vectors treated with vehicle or DOX for 7 days were analyzed for colony formation (*t* test; *n* = 3). (**H**) DU-145-DR cells expressing tet-MUC1shRNA and/or tet-MUC1-CD vectors were treated with vehicle or DOX for 7 days and analyzed for tumorsphere formation (*t* test; *n* = 3). (**I**) Lysates from DU-145-DR cells treated with vehicle or 3 μM GO-203 for 3 days were immunoblotted with antibodies against the indicated proteins run contemporaneously in parallel. (**J**) DU-145-DR cells treated with vehicle or 3 μM GO-203 for 3 days were analyzed for tumorsphere formation (*t* test; *n* = 3). (**K**) DU-145-DR/tet-MUC1shRNA cells treated with DOX for 7 days and then with DTX for 3 days were analyzed for cell viability. *P < 0.05, **P < 0.01, ***P < 0.001, and ****P < 0.0001.

Having identified a role for MUC1-C in conferring resistance of AR-positive LNCaP-ER cells, we asked if MUC1-C is of importance in drug-resistant AR-negative PC cells. To this end, we selected DU-145 cells for resistance to DTX (Figure 3A). Analysis of DU-145-DR versus DU-145 cells identified upregulation of MUC1-C in association with increases in expression of genes encoding (i) NE and CSC markers and (ii) glycolytic enzymes (Figure 3B). Analysis of DU-145-DR versus DU-145 cells also demonstrated increases in p-AKT and p-ERK levels (Figure 3C), colony formation (Figure 3D), and self-renewal capacity (Figure 3E). RNA-Seq in DU-145-DR cells further demonstrated that MUC1-C silencing was associated with downregulation of the HALLMARK G2M CHECKPOINT and HALLMARK E2F TARGETS gene signatures (Supplemental Figure 3, A–C). Silencing MUC1-C in DOX-treated DU-145-DR/tet-MUC1shRNA cells suppressed the increases in p-AKT and p-ERK (Figure 3F), as well as colony formation (Figure 3G) and self-renewal (Figure 3H), which were rescued with MUC1-CD expression. In support of these results, treatment of DU-145-DR cells with GO-203 downregulated p-AKT and p-ERK (Figure 3I) and inhibited self-renewal capacity (Figure 3J). Additionally, silencing MUC1-C with different MUC1shRNAs attenuated resistance of DU-145-DR cells to DTX treatment (Figure 3K and Supplemental Figure 3D). These findings in DU-145-DR cells indicate that MUC1-C is necessary for survival, self-renewal, and the DTX-resistant phenotype.

### MUC1-C regulates MYC and the glycolytic pathway in DU-145-DR cells.

**Figure 4:**
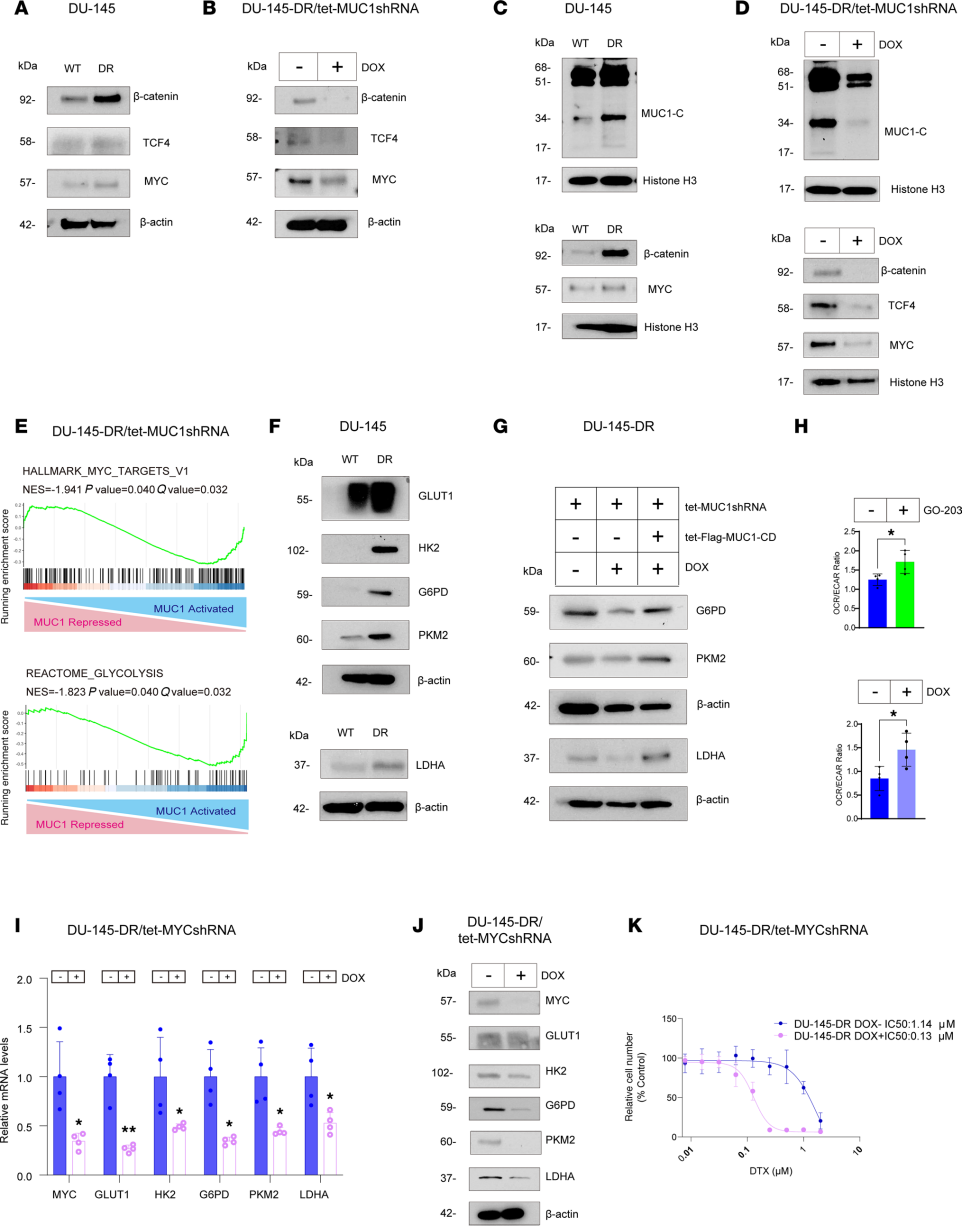
DU-145-DR cells are dependent on the MUC1-C/MYC axis for regulation of glycolytic enzyme expression and DTX resistance. (**A**) Immunoblot analysis of DU-145 and DU-145-DR cell lysates run contemporaneously in parallel. (**B**) Immunoblot analysis of lysates from DU-145-DR/tet-MUC1shRNA cells treated with DOX for 7 days run contemporaneously in parallel. (**C**) Immunoblot analysis of chromatin from DU-145 and DU-145-DR cells run at different times. (**D**) Immunoblot analysis of chromatin from DU-145-DR/tet-MUC1shRNA cells treated with DOX for 7 days run at different times. (**E**) GSEA of RNA-Seq from DU-145-DR cells with MUC1-C silencing using the HALLMARK MYC TARGETS and REACTOME GLYCOLYSIS gene signatures. (**F**) Immunoblot analysis of DU-145 and DU-145-DR cell lysates run at different times. (**G**) Immunoblot analysis of lysates from DU-145-DR cells expressing the indicated vectors and treated with DOX for 7 days run at different times. (**H**) DU-145-DR cells treated with vehicle or 3 μM GO-203 (upper) and DU-145-DR/tet-MUC1shRNA cells treated with vehicle or DOX for 7 days (lower) were assayed for OCR and ECAR. The OCR/ECAR results (mean ± SD of 4 determinations) are expressed as the relative ratio compared with untreated cells (*t* test; *n* = 3). (**I**) DU-145-DR/tet-MYCshRNA cells treated with DOX for 7 days were analyzed for the indicated transcripts by qRT-PCR (*t* test; *n* = 3). (**J**) Immunoblot analysis of lysates from DU-145-DR/tet-MYCshRNA cells treated with DOX for 7 days run contemporaneously in parallel. (**K**) DU-145-DR/tet-MYCshRNA cells treated with DOX for 7 days and then DTX for 3 days were analyzed for cell viability. *P < 0.05, and **P < 0.01.

Comparison of DU-145-DR versus DU-145 cells demonstrated that upregulation of MUC1-C was associated with increases in TCF4, β-catenin, and MYC (Figure 4A), which were suppressed by silencing MUC1-C in DOX-treated DU-145-DR/tet-MUC1shRNA cells (Figure 4B). Increases in chromatin levels of MUC1-C, TCF4, β-catenin, and MYC in DU-145-DR versus DU-145 cells (Figure 4C) were also suppressed in DU-145-DR cells with MUC1-C silencing (Figure 4D). GSEA of RNA-Seq data from DU-145-DR cells with MUC1-C silencing further demonstrated that MUC1-C was associated with regulation of the HALLMARK MYC TARGETS V1 and REACTOME GLYCOLYSIS (Figure 4E) gene signatures. Mirroring effects of the MUC1-C/MYC pathway in LNCaP-ER cells, we found that increases in MUC1-C in DU-145-DR versus DU-145 cells were associated with upregulation of GLUT1, HK2, G6PD, PKM2, and LDHA levels (Figure 4F). Silencing MUC1-C with different MUC1 shRNAs downregulated expression of these glycolytic enzymes (Supplemental Figure 4, A and B), which were rescued with MUC1-CD (Figure 4G). Targeting MUC1-C with GO-203 similarly suppressed these effectors of glycolysis (Supplemental Figure 4, C and D). Analysis of glycolysis using the Seahorse assay further demonstrated that targeting MUC1-C genetically and pharmacologically increased the OCR/ECAR ratio, in concert with suppression of the glycolytic pathway (Figure 4H and Supplemental Figure 4, E and F). Moreover, treatment of DU-145-DR/tet-MYCshRNA cells with DOX demonstrated that silencing MYC suppressed glycolytic enzyme expression (Figure 4, I and J). Of additional importance, we found that, like MUC1-C, silencing MYC sensitized DU-145-DR cells to DTX treatment (Figure 4K). These findings indicate that the MUC1-C/MYC axis regulated the glycolytic pathway in association with conferring the DU-145 DTX-resistant phenotype.

### MUC1-C regulates redox balance in association with conferring drug resistance.

**Figure 5:**
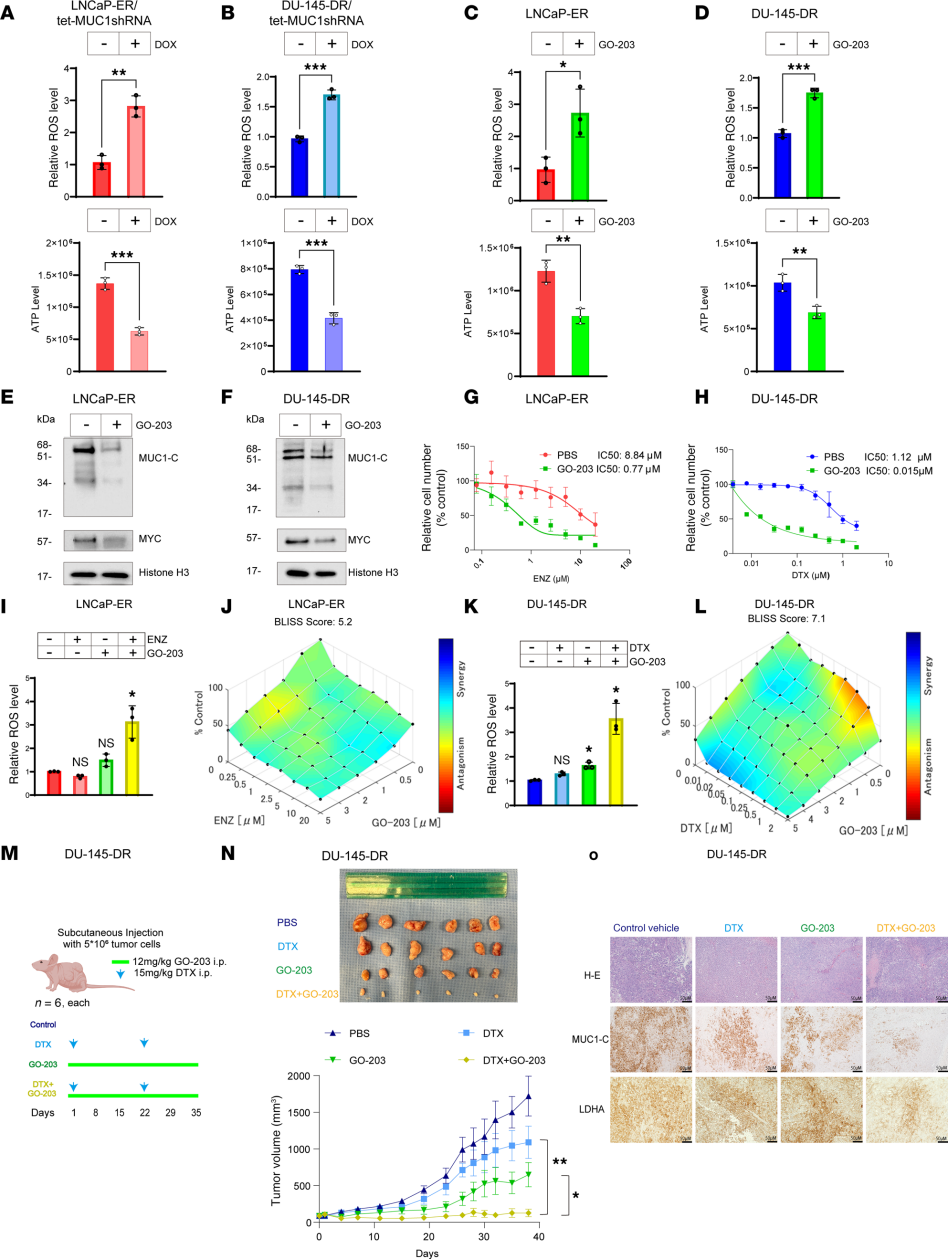
MUC1-C integrates redox balance and the drug-resistant phenotype. (**A** and **B**) LNCaP-ER/tet-MUC1shRNA (**A**) and DU-145-DR/tet-MUC1shRNA cells treated with DOX for 7 days (**B**) were analyzed for ROS and ATP levels. The results (mean ± SD of 3 determinations) are expressed as (i) relative ROS levels compared with vehicle-treated cells (assigned a value of 1) and (ii) absolute ATP levels as determined by luminescence (*t* test; *n* = 3). (**C** and **D**) LNCaP-ER (**C**) and DU-145-DR (**D**) cells treated with 3 μM GO-203 for 3 days were analyzed for ROS and ATP levels (*t* test; *n* = 3). (**E** and **F**) Immunoblot analysis of chromatin from LNCaP-ER (**E**) and DU-145-DR (**F**) cells treated with 3 μM GO-203 for 3 days run contemporaneously in parallel. (**G** and **H**) LNCaP-ER (**G**) and DU-145-DR (**H**) cells treated with 3 μM GO-203 and ENZ or DTX were analyzed for cell viability. (**I**) LNCaP-ER cells treated with 10 μM ENZ, 3 μM GO-203, and the combination of GO-203 and ENZ for 3 days were analyzed for ROS levels. (**J**) LNCaP-ER cells were treated with GO-203 and ENZ for 3 days. Indicated are the combination indices determined using ΔBliss scores. (**K**) DU-145-DR cells treated with 10 μM DTX, 3 μM GO-203, and the combination of GO-203 and DTX for 3 days were analyzed for ROS levels. (**L**) DU-145-DR cells treated with GO-203 and DTX for 3 days. Indicated are the combination indices determined using ΔBliss scores. (**M**) Treatment schedule for castrated nude mice with 100 mm^3^ DU-145-DR tumors. (**N**) Tumor volumes (mean ± SD) at the end of the study were (i) PBS, 1,720.9 ± 607.3 mm^3^; (ii) DTX, 1,090.5 ± 493.5 mm^3^; (iii) GO-203, 648.7 ± 371.5 mm^3^; and (iv) DTX+GO-203, 126.9 ± 116.9 mm^3^. (**O**) Representative IHC images of DU-145-DR tumors treated with DTX, GO-203, and GO-203+DTX and stained with H&E and for MUC1-C and LDHA. Scale bars, 50 μm. *P < 0.05, **P < 0.01, and ***P < 0.001.

Aerobic glycolysis regulates redox balance and ATP production to maintain the CSC state (32, 33). In investigating the relationship between MUC1-C–induced aerobic glycolysis and drug resistance, we found that silencing MUC1-C in LNCaP-ER (Figure 5A) and DU-145-DR (Figure 5B) cells increased ROS and decreased ATP levels. In support of MUC1-C dependence, similar effects on ROS and ATP levels were obtained in these drug-resistant models when targeting MUC1-C with GO-203 treatment (Figure 5, C and D). GO-203 also decreased MUC1-C and MYC levels in chromatin (Figure 5, E and F) and attenuated resistance of LNCaP-ER to ENZ (Figure 5G) and DU-145-DR cells to DTX (Figure 5H). These results indicated that the effects of targeting MUC1-C/MYC signaling on the glycolytic pathway and ROS levels contributed to the drug-resistant phenotype. In addressing this potential relationship, GO-203–treated LNCaP-ER and DU-145-DR cells were incubated with the antioxidant glutathione, which (i) reversed the effects on ROS and ATP levels (Supplemental Figure 5, A and B) and (ii) restored drug resistance (Supplemental Figure 5, C and D). As an additional control, treatment of LNCaP-ER and DU-145-DR cells with 2-deoxyglucose (2-DG), an inhibitor of glycolysis, increased ROS and decreased ATP levels (Supplemental Figure 5, E and F) and abrogated the respective drug-resistant phenotypes (Supplemental Figure 5, G and H). Furthermore, we found in LNCaP-ER cells that (i) combining GO-203 with ENZ increased ROS levels to a greater extent than either agent alone (Figure 5I) and (ii) the combination was synergistic in suppressing survival (Figure 5J and Supplemental Figure 5I). These results were extended to DU-145-DR cells; that is, combining GO-203 and DTX was more effective than either agent alone in increasing ROS levels (Figure 5K) and inducing loss of survival (Figure 5L and Supplemental Figure 5J). In an in vivo model, resistance of established DU-145-DR tumor xenografts to DTX was reversed by targeting MUC1-C with GO-203 treatment (Figure 5, M and N). As assessed by IHC tumor staining, we found that MUC1-C and LDHA levels were decreased to a greater extent with the combination of GO-203 and DTX treatment (Figure 5O). These findings indicate that MUC1-C integrates regulation of the glycolytic pathway and redox balance with conferring drug resistance.

### MUC1-C regulates redox balance and drug resistance in H660 t-NEPC cells.

**Figure 6:**
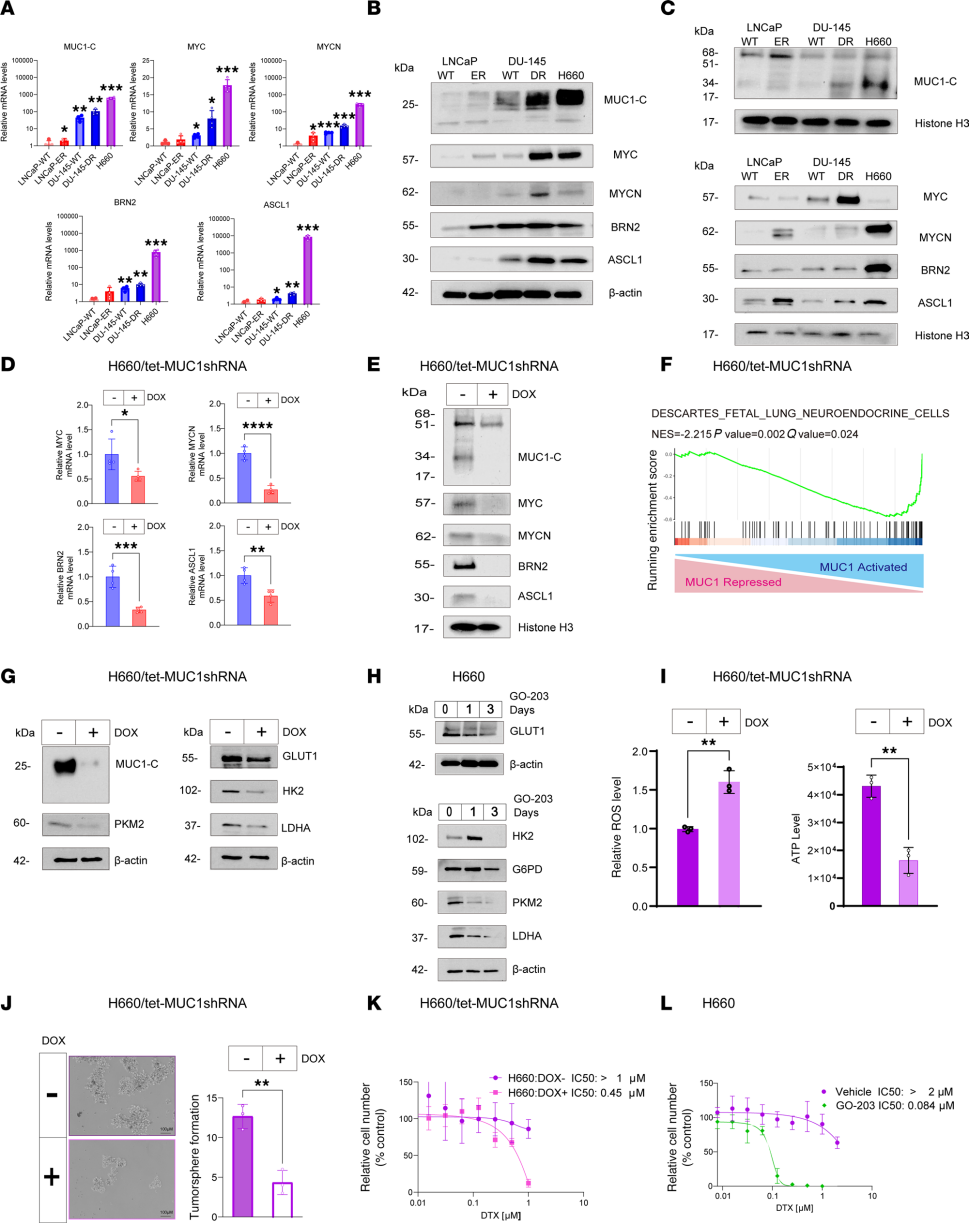
MUC1-C regulates redox balance, the NE phenotype, and drug resistance in H660 t-NEPC cells. (**A**) Cells were analyzed for the indicated transcripts by qRT-PCR. The results (mean ± SD of 4 determinations) are expressed as relative levels compared with that obtained for LNCaP cells (assigned a value of 1) (*t* test; *n* = 3). (**B**) Immunoblot analysis of cell lysates run contemporaneously in parallel. (**C**) Immunoblot analysis of chromatin run at different times. (**D**) H660/tet-MUC1shRNA cells treated with DOX for 7 days were analyzed for the indicated transcripts by qRT-PCR (*t* test; *n* = 3). (**E**) Immunoblot analysis of chromatin from H660/tet-MUC1shRNA cells treated with DOX for 7 days run contemporaneously in parallel. (**F**) RNA-Seq was performed in triplicate on H660/tet-MUC1shRNA cells treated with DOX for 7 days. GSEA was performed using the DESCARTES FETAL LUNG NEUROENDOCRINE CELLS gene signature. (**G**) Immunoblot analysis of lysates from H660/tet-MUC1shRNA cells treated with DOX for 7 days were immunoblotted run at different times. (**H**) Immunoblot analysis of lysates from H660 cells treated with 3 μM GO-203 run at different times. (**I**) H660/tet-MUC1shRNA cells treated with DOX for 7 days were analyzed for ROS and ATP levels (*t* test; *n* = 3). (**J**) H660/tet-MUC1shRNA cells treated with DOX for 7 days were analyzed for tumorsphere formation (*t* test; *n* = 3). (**K**) H660/tet-MUC1shRNA cells treated with DOX for 7 days and then DTX for 3 days were analyzed for cell viability. (**L**) H660 cells treated with 3 μM GO-203 for 3 days and then DTX for 3 days were analyzed for cell viability. *P < 0.05, **P < 0.01, ***P < 0.001, and ****P < 0.0001.

Drug-resistant CRPC can progress to the highly aggressive form of treatment-related t-NEPC, which has limited therapeutic options and poor clinical outcomes (6–8, 34). To determine whether MUC1-C–mediated regulation of ROS and ATP balance contributes to the refractory t-NEPC phenotype, we studied AR-negative NEPC NCI-H660 cells, which express high levels of NE markers and exhibit other NEPC characteristics (35). Compared with LNCaP-ER and DU-145-DR cells, H660 cells exhibited significantly higher levels of MUC1-C, MYC, MYCN, BRN2, and ASCL1 transcripts (Figure 6A). Analysis of total cell lysates (Figure 6B) and chromatin (Figure 6C) demonstrated increased MUC1-C expression in association with varying levels of MYC, MYCN, BRN2, and ASCL1. Targeting MUC1-C genetically in H660 cells downregulated (i) p-AKT and p-ERK and (ii) TCF4, β-catenin, and MYC expression (Supplemental Figure 6, A and B). MUC1-C was also necessary for expression of MYC, MYCN, BRN2, and ASCL1 transcripts (Figure 6D) and proteins in chromatin (Figure 6E). GSEA of RNA-Seq data from H660 cells with MUC1-C silencing was associated with downregulation of the HALLMARK MYC TARGETS V1 (Supplemental Figure 6C) and DESCARTES FETAL LUNG NEUROENDOCRINE CELLS (Figure 6F) gene signatures. Silencing MUC1-C in H660 cells was also associated with suppression of the REACTOME GLYCOLYSIS gene signature (Supplemental Figure 6D). Based on these results, we verified that targeting MUC1-C in H660 cells with silencing (Figure 6G) and GO-203 (Figure 6H) downregulated expression of the GLUT1, HK2, G6PD, PKM2, and LDHA proteins. Furthermore, targeting MUC1-C in H660 cells (i) increased ROS and decreased ATP levels (Figure 6I), (ii) suppressed self-renewal (Figure 6J), and (iii) attenuated resistance to DTX treatment (Figure 6, K and L). These findings indicate that MUC1-C activates MYC and the glycolytic pathway in conferring resistance of H660 NEPC cells to DTX.

### Drug-resistant patient-derived NEPC cells are sensitive to targeting MUC1-C with an ADC.

**Figure 7:**
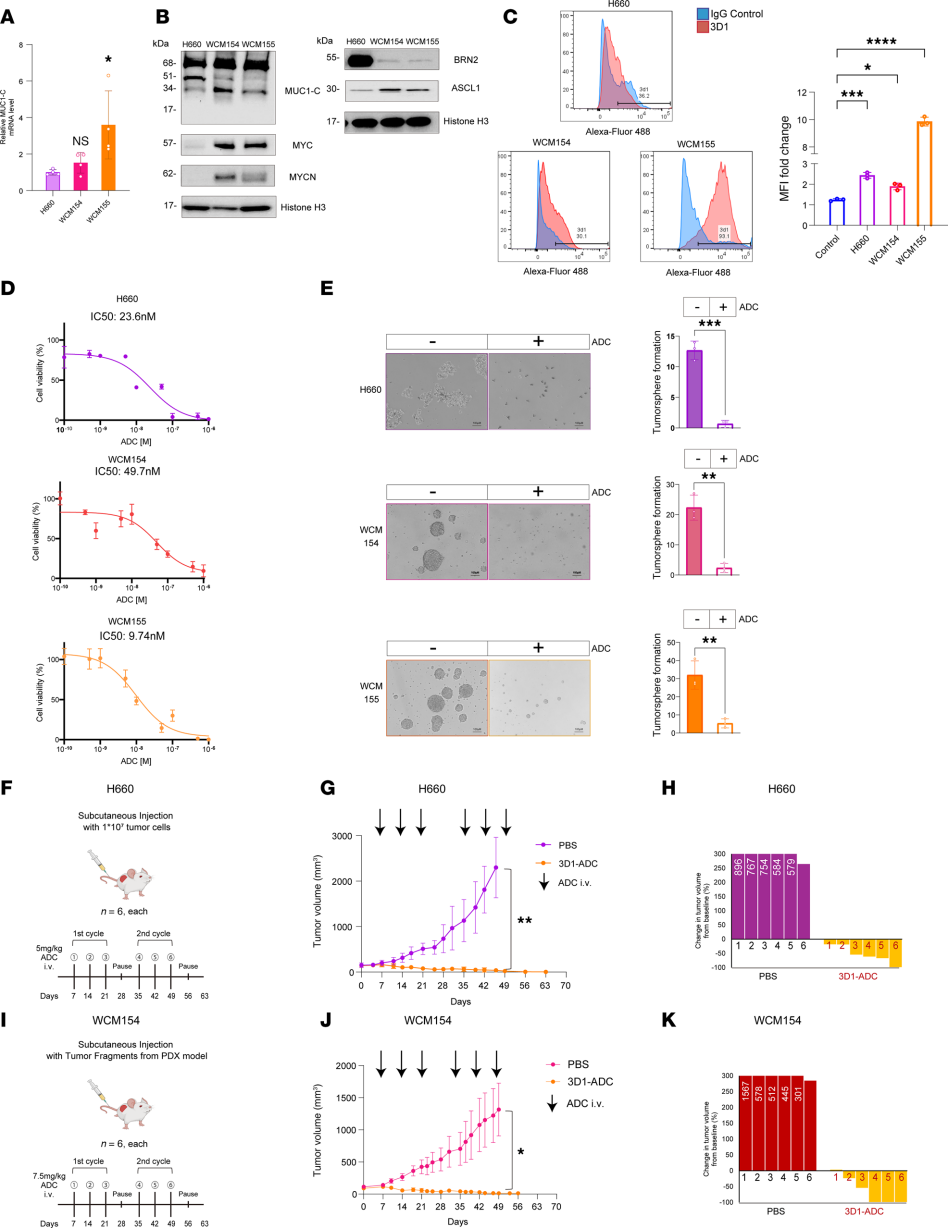
Targeting MUC1-C with an ADC is effective against t-NEPC cells growing in vitro and as tumor xenografts. (**A**) Cells were analyzed for the indicated transcripts by qRT-PCR. The results (mean ± SD of 4 determinations) are expressed as relative levels compared with that obtained for H660 cells (assigned a value of 1) (*t* test; *n* = 3). (**B**) Immunoblot analysis of chromatin from H660, WCM154, and WCM155 cells run at different times. (**C**) Cells were analyzed by flow cytometry with a control IgG and MAb-3D1. Shown are histograms and percentage of positive for MUC1-C expression (left). The bar plot depicts MFI fold-change (MAb-3D1/IgG). The results (mean ± SD of 3 determinations) are expressed as relative levels compared with that obtained for 3D1-negative control cells (assigned a value of 1) (right). (**D**) Cells treated with PBS or M1C ADC for 7 days were analyzed for cell viability. (**E**) Cells treated with 50 nM M1C ADC for 7 days were analyzed for tumorsphere formation (*t* test; *n* = 3). (**F**) Treatment schedule for NSG mice with 100 mm^3^ H660 tumors. (**G**) Tumor volumes (mean ± SD) on day 42 were 1,810.9 ± 467.0 mm^3^ in the PBS group and 47.5 ± 41.3 mm^3^ in the M1C ADC group (*P* value = 0.001). (**H**) Percentage change in volume from baseline shown as a waterfall plot. (**I**) Treatment schedule for NOD/SCID-γ mice with 100 mm^3^ WCM154 patient-derived xenograft (PDX) tumors. (**J**) Tumor volumes (mean ± SD) for 6 mice on day 44 were 1,152.6 ± 384.9 mm^3^ in the PBS control group and 28.4 ± 32.2 mm^3^ in the M1C ADC group (*P* value = 0.018). (**K**) Percentage change in volume for each WCM154 tumor from baseline shown as a waterfall plot. Scale bar, 100 μm. *P < 0.05, **P < 0.01, ***P < 0.001, and ****P < 0.0001.

Treatment-resistant CRPC that progresses to t-NEPC has limited therapeutic options (6–8, 34). Therefore, having identified MUC1-C dependence of DU-145-DR and H660 cells, we studied WCM154 and WCM155 organoids derived from patients with t-NEPC (5, 36). Analysis of WCM154 and WCM155 cells demonstrated that MUC1-C expression as assessed by mRNA (Figure 7A) and chromatin protein levels (Figure 7B) were comparable to those in H660 cells. Levels of MYC transcripts (Supplemental Figure 7A) and protein in chromatin (Figure 7B) were increased in WCM154 and WCM155 cells; however, BRN2 levels were relatively higher in H660 cells (Figure 7B). Of translational relevance, MUC1-C expression as determined by flow cytometry was also detectable on the surface of H660, WCM154, and WCM155 cells (Figure 7C). There are no clinically approved agents against MUC1-C; accordingly, we generated MAb 3D1 against the MUC1-C extracellular domain alpha-3 helix (37). Humanized huMAb 3D1 was conjugated to monomethyl auristatin E using a maleimidocaproyl-valine-citrulline-*p*-aminobenzyloxycarbonyl cleavable linker at a drug/antibody ratio of ~4 (37). The M1C ADC was active against MUC1-positive, but not MUC1-negative, breast and lung cancer cells (37); however, it is not known if this agent has activity against CRPC/NEPC cells, particularly in the setting of drug resistance. In vitro sensitivity to the M1C ADC was comparable for H660, WCM154, and WCM155 cells with IC_50_ values of 23.6, 49.7, and 9.74 nM, respectively (Figure 7D). Importantly, M1C ADC treatment of H660, WCM154, and WCM155 cells suppressed self-renewal capacity (Figure 7E). Moreover, treatment of established H660 tumor xenografts with 2 cycles of the M1C ADC administered intravenously at 5 mg/kg/w 3 weeks every 28 days was highly effective in inducing complete and prolonged responses (Figure 7, F–H) in the absence of weight loss and other overt toxicities (Supplemental Figure 7B). Additionally, as found for H660 xenografts, M1C ADC treatment of established WCM154 tumors was highly effective in inducing complete and durable responses (Figure 7, I–K) without significant toxicity (Supplemental Figure 7C). These findings support dependency of t-NEPC cells on MUC1-C for self-renewal and tumorigenicity and identify the M1C ADC as a potential therapeutic for the treatment of patients with refractory NEPC.

### Analysis of scRNA-Seq data identifies MUC1-C as a target associated with driving progression of treatment-resistant CRPC/NEPC.

**Figure 8:**
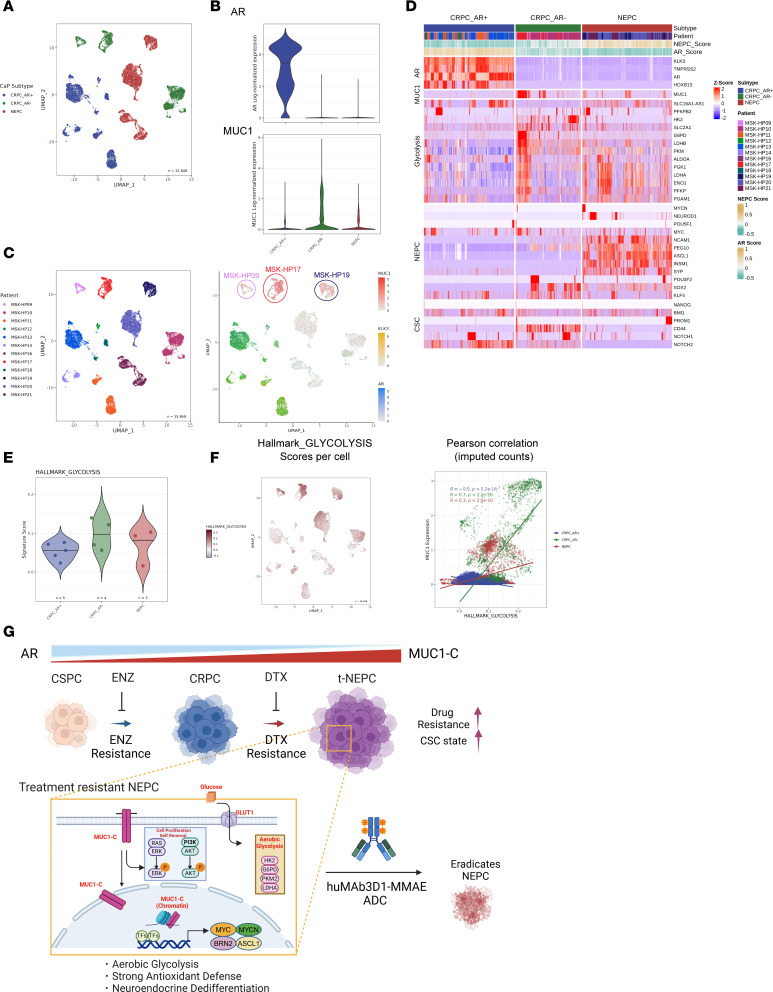
Analysis of scRNA-Seq data derived from treatment-resistant CRPCs and NEPCs identifies MUC1 associations with glycolysis, stemness, and NE differentiation. (**A**) Uniform manifold approximation and projection (UMAP) of scRNA-Seq data from patient treatment-resistant CRPC/AR^+^, CRPC/AR^–^, and NEPC cells. (**B**) AR and MUC1 expression by CRPC/AR^+^, CRPC/AR^–^, and NEPC subtypes. Median expression per cluster is shown as a horizontal line. (**C**) UMAP of scRNA-Seq data from the indicated patient tumor samples (left). Overlap of MUC1, AR, and KLK3 normalized gene expression (right). (**D**) Heatmaps depicting CRPC/AR^+^, CRPC/AR^–^, and NEPC cell expression of candidate genes associated with AR signaling, glycolysis, NEPC and CSC gene signatures. (**E**) Gene set enrichment was performed for CRPC/AR^+^, CRPC/AR^–^, and NEPC cells using the HALLMARK GLYCOLYSIS gene signature. Each point represents the average score per tumor. Median gene signature scores per cluster are shown as horizontal lines. (**F**) UMAP showing the scores per cell using the HALLMARK_GLYCOLYSIS gene signature (left). Pearson’s correlation plots for CRPC/AR^+^, CRPC/AR^–^, and NEPC cells using imputed gene expression of MUC1 and HALLMARK GLYCOLYSIS enrichment scores (right). (**G**) Depiction of MUC1-C dependence in treatment-resistant CRPC/NEPC. Selection of AR-positive LNCaP cells for ENZ resistance and AR-negative DU-145 cells for DTX resistance induces MUC1-C expression and dependence on MUC1-C for the drug-resistant phenotype. Consistent with MUC1-C suppression of AR (12), progression of ENZ- and DTX-resistant PC to t-NEPC is associated with increasing MUC1-C and decreasing AR levels. Progression of drug-resistant PC to t-NEPC is dependent on activation of the MUC1-C/MYC axis and thereby effectors of the glycolytic pathway that regulate ROS and ATP levels necessary for maintaining the CSC state and treatment resistance. In support of MUC1-C addiction, we demonstrate that an M1C ADC is highly effective against t-NEPC cell self-renewal and tumorigenicity.

To extend the above results to clinical samples, we analyzed a single-cell RNA-Seq (scRNA-Seq) dataset derived from treatment-resistant CRPCs and NEPCs (38). Consistent with involvement of MUC1-C in suppressing AR expression, we found that MUC1 was upregulated in association with downregulation of AR in CRPC/AR^–^ and NEPC, as compared with CRPC/AR^+^, tumors (Figure 8, A and B). For individual patient samples, we overlapped normalized MUC1, AR, and KLK3 gene expression and found that (i) CRPC/AR^+^ tumors have high AR and KLK3 versus low MUC1 levels, and (ii) MSK-HP09, MSK-HP17, and MSK-HP19 samples with high MUC1 expression are negative for AR and KLK3 (Figure 8C). Heatmaps of CRPC/AR^–^ and NEPC versus CRPC/AR^+^ tumors further demonstrated that upregulation of MUC1 associated with suppression of AR and KLK3 expression and induction of genes encoding (i) glycolytic enzymes; (ii) effectors of the CSC state, such as NOTCH1/2 and CD44; and (iii) markers of NE differentiation (Figure 8D). As further support for involvement of MUC1 in activating glycolysis, we verified by gene set variation analysis (GSVA) that MUC1 associated with glycolytic genes in the progression of treatment-resistant CRPC/AR^–^ and NEPC tumors (Figure 8, E and F). These findings uncover an association of MUC1 with glycolysis, stemness, and NE differentiation in refractory CRPC/NEPC and support targeting MUC1-C in this advanced patient population.

## Discussion

Patients with advanced hormone-sensitive PC treated with androgen deprivation therapy with or without second-generation AR pathway inhibitors invariably develop castration resistance. Subsequent standard therapy is often AR blockade with drugs, like ENZ, or chemotherapy with DTX-based regimens. Despite initial responses, acquired resistance also develops against these therapeutic approaches. Resistance is mediated through diverse mechanisms involving AR reactivation, AR mutations, expression of AR splice variants, and activation of glucocorticoid receptor and WNT signaling pathways, among others, and in some cases loss of AR expression and AR signaling dependence (39). The pleiotropic nature of these mechanisms limits subsequent therapeutic approaches. There is no known association of the MUC1-C protein, which is upregulated in CRPC/NEPC, with treatment resistance in advanced PC (26). The present work demonstrates that selection of AR-positive LNCaP cells for ENZ resistance is associated with increases in MUC1-C expression. Functionally, targeting MUC1-C genetically or pharmacologically reversed ENZ resistance. Furthermore, LNCaP cells overexpressing exogenous MUC1-C exhibited ENZ resistance, verifying that MUC1-C is necessary for the refractory phenotype. MUC1-C suppresses the AR axis (12), which could explain the mechanism for ENZ resistance. We therefore asked if MUC1-C confers resistance of AR-null DU-145 cells. Here, we selected for resistance to DTX, which like cabazitaxel and platinum-based chemotherapy is used for the treatment of AR-negative CRPC. We found that DTX-resistant DU-145-DR cells have increased MUC1-C levels and are dependent on MUC1-C for the refractory phenotype. Treatment-resistant CRPC can progress to t-NEPC (5–8). Accordingly, we studied H660 t-NEPC cells and found that their resistance to DTX is also MUC1-C dependent. These findings indicate that MUC1-C functions as a common effector of resistance in (i) LNCaP-ER cells to ENZ and (ii) DU-145-DR and H660 cells to DTX (Figure 8G).

Having found that MUC1-C is necessary for resistance of PC cells across the spectrum of progression and treatment with ENZ and DTX, we searched for a MUC1-C–driven pathway that might extend from CRPC to t-NEPC. Along these lines, MUC1-C drives the CSC state (25, 26), which is characterized by self-renewal capacity, lineage plasticity, and treatment resistance (40–43). Our results demonstrate that drug-resistant LNCaP-ER, DU-145-DR, and H660 cells are each dependent on MUC1-C for tumorsphere formation as a measure of self-renewal capacity and the CSC state. Other shared pathways were activation of (i) ERK and AKT signaling, (ii) E2F and G2M gene signatures, and (iii) importantly, effectors of glycolysis. CSCs are dependent on aerobic glycolysis for generating ATP and regulating redox balance, which are both essential for maintaining self-renewal capacity (44). MUC1-C binds directly to MYC and regulates MYC target genes that include those encoding glycolytic enzymes. Our results in drug-resistant PC models demonstrate that the MUC1-C/MYC axis is essential for activating the glycolytic pathway, sustaining ATP levels, and circumventing increases in ROS. Maintaining low ROS levels in CSCs through activation of glycolysis is necessary for supporting CSC self-renewal and cell cycle progression, as well as reducing oxidative DNA damage and sensitivity to genotoxic agents (44–47). As evidence that LNCaP-ER, DU-145-DR, and H660 cells are indeed dependent on MUC1-C–driven regulation of ROS levels, the effects of targeting MUC1-C on self-renewal and drug resistance were reversed by attenuating dysregulation of redox balance with an antioxidant.

Our findings that CRPC and NEPC cells are addicted to MUC1-C for the CSC state, as evidenced by self-renewal and drug resistance, supported the importance of MUC1-C as a potential target for refractory PC treatment. ADCs have effectively changed the treatment landscape of breast and other cancers (48); whereas, despite a number of ongoing clinical trials, there are no approved ADCs as yet for advanced PC (49). The present studies demonstrate that an M1C ADC is highly effective against established H660 tumor xenografts with complete and long-term responses. These provocative results were extended by the demonstration that patient-derived t-NEPC xenograft models are also highly sensitive to the M1C ADC in vitro and in vivo. The M1C ADC is presently under development by the National Institutes of Health National Cancer Institute Experimental Therapeutics Program (NCI NExT Program) for Investigational New Drug–enabling studies. Our findings thus demonstrate that MUC1-C is (i) a common effector of drug-resistant CRPC and t-NEPC cell progression and (ii) a druggable target with an M1C ADC for the treatment of patients with refractory PCs.

## Methods

### Sex as a biological variable.

The present studies were focused on PC. Sex was not considered as a biological variable.

### Cell culture.

Human LNCaP and DU-145 cells (ATCC) were cultured in RPMI 1640 medium (Thermo Fisher Scientific) supplemented with 10% FBS. Human NCI-H660 NEPC cells (ATCC) were cultured in RPMI 1640 medium with 5% FBS, 10 nM β-estradiol (MilliporeSigma), 10 nM hydrocortisone, 1% insulin-transferrin-selenium (Thermo Fisher Scientific), and 2 mM l-glutamine (Thermo Fisher Scientific). Cells were treated with GO-203, ENZ, or DTX (Selleck Chemicals). LNCaP cells were treated with increasing ENZ concentrations for 24 weeks to select for ENZ-resistant LNCaP-ER cells. DU-145 cells were treated with increasing DTX concentrations for 24 weeks to select for DTX-resistant DU-145-DR cells. Human NEPC WCM154 and WCM155 organoids were maintained as described (5, 36). The M1C ADC was provided by the NCI NExT Program (Gaithersburg, Maryland, USA). Authentication of the cells was performed by short tandem repeat analysis. Cells were monitored for mycoplasma contamination using the MycoAlert Mycoplasma Detection Kit (Lonza).

### Cell viability assays.

Cells were seeded at a density of 5,000 cells per well in 96-well plates. After 24 hours, the cells were treated with different concentrations of ENZ, DTX, GO-203, and anti–MUC1-C ADC. Cell viability was assessed using the Alamar blue assay (Thermo Fisher Scientific) in sextuplicate wells as described (30). The IC_50_ value was determined by nonlinear regression of the dose-response data using Prism 9.0 (GraphPad Software). Fluorescence intensity (560 nm excitation/590 nm emission) was measured in sextuplicate cells. The results (mean ± SD of 4 or 6 determinations) are expressed as relative cell number (% control) compared with that for control cells.

### Gene silencing and rescue vectors.

MUC1shRNA (MISSION shRNA TRCN0000122938; MilliporeSigma), MYCshRNA (MISSION shRNA TRCN0000039642; MilliporeSigma), or a control scrambled shRNA (CshRNA; MilliporeSigma) was inserted into the pLKO-tet-puro vector (Plasmid #21915; Addgene) as described (12). MUC1shRNA#2 (MISSION shRNA TRCN0000430218) was produced in HEK293T cells (ATCC) as described (30). MUC1-C was inserted into pHR′ CMV GFP Hygro vector (Plasmid #14858, Addgene) as described (50). Flag-tagged MUC1-CD was inserted into the empty control pLenti CMV Blast Dest (706-1) vector (Plasmid #17451, Addgene). Cells transduced with the vectors were selected for growth in 1–2 μg/mL puromycin. Cells were treated with 0.1% DMSO as the vehicle control or 500 ng/mL DOX (MilliporeSigma).

### qRT-PCR.

Total cellular RNA was isolated using TRIzol reagent (Thermo Fisher Scientific). cDNAs were synthesized using the High Capacity cDNA Reverse Transcription Kit (Applied Biosystems) as described (30). The cDNA samples were amplified using the Power SYBR Green PCR Master Mix (Applied Biosystems) and the CFX96 Real-Time PCR System (Bio-Rad) as described (30, 51). Primers used for qRT-PCR are listed in Supplemental Table 1. The results (mean ± SD of 3 or 4 determinations) are expressed as relative mRNA levels compared with that obtained for control cells (assigned a value of 1).

### Immunoblot analysis.

Total lysates prepared from subconfluent cells were subjected to immunoblot analysis as described (30, 51) using anti–MUC1-C (MA5-11202, 1:200 dilution; Invitrogen), anti-MYC (9402, 1:1,000 dilution; Cell Signaling Technology [CST]), anti-MYCN (9405, 1:1,000 dilution; CST), anti-BRN2 (12137, 1:1,000 dilution; CST), anti-ASCL1 (GTX129189, 1:1,000 dilution; GeneTex), anti-GLUT1 (115730, 1:1,000 dilution; Abcam), anti-HK2 (2867, 1:1,000 dilution; CST), anti-G6PD (8866s, 1:1,000 dilution; CST), anti-PKM2 (15822-1 AP, 1:1,000 dilution; Proteintech), anti-LDHA (3582s, 1:1,000 dilution; CST), anti–β-catenin (9587s, 1:1,000 dilution; CST), anti-TCF4 (22337-1-AP, 1:500 dilution; Proteintech), anti-AKT (9272, 1:1,000 dilution; CST), anti–phospho-AKT (4058s, 1:2,000 dilution; CST), p44/42 MAPK (ERK1/2) (9107s, 1:2,000 dilution; CST), anti–phospho-p44/42 MAPK (ERK1/2) (4377, 1:1,000 dilution; CST), and anti–β-actin (A5441, 1:5,000 dilution; Sigma-Aldrich). Chromatin analyzed by immunoblotting was isolated using the Chromatin Extraction Kit (ab117152, Abcam) according to the manufacturer’s instructions.

### RNA-Seq analysis.

Total RNA from cells cultured in triplicate was used to generate RNA-Seq datasets as described (30). Raw sequencing reads were aligned as described (30). Raw feature counts were normalized and analyzed using DESeq2 (SCR_015687) as described (30). Differential expression rank order was performed using GSEA as described (30). GSVA was performed using the GSVA package. Gene sets queried included those from the Hallmark, Reactome, and GO-BP gene sets available through the Molecular Signatures Database (MSigDB).

### Isobologram analysis.

A total of 5,000 were seeded per well in 96-well plates (Thermo Fisher Scientific) and incubated for 24 hours. The cells were then left untreated or treated with a maximum of 5 μM GO-203 in the presence of varying concentrations of ENZ or DTX for 72 hours. Cell viability as assessed by Alamar blue staining was used to calculate the effects of ENZ or DTX at each concentration of GO-203 by isobologram analysis. For synergy determination, the Bliss independence model was used to examine the synergistic effect (52, 53). In this model, when the experimentally determined drug combination effect is equal to, higher than, or lower than the expected effect (E_AB_), the combination is deemed additive (ΔBliss = 0), synergistic (>0), or antagonistic (<0), respectively.

### Flow cytometry.

Cells were blocked by incubation with 1% BSA/PBS for 20 minutes on ice. After washing with ice-cold PBS, cells were incubated with 40 μg/mL mAb 3D1 or 40 μg/mL IgG1-κ isotype control antibody (catalog 60070.1; STEMCELL Technologies) for 60 minutes on ice as described (51). FITC-conjugated goat F(ab′)_2_ anti-mouse immunoglobulin was used as the secondary antibody (ab150113, 1:1,000 dilution, Abcam). Cells were analyzed by MACSQuant Analyzer 10 Flow Cytometer (Miltenyi Biotec). Measurement of geometric MFI was performed with FlowJo v10.6.2 (BD Biosciences) software as described (51).

### Analysis of glycolytic rates.

The Seahorse XF Glycolytic Rate Assay Kit (Agilent) was used for measuring the ECAR and OCR. A total of 8 × 10^3^ cells per well were seeded in 96-well cell culture plates in RPMI 1640 medium supplemented with 10% FBS and incubated at 37°C overnight in a 5% CO_2_ incubator. The next day, the growth medium was replaced with bicarbonate-free RPMI 1640 medium, and the cells were incubated at 37°C for 1 hour in a CO_2_-free incubator. Analysis of ECAR and OCR was performed using the Extracellular Flux Analyzer XF96 (Seahorse Bioscience) under baseline conditions and treatment with (i) 0.5 μM rotenone and antimycin A or (ii) 50 mM 2-DG.

### Measurement of ATP levels.

Cellular ATP levels were determined using the Luminescent ATP Detection Assay kit (ab113849, Abcam) according to the manufacturer’s instructions. Luminescence intensity was detected using FLUOstar Omega plate reader (BMG LABTECH).

### Measurement of cellular ROS levels.

Assays of cellular ROS levels were performed using the DCFDA/H2DCFDA Cellular ROS Assay Kit, (ab113851, Abcam) according to the manufacturer’s instructions. ROS levels were measured using the FLUOstar Omega plate reader.

### Colony formation assays.

Cells were seeded in 24-well plates for 24 hours and then treated with ENZ, DTX, GO-203, DOX, or control vehicle every 3 days. After 7–10 days, the cells were stained with 0.5% crystal violet (LabChem) in 25% methanol. Colonies >25 cells were counted in triplicate wells as described (30, 51).

### Tumorsphere formation assays.

Cells (5 × 10^3^) were seeded per well in 6-well ultralow-attachment culture plates (Corning Life Sciences) in DMEM/F12 50/50 medium (Corning Life Sciences) with 20 ng/mL EGF (MilliporeSigma), 20 ng/mL bFGF (Millipore Sigma), and 1% B27 supplement (Gibco) as described (30, 51). Tumorspheres were counted under an inverted microscope (Eclipse TE2000-S, Nikon) in triplicate wells.

### In vivo experiments.

Six- to 8-week-old castrated male nude mice (The Jackson Laboratory) were injected subcutaneously in the flank with 5 × 10^6^ DU-145-DR cells in 100 μL of a 1:1 solution of medium and Matrigel (BD Biosciences). When the mean tumor volume reached 100–200 mm^3^, mice were pair-matched into groups of 6 mice each. Mice were treated intraperitoneally with PBS as the vehicle control, DTX (15 mg/kg) alone weekly, GO-203 (15 mg/kg) alone daily, and DTX in combination with GO-203. Tumor tissues were prepared for IHC staining as described below for patient samples. Slides were incubated with anti–MUC1-C (16564s, 1:200, Thermo Fisher Scientific) and anti-LDHA (3582s, 1:200 dilution; CST) for 8 hours at 4°C and counterstained with hematoxylin. For M1C ADC studies, castrated 6- to 8-week-old NOD/SCID-γ male mice (NOD.Cg-PrkdcscidIl2rgtm1Wjl/SzJ; The Jackson Laboratory) were injected subcutaneously in the flank with (i) 5 × 10^6^ H660 cells in 100 μL of a 1:1 solution of medium and Matrigel (BD Biosciences) or (ii) transplanted WCM154 PDX tumor. The M1C ADC (5 or 7.5 mg/kg) provided by the NCI NExT Program or PBS as the control vehicle was injected intravenously in the tail vein once each week for 3 weeks every 28 days.

Protocol-approved experimental and humane endpoints were followed using criteria of tumor size reaching 2 cm in any dimension and 15% weight loss from last maximum weight measurement.

### ScRNA-Seq studies.

ScRNA-Seq data of PC patient biopsies were obtained from Zaidi et al. (38) National Center for Biotechnology Information (NCBI) Gene Expression Omnibus (GEO) GSE264573 and GSE210358 and reanalyzed according to the authors’ methods using Seurat (54). Tumor subtype of CRPC and NEPC was based on the clinical characteristics described by Zaidi et al. (38). Further division of tumors into CRPC/AR^+^ and CRPC/AR^–^ subtypes was based on mean AR expression levels. Log-normalized gene expression counts were used for all analyses and visualization, unless otherwise stated. To calculate GSEAs for each cell, AUCell (55) was implemented using the following curated and previously published gene sets: Hallmark Glycolysis (MSigDB) (56), NEPC score obtained from Beltran et al. (57), and AR score obtained from Chan et al. (58). For Pearson’s correlations, gene expression counts for MUC1 were first imputed using MAGIC (59).

### Statistics.

Data are expressed as the mean ± SD. The unpaired 2-tailed *t* test was used to determine differences between means of groups. A *P* value of less than 0.05 denoted by an asterisk (*) was considered statistically significant with confidence intervals equal to 95%.

### Study approval.

All animal studies were approved by the Dana-Farber Cancer Institute IACUC under Protocol #03-029.

### Data availability.

The accession numbers for the RNA-Seq data are GEO GSE276750, GSE276890, and GSE289308. ScRNA-Seq data of PC patient biopsies was obtained from Zaidi et al. (38) GEO GSE264573 and GSE210358. Supporting data values associated with the main manuscript and supplement material are included in the Supporting Data Values file.

## Author contributions

Conceptualization was done by KS, TD, and DK. Methodology was developed by KS, TD, HH, SYK, MK, and MR. Investigation was done by KS, NH, HO, AN, AB, ST, and AF. Data curation was done by KS, TD, HH, SYK, YB, TK, HO, JJ, MDL, and MO. Software was developed by KS, MK, MR, and DK. KS and DK wrote the original draft. KS, TD, HH, SYK, NH, HO, AN, AB, MO, and DK reviewed and edited the draft. HB and DK supervised. HB and DK acquired funding.

## Supplementary Material

Supplemental data

Unedited blot and gel images

Supporting data values

## Version 1 06/24/2025

In-Press Preview

## Version 2 07/22/2025

Electronic publication
