# Supplementary material for: MUC1-C dependence in treatment-resistant prostate cancer uncovers a target for antibody-drug conjugate therapy
Source: JCI Insight. 2025 Jun 24;10(14):e190924. doi: 10.1172/jci.insight.190924 (PMC12288968; doi:10.1172/jci.insight.190924)
Supplement: Supplemental data [file jciinsight-10-190924-s080.pdf]

# Supplemental Figure 1

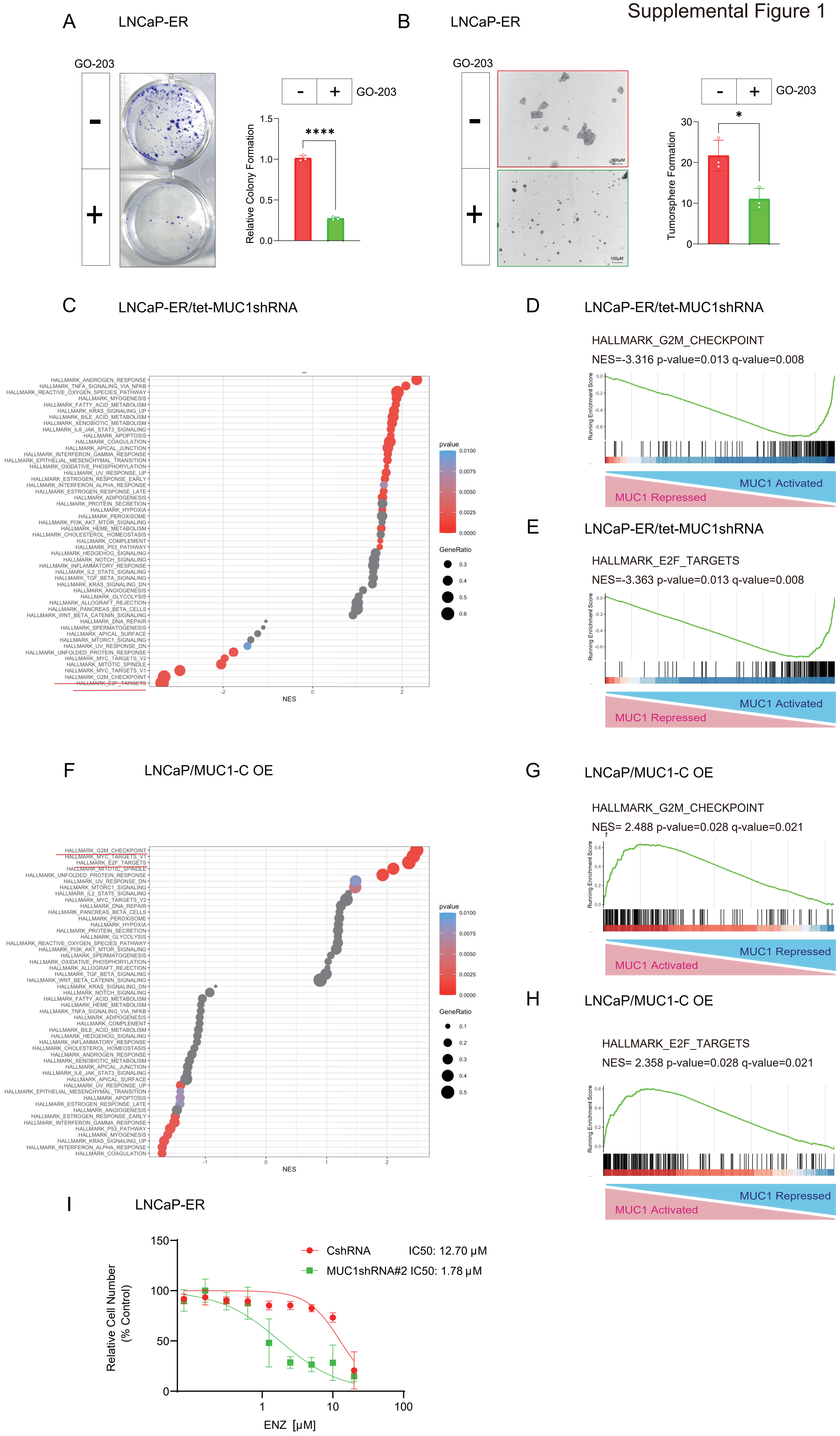

**Supplemental Figure S1. MUC1-C regulates the transcriptomes of LNCaP-ER and LNCaP/MUC1-C OE cells. A.** LNCaP-ER cells treated with vehicle or 3 μM GO-203 for 3 days were analyzed for colony formation. Shown are representative photomicrographs of stained colonies (left). The results (mean±SD of three determinations) are expressed as relative colony number compared to that for untreated cells (assigned a value of 1)(right)(t-test; n=3). **B.** LNCaP-ER cells treated with vehicle or 3 μM GO-203 for 3 days were analyzed for tumorsphere formation. Shown are representative photomicrographs of tumorspheres (left). The results (mean±SD of three determinations) are expressed as tumorsphere number (right) (t-test; n=3). **C.** RNA-seq was performed in triplicate on LNCaP-ER/tet-MUC1shRNA cells treated with vehicle or DOX for 7 days. The data was analyzed using the indicated HALLMARK gene signatures. **D and E.** GSEA of RNA-seq data from LNCaP-ER cells with MUC1-C silencing using the HALLMARK G2M CHECKPOINT (**D**) and HALLMARK E2F TARGETS (**E**) gene signatures. **F.** RNA-seq was performed in triplicate on LNCaP and LNCaP/MUC1-C OE cells. The data was analyzed using the indicated HALLMARK gene signatures. **G and H.** GSEA of RNA-seq data from LNCaP and LNCaP/MUC1-C OE cells using the HALLMARK G2M CHECKPOINT (**G**) and HALLMARK E2F TARGETS (**H**) gene signatures. **I.** LNCaP-

ER/CshRNA and LNCaP-ER/MUC1shRNA#2 treated with the indicated concentrations of ENZ for 3 days were analyzed for cell viability by Alamar Blue staining. The results (mean±SD of six determinations) are expressed as relative cell number (% control) compared to that for untreated cells. Indicated are the ENZ IC50 values.

# Supplemental Figure 2

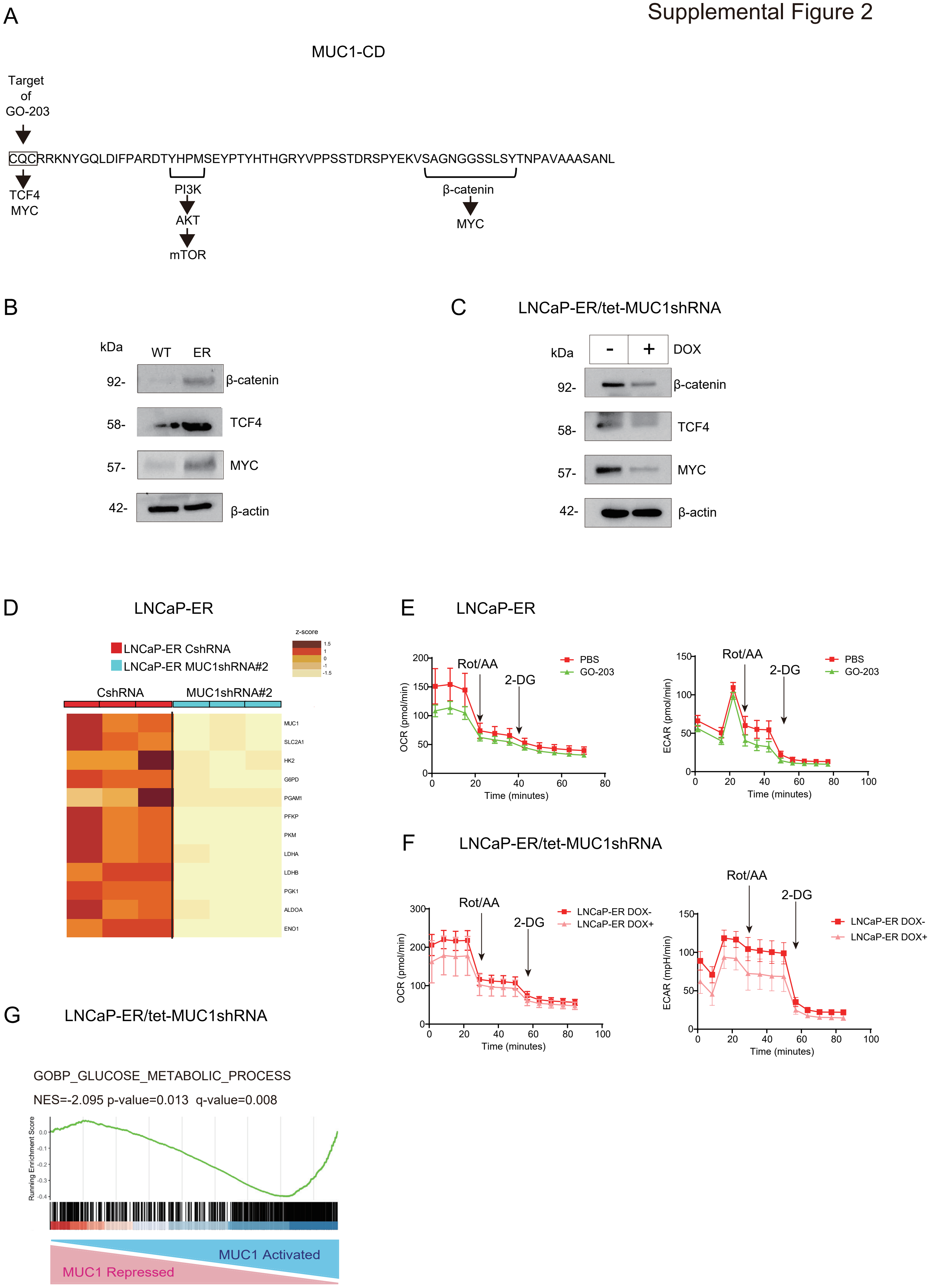

**Supplemental Figure S2. MUC1-C regulates MYC expression and associates with the glucose metabolic process. A.** Amino acid sequence of the MUC1-C cytoplasmic domain (MUC1-CD) highlighting (i) direct binding of the CQC motif with MYC and TCF4 and (ii) interaction of the serine-rich motif (SRM) with β-catenin. MUC1-CD facilitates the formation of TCF4/β-catenin complexes in activating the *MYC* gene. MUC1-CD also includes a YHPM sequence that, when phosphorylated on tyrosine, conforms to a consensus sequence for binding of the PI3K SH2 domain. **B.** Immunoblot analysis of lysates from LNCaP and LNCaP-ER cells run contemporaneously in parallel. **C.** Immunoblot analysis of lysates from LNCaP-ER/tet-MUC1shRNA cells treated with vehicle or DOX for 7 days run contemporaneously in parallel. **D.** Heatmap of glycolysis gene expression in LNCaP-ER/CshRNA and LNCaP-ER/MUC1shRNA#2 cells. **E and F.** LNCaP-ER cells treated with vehicle or 3 μM GO-203 (**E**) and LNCaP-ER/tet-MUC1shRNA cells treated with vehicle or DOX for 7 days (**F**) were assayed for oxygen consumption rate (OCR) and extracellular acid rate (ECAR). Rotenone and Antimycin A (ROT/AA) and 2-deoxyglucose (2-DG) were added at the indicated times. The OCR and ECAR results (mean±SD of four determinations) are expressed as pmol/min. **G.** GSEA of RNA-seq data from LNCaP-ER cells with MUC1-C silencing using the GOBP GLUCOSE METABOLIC PROCESS gene signature.

# Supplemental Figure 3

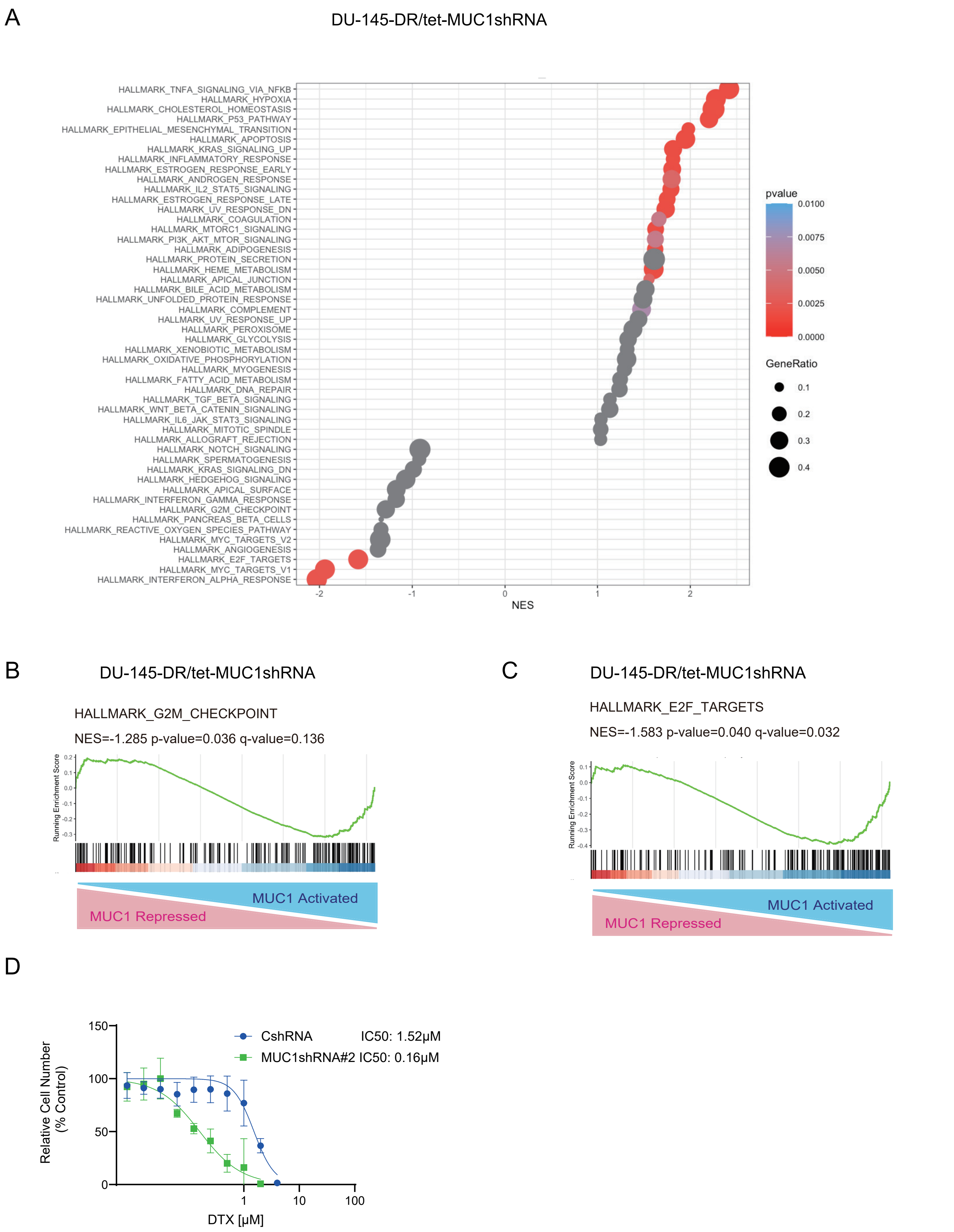

**Supplemental Figure S3. Silencing MUC1-C in DOX-treated DU-145-DR/tet-MUC1shRNA cells regulates gene transcriptomes. A.** Analysis of RNA-seq data from DU-145-DR/tet-MUC1shRNA treated with vehicle or DOX for 7 days using the indicated HALLMARK gene signatures. **B and C.** GSEA of RNA-seq data from DU-145-DR cells with MUC1-C silencing using the HALLMARK G2M CHECKPOINT (**B**) and HALLMARK E2F TARGETS (**C**) gene signatures. **D.** DU-145-DR/CshRNA and DU-145-DR/MUC1shRNA#2 treated with the indicated concentrations of DTX for 3 days were analyzed for cell viability by Alamar Blue staining. The results (mean±SD of six determinations) are expressed as relative cell number (% control) compared to that for untreated cells. Indicated are the DTX IC50 values.

# Supplemental Figure 4

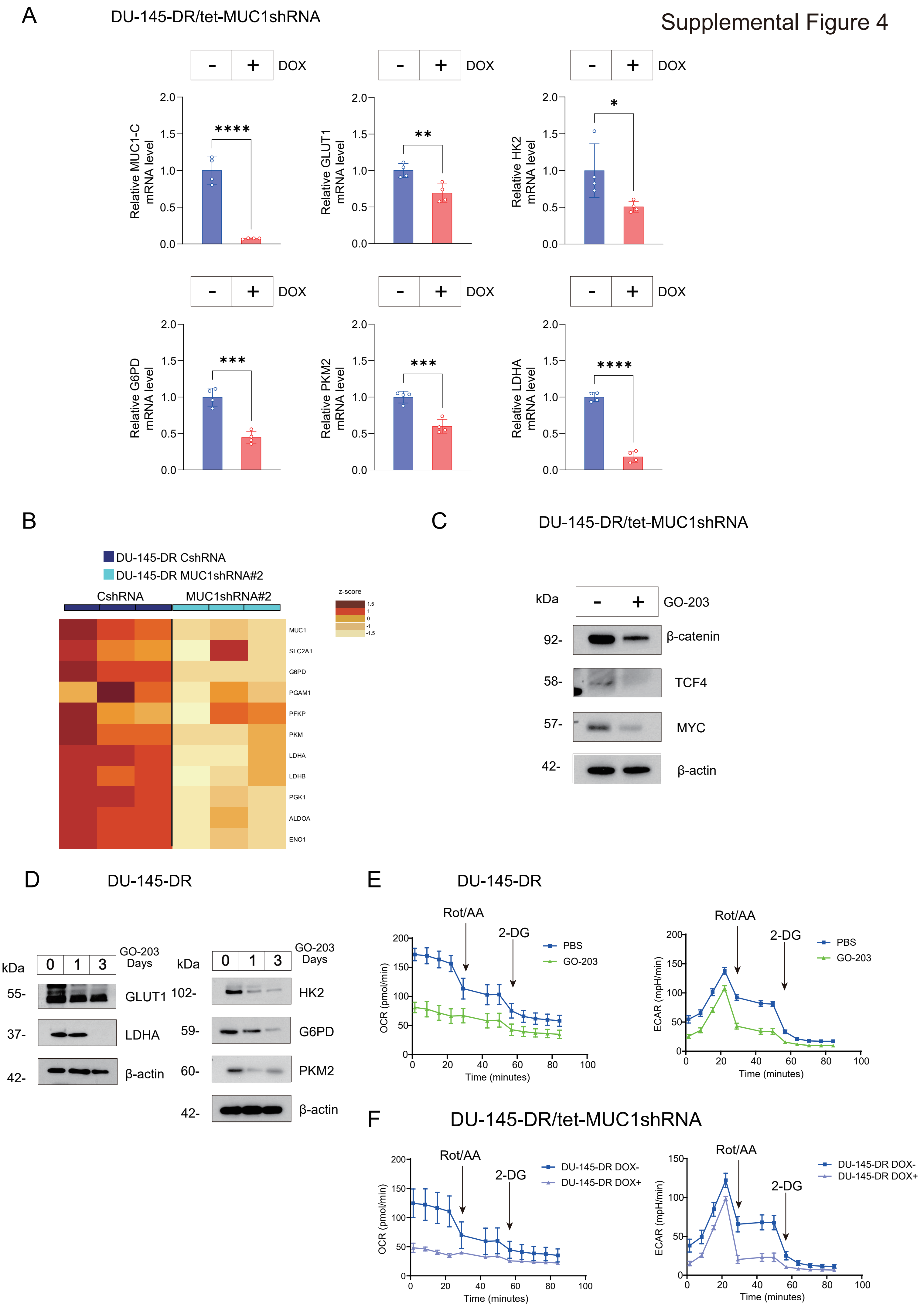

**Supplemental Figure S4. Effects of targeting MUC1-C on gene expression in DU-145-DR cells. A.** DU-145-DR/tet-MUC1shRNA cells treated with vehicle or DOX for 7 days were analyzed for the indicated transcripts by qRT-PCR. The results (mean±SD of four determinations) are expressed as relative levels compared to that obtained for vehicle-treated cells (assigned a value of 1) (t-test; n=3). **B.** Heatmap of glycolysis gene expression in DU-145-DR/CshRNA and DU-145-DR/MUC1shRNA#2 cells. **C.** Immunoblot analysis of lysates from DU-145-DR cells treated with vehicle or 3 μM GO-203 for 3 days run contemporaneously in parallel. **D.** Immunoblot analysis of lysates from DU-145-DR cells treated with vehicle or 3 μM GO-203 for 1 and 3 days run at different times. **E and F.** DU-145-DR cells treated with vehicle or 3 μM GO-203 (**E**) and DU-145-DR/tet-MUC1shRNA cells treated with vehicle or DOX for 7 days (**F**) were assayed for OCR and ECAR. ROT/AA and 2-DG were added at the indicated times. The OCR and ECAR results (mean±SD of four determinations) are expressed as pmol/min.

Supplemental Figure 5

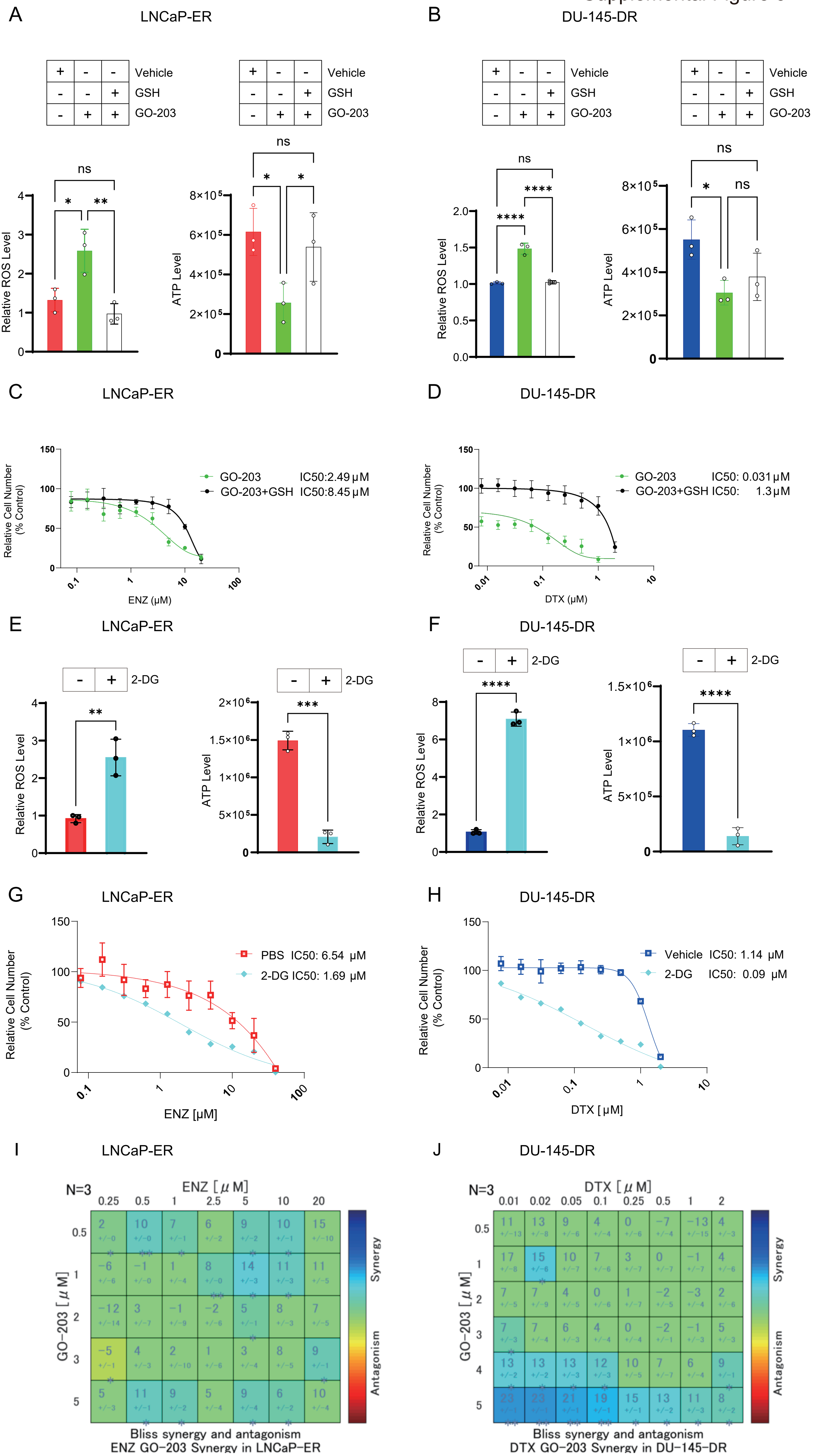

**Supplemental Figure S5. Targeting MUC1-C with GO-203 regulates ROS and ATP levels in LNCaP-ER and DU-145-DR cells. A and B.** LNCaP-ER (**A**) and DU-145-DR (**B**) cells treated with vehicle, 3 µM GO-203, 3 µM GO-203 and 2.5 µM GSH for 3 days were analyzed for ROS and ATP levels. The results (mean±SD of three determinations) are expressed as (i) relative ROS levels compared to that for vehicle-treated cells (assigned a value of 1) and (ii) absolute ATP levels as determined by luminescence without and with GO-203 treatment. (t-test; n=3). **C.** LNCaP-ER cells treated with 3 µM GO-203 and 3 µM GO-203+2.5 µM GSH and then with the indicated concentrations of ENZ for 3 days were analyzed for cell viability. **D.** DU-145-DR cells treated with 3 µM GO-203 and 3 µM GO-203+2.5 µM GSH and then with DTX for 3 days were analyzed for cell viability. **E and F.** LNCaP-ER (**E**) and DU-145-DR (**F**) cells treated with vehicle or 10 mM 2-DG for 3 days were analyzed for ROS and ATP levels. The results (mean±SD of three determinations) are expressed as (i) relative ROS levels compared to that for vehicle-treated cells (assigned a value of 1) and (ii) absolute ATP levels as determined by luminescence without and with 2-DG treatment(t-test; n=3). **G.** LNCaP-ER cells treated with vehicle or 10 mM 2-DG and ENZ for 3 days were analyzed for cell viability. **H.** DU-145-DR cells treated with vehicle or 10 mM 2-DG and DTX for 3 days were analyzed for cell viability. **I.** Combination index values for LNCaP-ER cells treated with ENZ and GO-203 as determined using Bliss scores (0=additive, >0: synergistic, <0: antagonistic). **J.** Combination index values for DU-145-DR cells treated with DTX and GO-203 as determined using Bliss scores (0=additive, 0>: synergistic, <0: antagonistic).

# Supplemental Figure 6

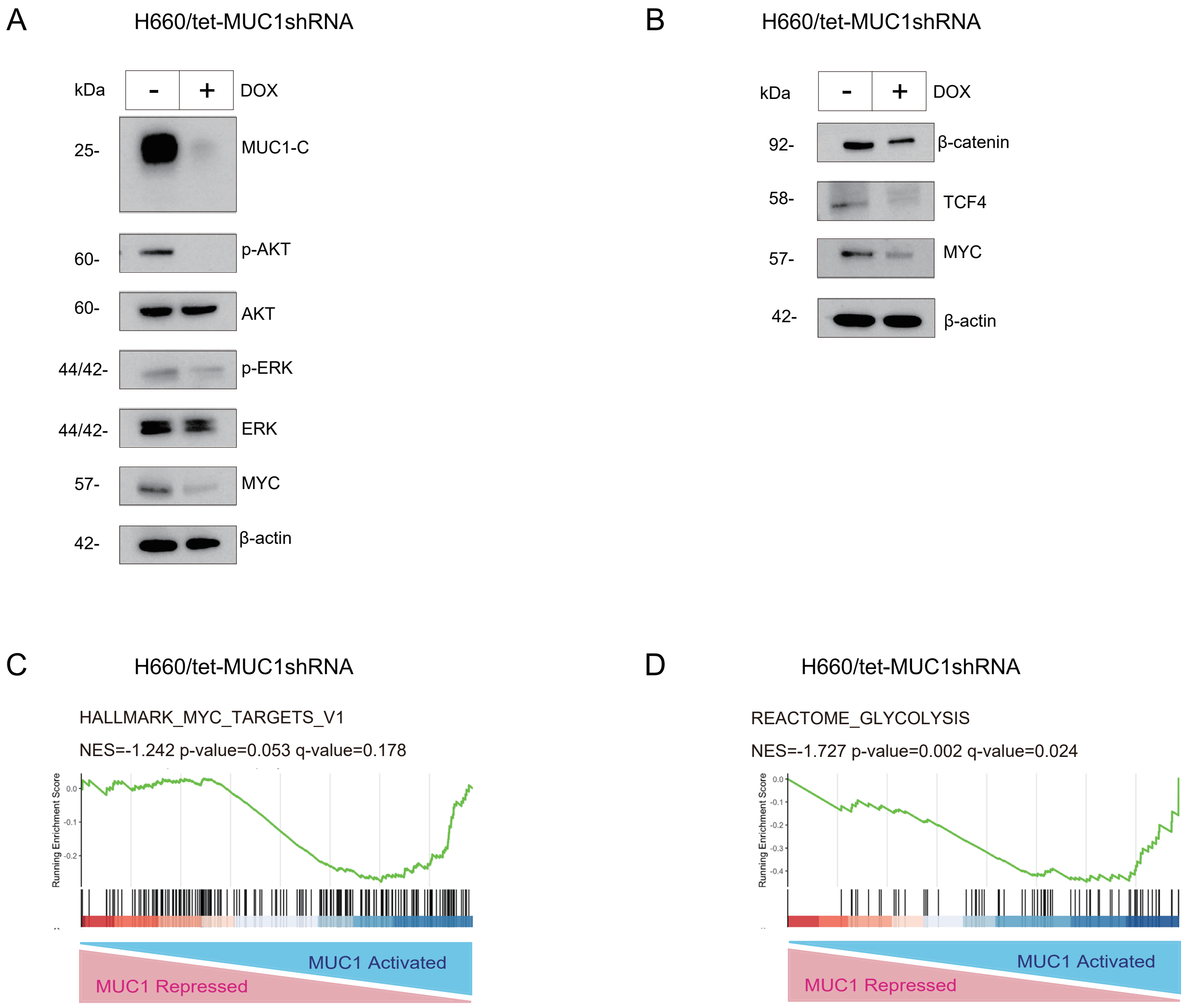

**Supplemental Figure S6. Effects of targeting MUC1-C in H660 t-NEPC cells. A.** Immunoblot analysis of lysates from H660/tet-MUC1shRNA cells treated with vehicle or DOX for 7 days run contemporaneously in parallel. **B.** Immunoblot analysis of lysates from H660/tet-MUC1shRNA cells treated with vehicle or DOX run contemporaneously in parallel. **C and D.** GSEA of RNA-seq data from H660 cells with MUC1-C silencing using the HALLMARK MYC TARGETS V1 (**C**) and REACTOME GLYCOLYSIS (**D**) gene signatures.

Supplemental Figure 7

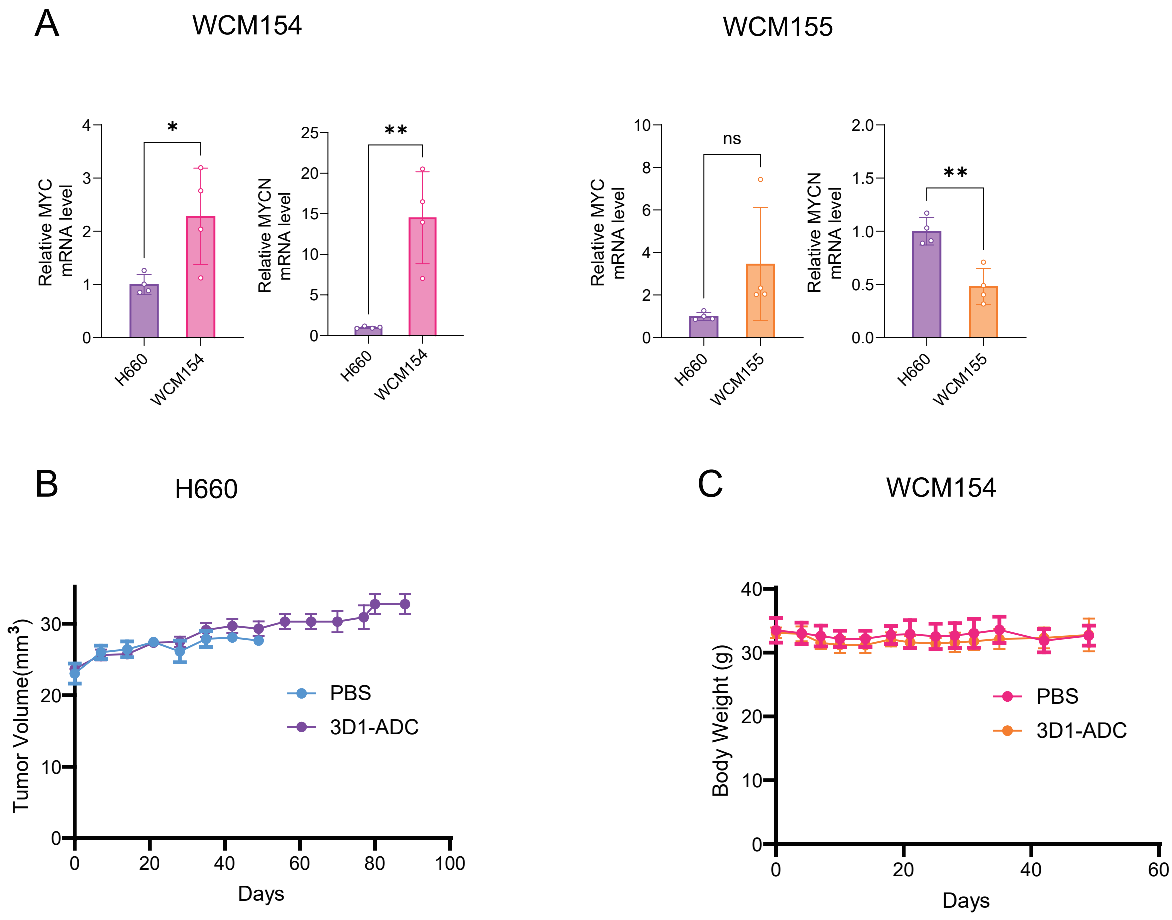

**Supplemental Figure S7. Effects of targeting MUC1-C in t-NEPC cells and scRNA-seq data of treatment resistant CRPC and NEPC models. A.** H660 and patient-derived WCM154 and WCM155 t-NEPC cells were analyzed for the indicated transcripts by qRT-PCR. The results (mean±SD of four determinations) are expressed as relative levels compared to that obtained for DU-145-DR cells (assigned a value of 1)(t-test; n=3). **B.** Body weights expressed as the mean±SD for 6 mice bearing H660 tumor xenografts treated with PBS control vehicle or M1C ADC. **C.** Body weights expressed as the mean±SD for 6 mice bearing WCM154 PDX xenografts treated with PBS control vehicle or M1C ADC.
