# Supplementary material for: MUC1-C dependence in treatment-resistant prostate cancer uncovers a target for antibody-drug conjugate therapy
Source: JCI Insight. 2025 Jun 24;10(14):e190924. doi: 10.1172/jci.insight.190924 (PMC12288968; doi:10.1172/jci.insight.190924)

Unedited Gels and Blots

Figure 1B

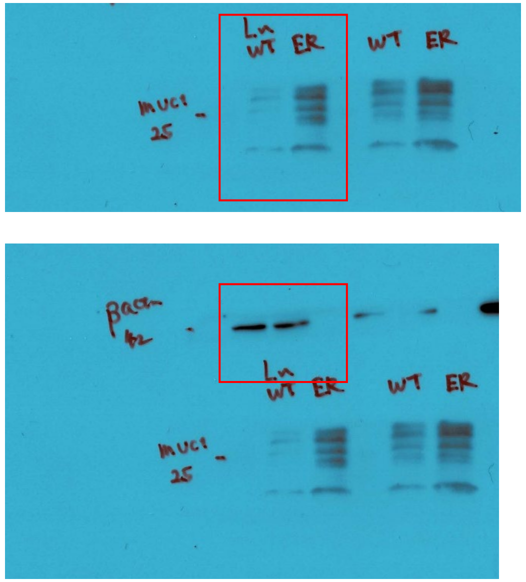

MUC1-C
25kDa

β-actin
42kDa

Figure 1D

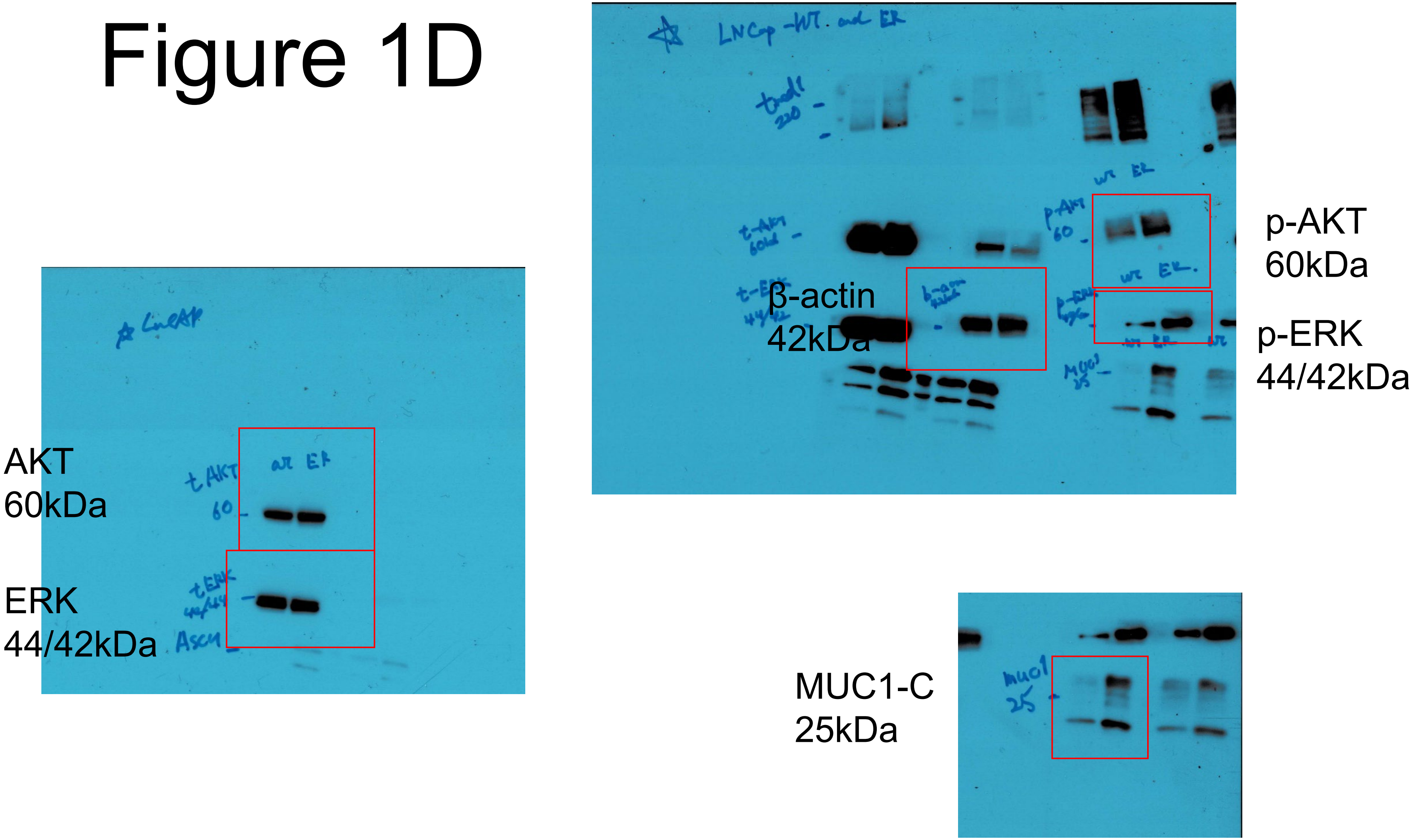
p-AKT
60kDa
β-actin
42kDa
p-ERK
44/42kDa
AKT
60kDa
ERK
44/42kDa
MUC1-C
25kDa

Figure 1G

MUC1-C
25kDa

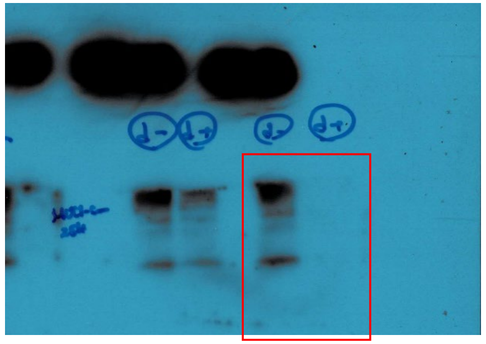

β-actin
42kDa

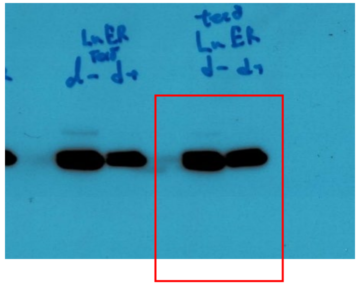

AKT
60kDa

ERK
44/42kDa

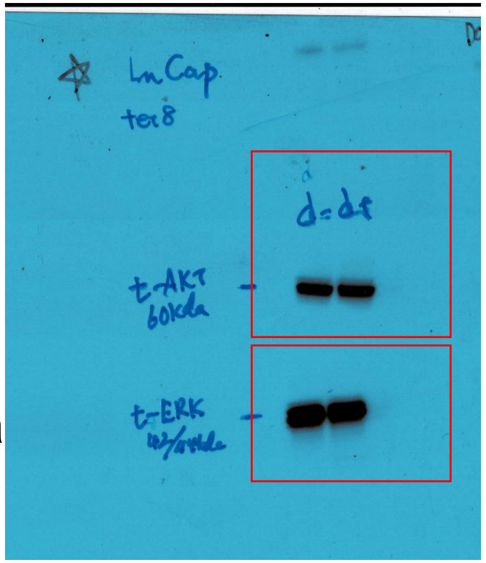

p-AKT
60kDa

p-ERK
44/42kDa

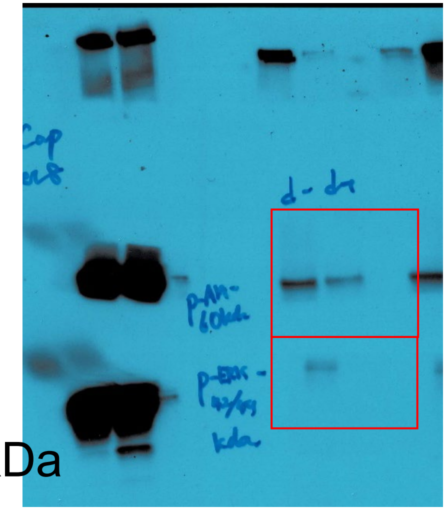

Figure 1J

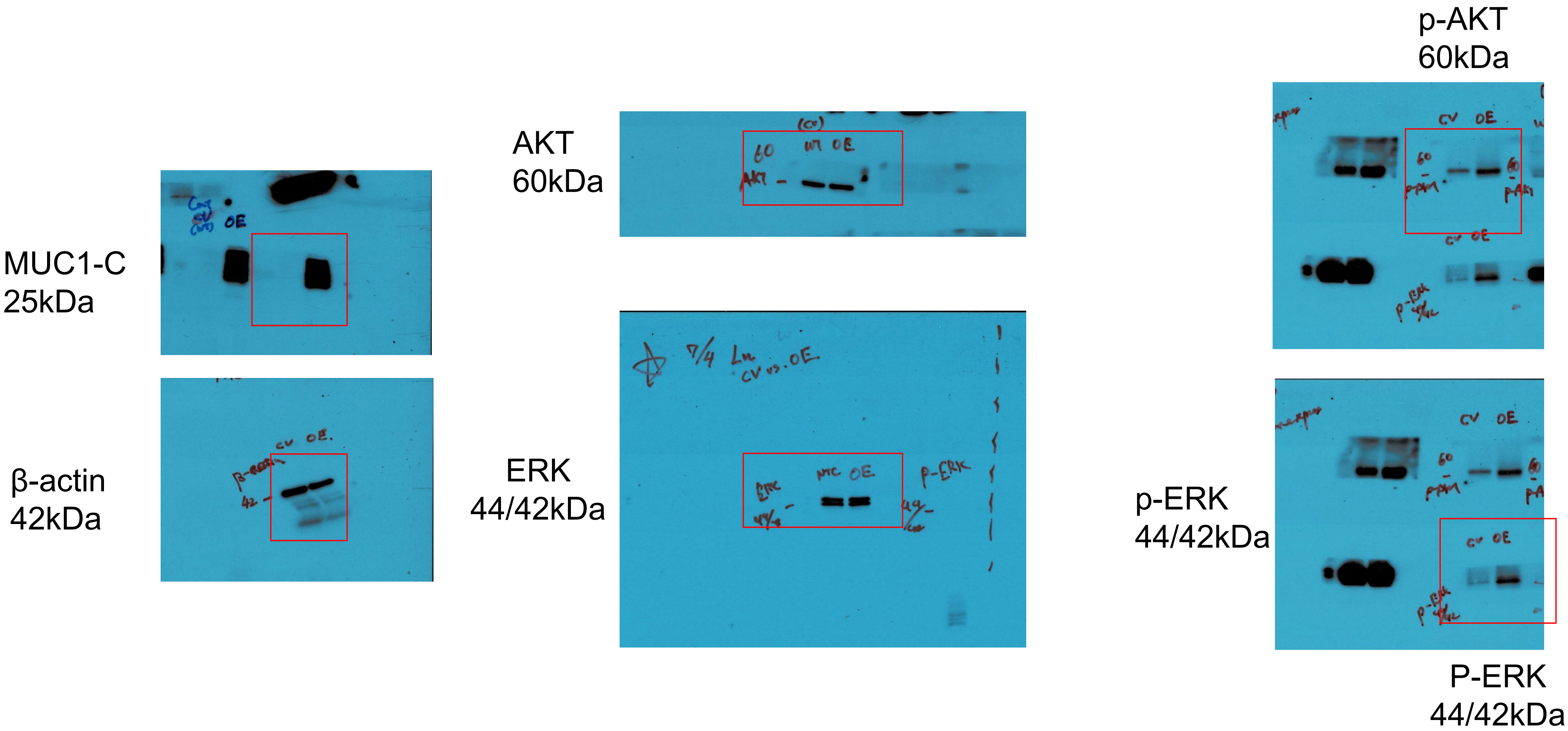

MUC1-C
25kDa
β-actin
42kDa
AKT
60kDa
ERK
44/42kDa
p-AKT
60kDa
p-ERK
44/42kDa
P-ERK
44/42kDa

Figure 2A

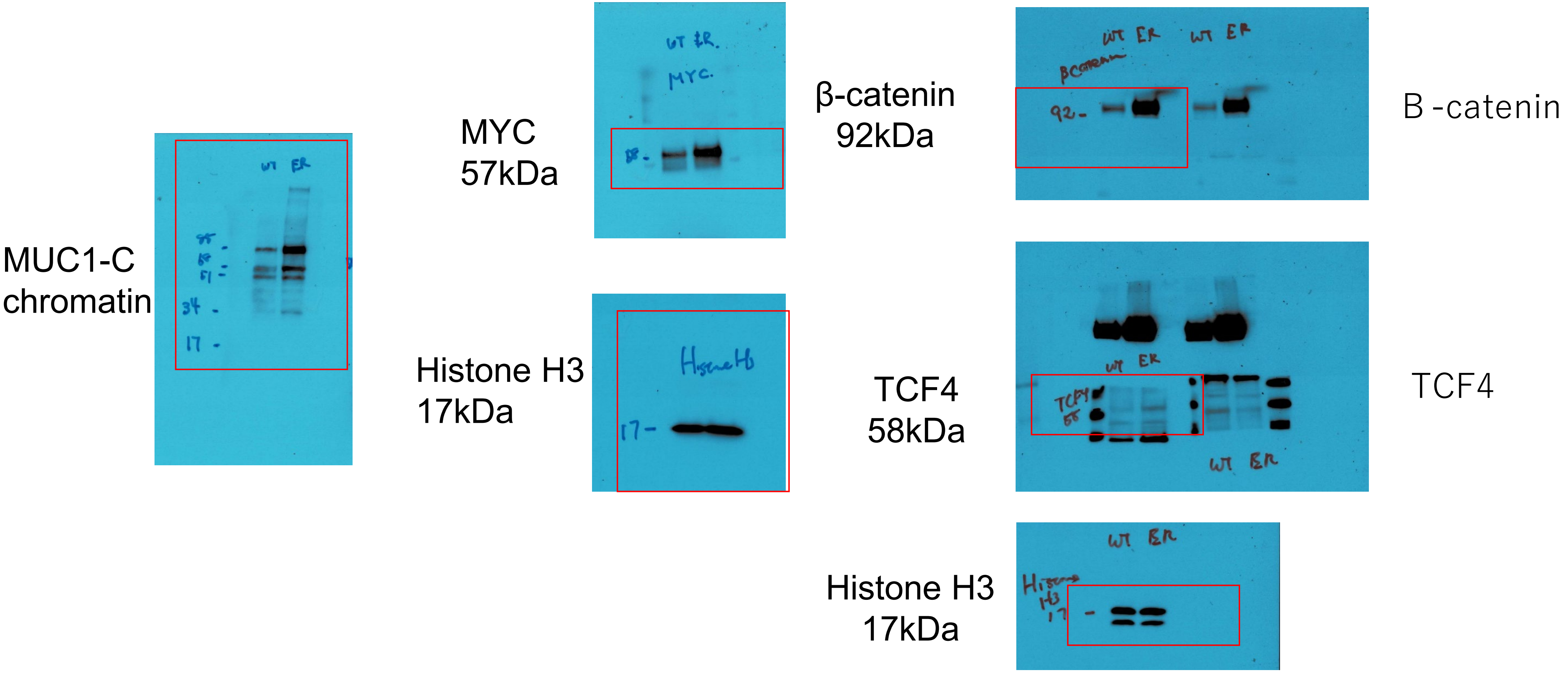
MUC1-C
chromatin
MYC
57kDa
Histone H3
17kDa
β-catenin
92kDa
B -catenin
TCF4
58kDa
TCF4
Histone H3
17kDa

Figure 2B

MUC1-C
chromatin

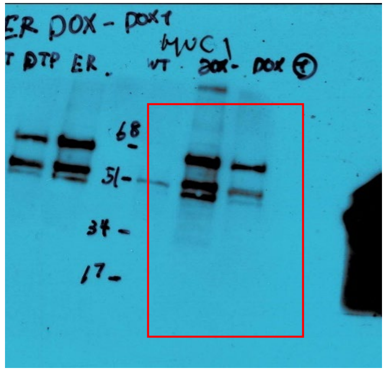

β-catenin
92kDa

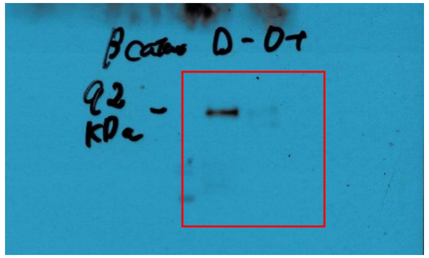

TCF4
58kDa

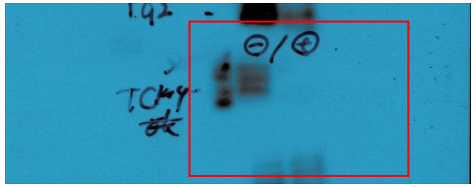

Histone H3
17kDa

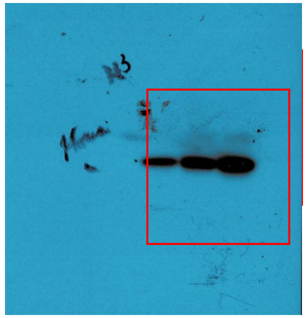

MYC
57kDa

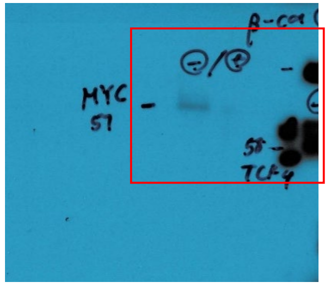

Histone H3
17kDa

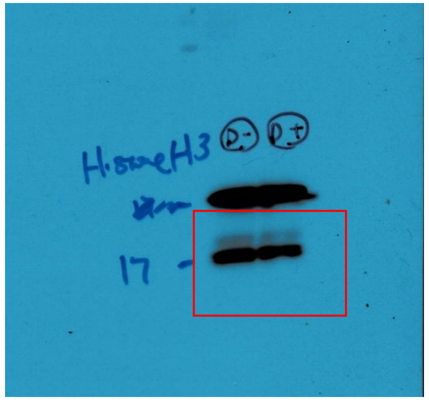

Figure 2C

β-catenin
92kDa

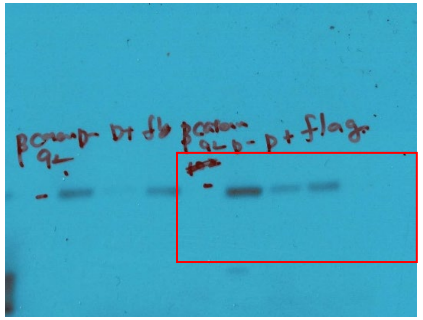

MYC
57kDa

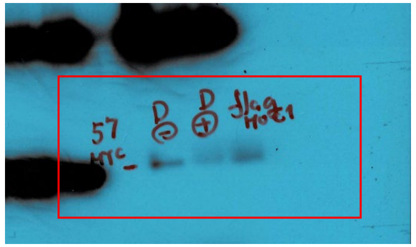

TCF4
58kDa

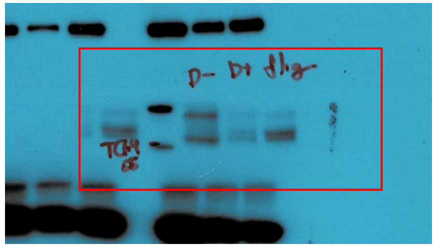

MUC1-C
25kDa

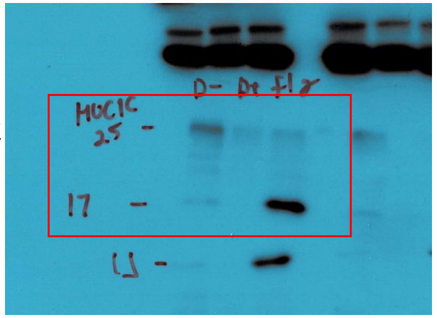

β-actin
42kDa

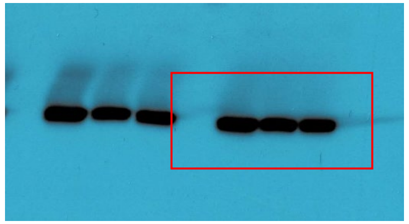

Figure 2F

GLUT1
55kDa

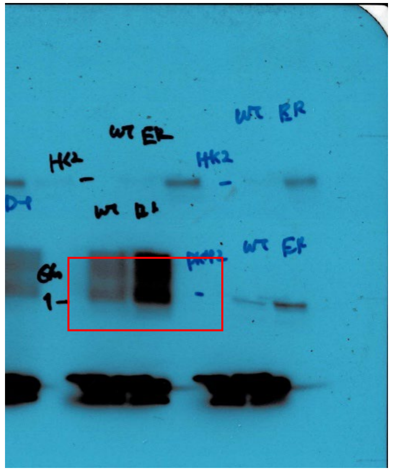

β-actin
42kDa

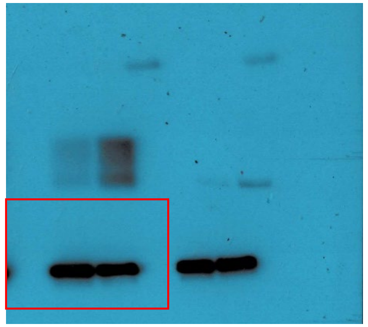

LDHA
37kDa

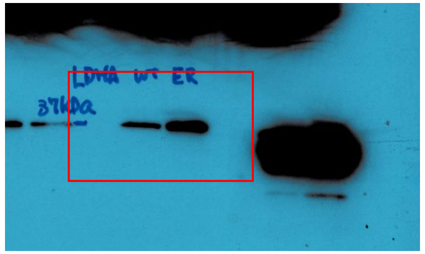

HK2
102kDa

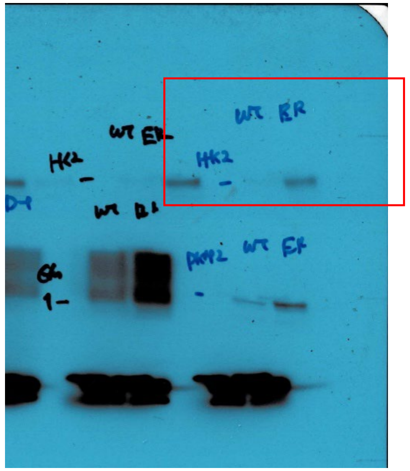

PKM2
60kDa

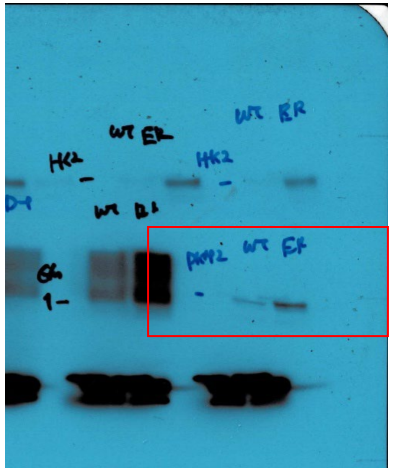

G6PD
59kDa

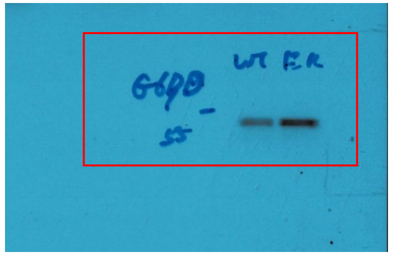

Figure 2H

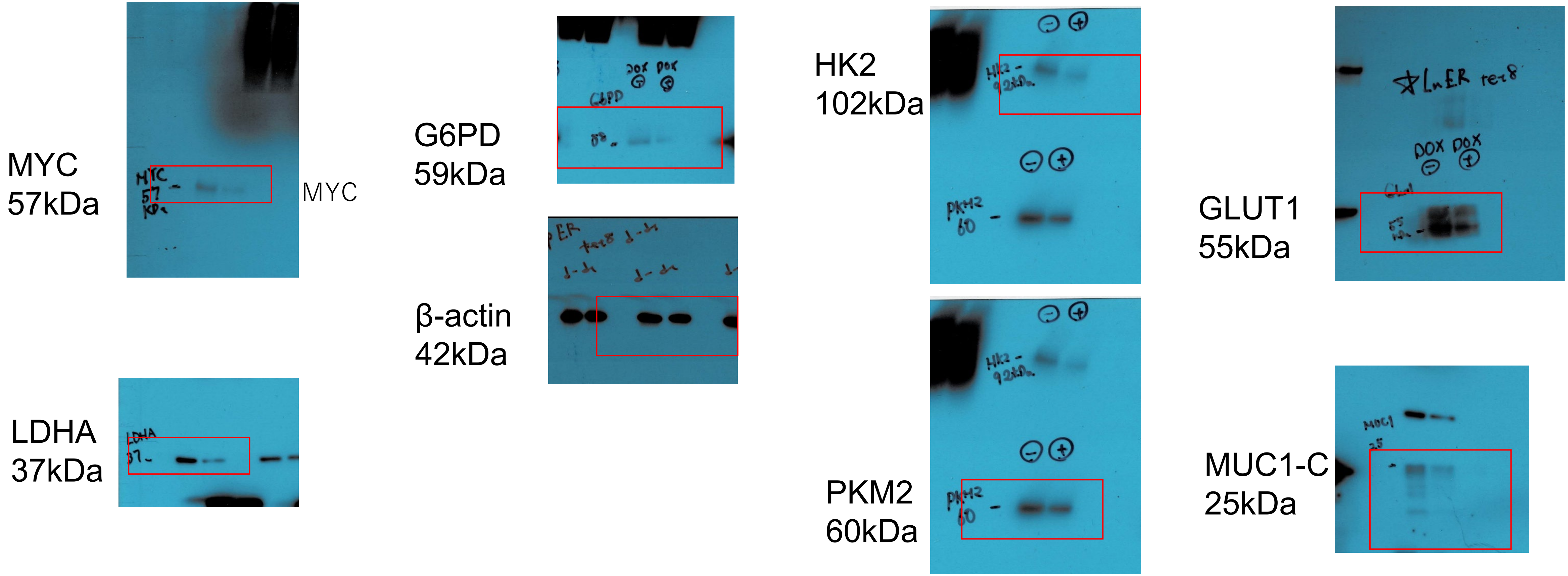
MYC
57kDa
MYC
G6PD
59kDa
β-actin
42kDa
HK2
102kDa
GLUT1
55kDa
LDHA
37kDa
PKM2
60kDa
MUC1-C
25kDa

Figure 2K

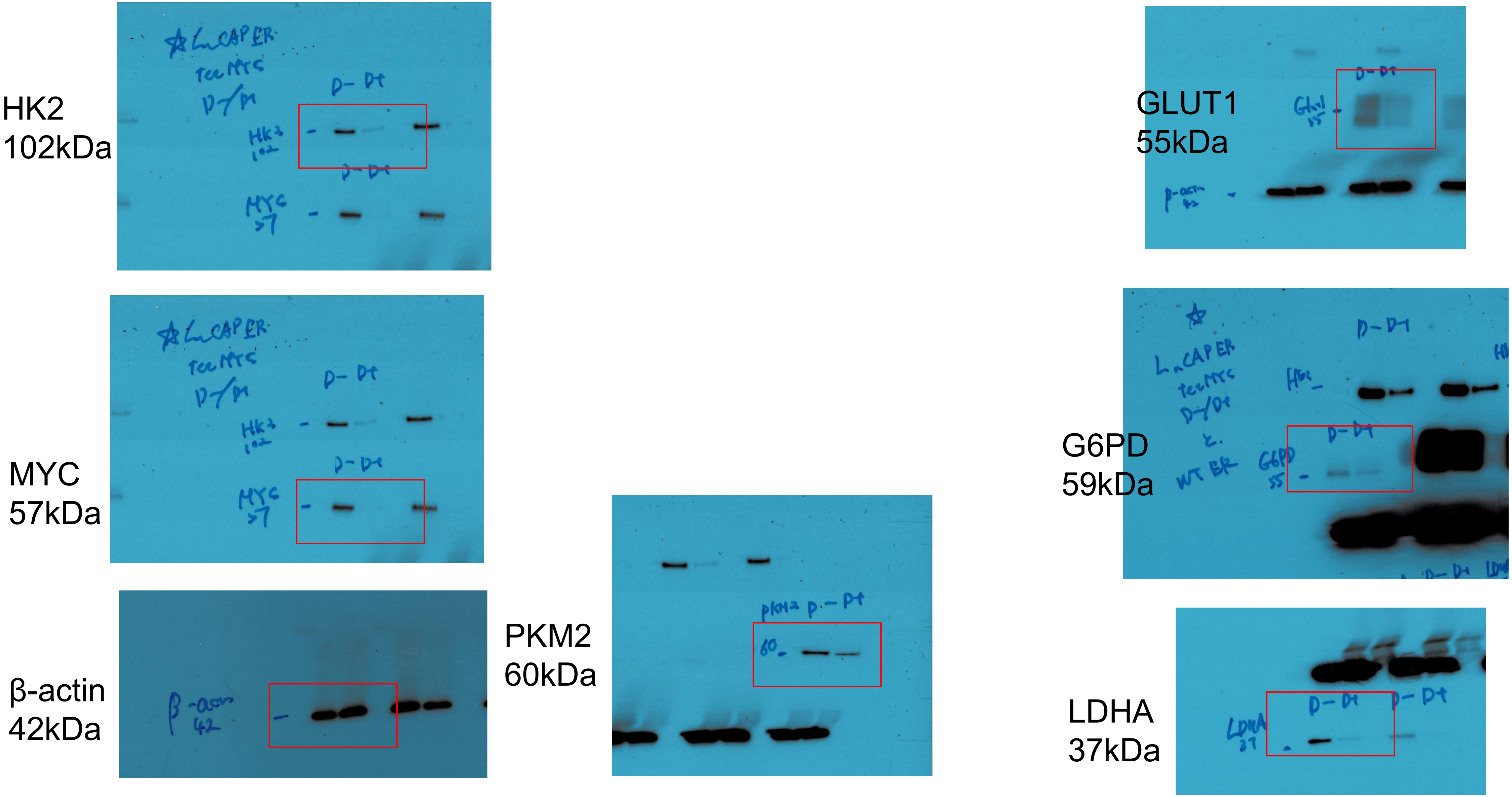
HK2
102kDa
MYC
57kDa
β-actin
42kDa
PKM2
60kDa
GLUT1
55kDa
G6PD
59kDa
LDHA
37kDa

Fig S2B

β-catenin
92kDa

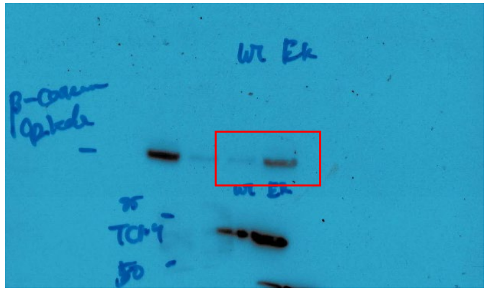

TCF4
58kDa

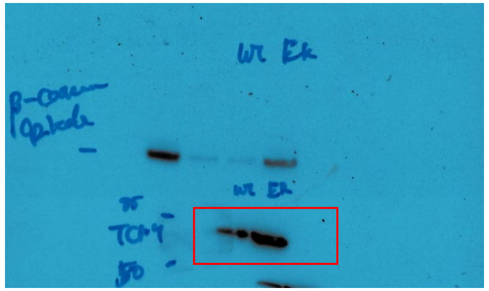

MYC
57kDa

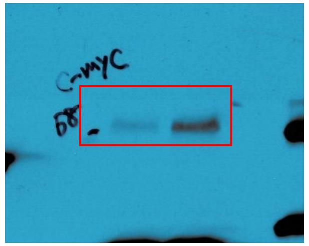

β-actin
42kDa

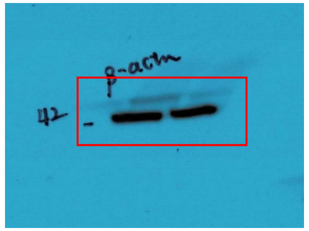

Figure S2C

TCF4
58kDa

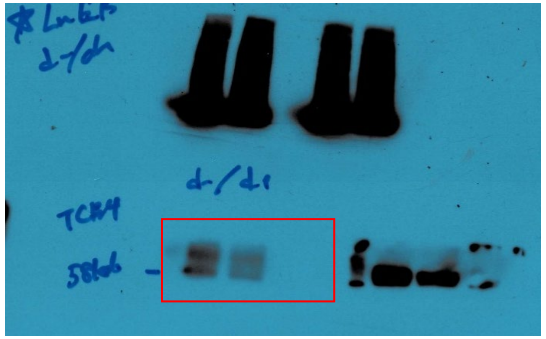

MYC
57kDa

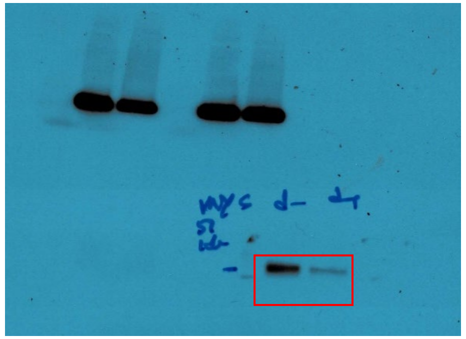

β-catenin
92kDa

β-actin
42kDa

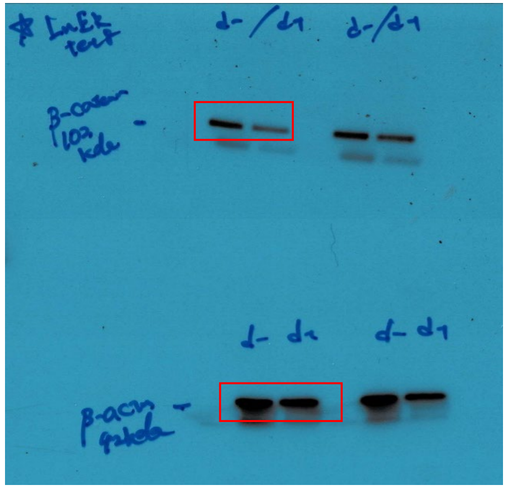

Figure 3C

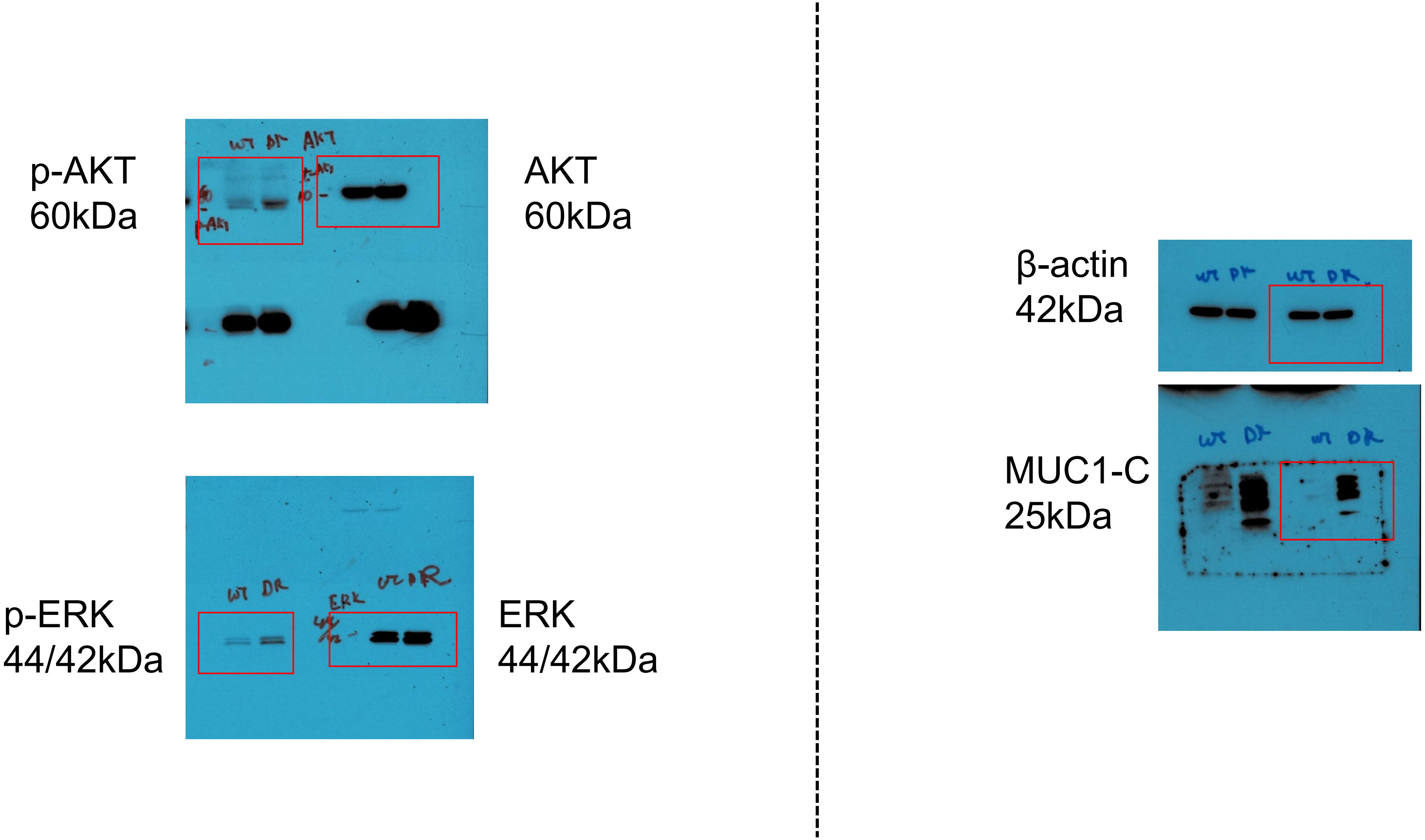
p-AKT
60kDa
AKT
60kDa
p-ERK
44/42kDa
ERK
44/42kDa
β-actin
42kDa
MUC1-C
25kDa

Figure 3F

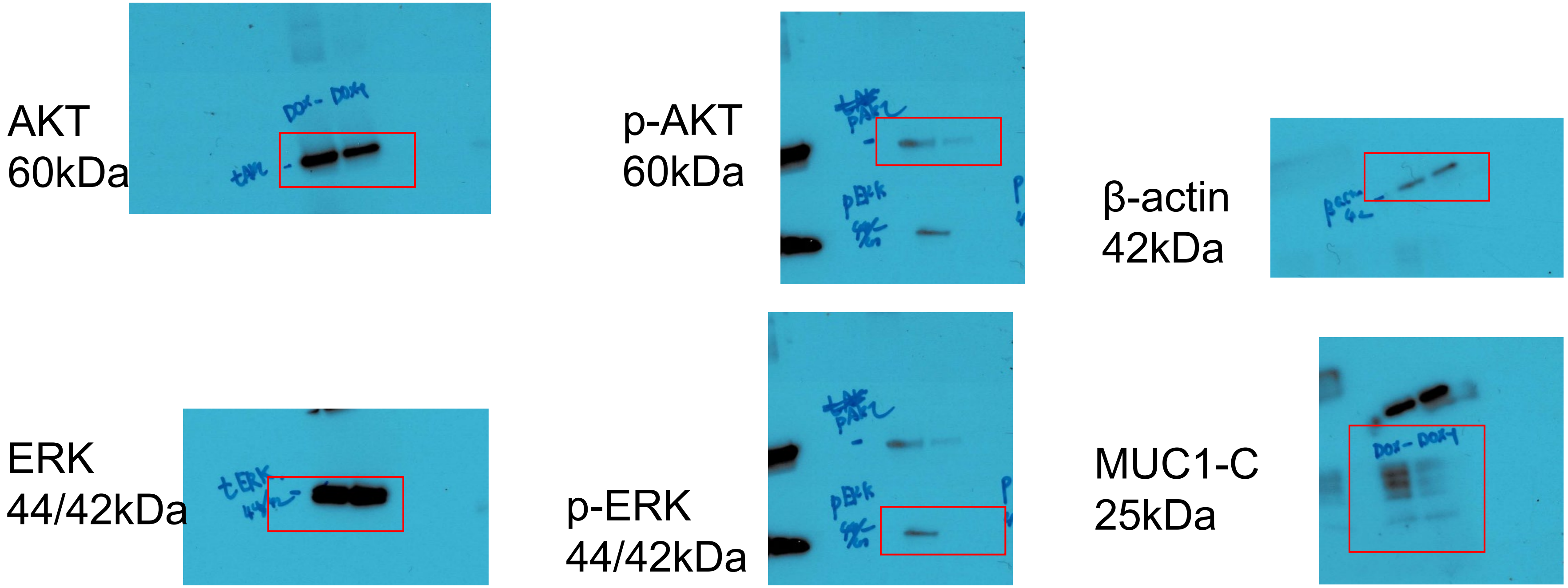
AKT
60kDa
p-AKT
60kDa
β-actin
42kDa
ERK
44/42kDa
p-ERK
44/42kDa
MUC1-C
25kDa

Figure 3I

β-actin
42kDa

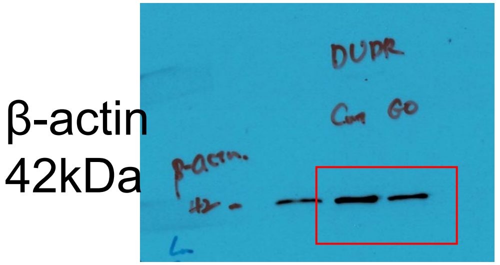

AKT
60kDa

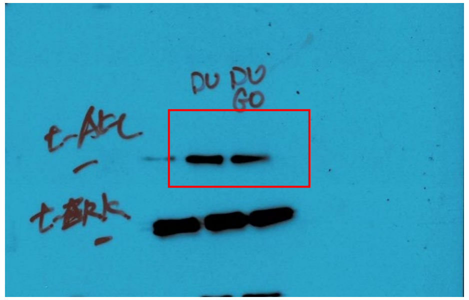

ERK
44/42kDa

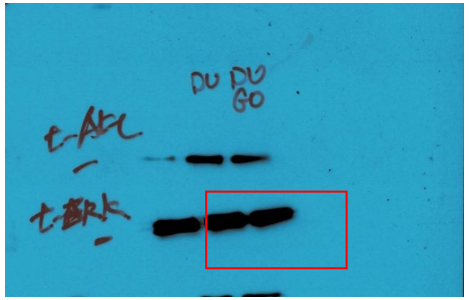

p-AKT
60kDa

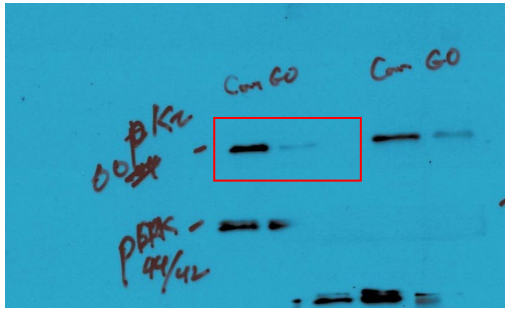

p-ERK
44/42kDa

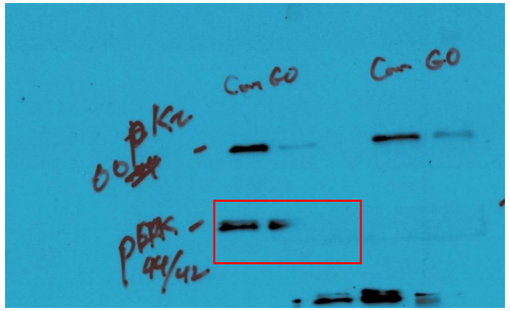

Figure 4A

β-catenin
92kDa

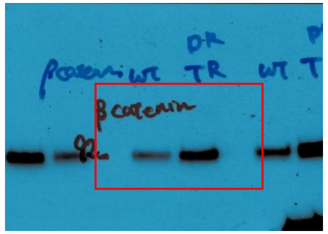

TCF4
58kDa

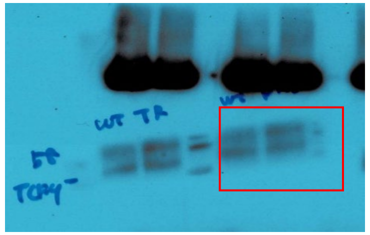

MYC
57kDa

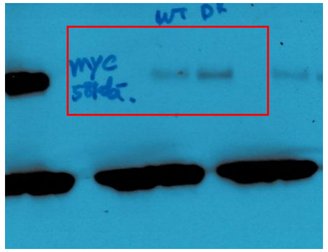

β-actin
42kDa

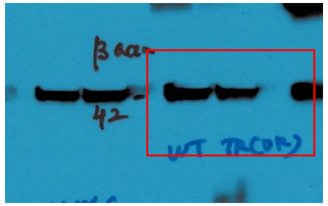

Figure 4B

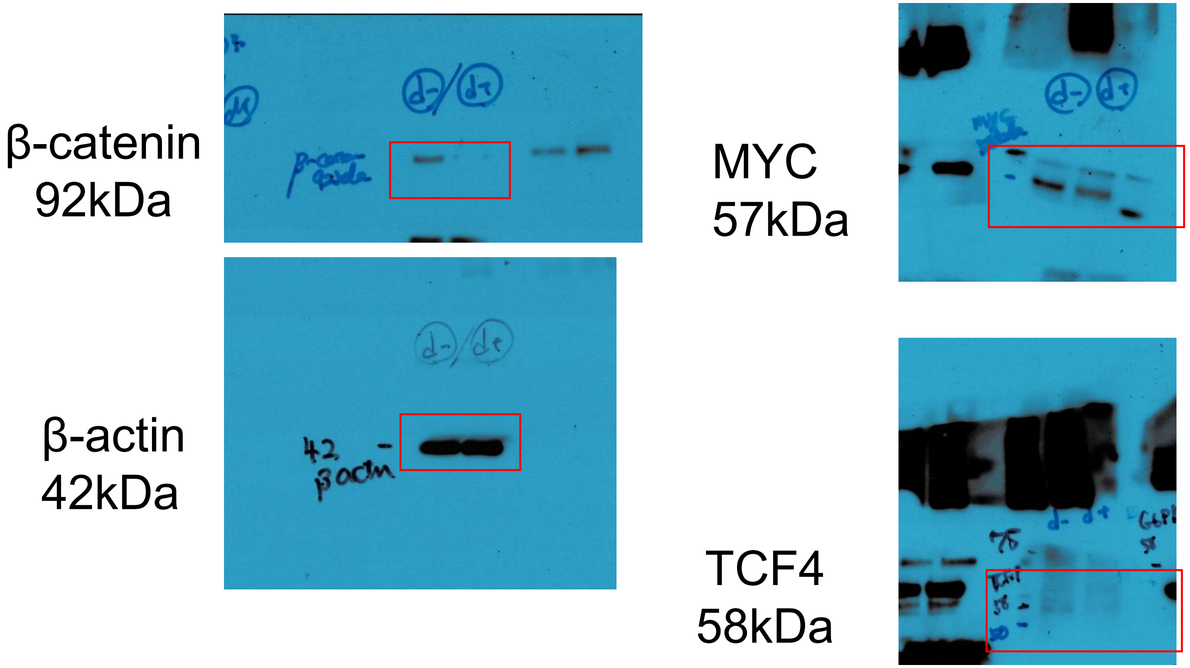

Figure 4C

MUC1-C
chromatin

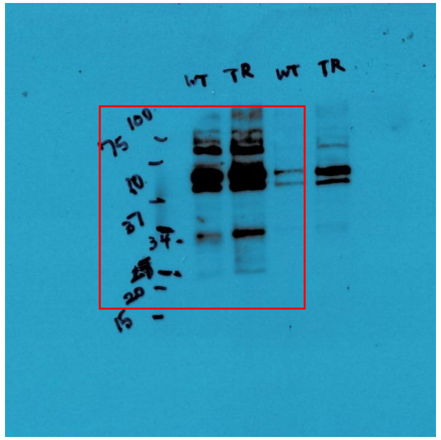

Histone H3
17kDa

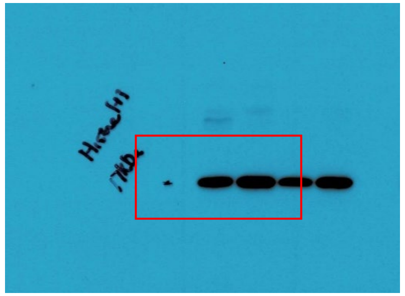

β-catenin
92kDa

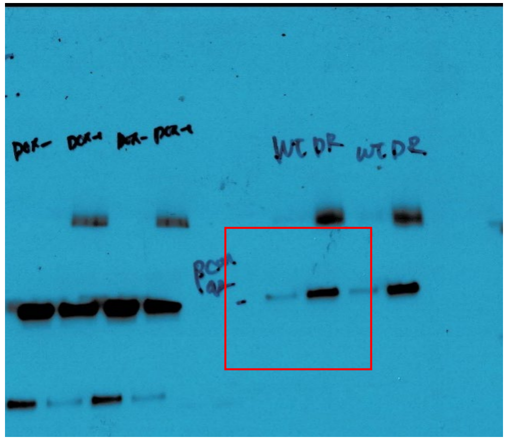

MYC
57kDa

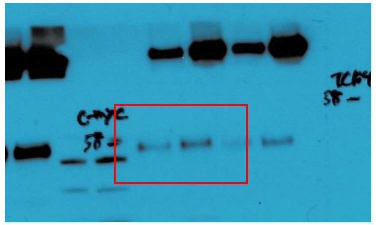

Histone H3
17kDa

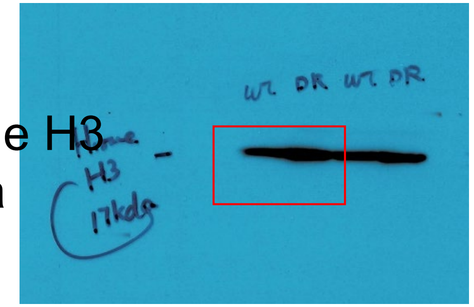

Figure 4D

MUC1-C
chromatin

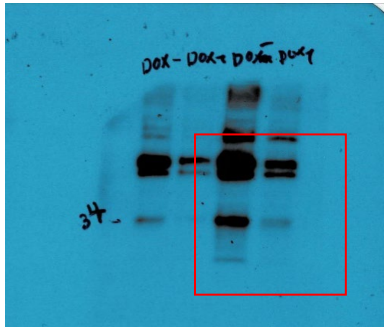

Histone H3
17kDa

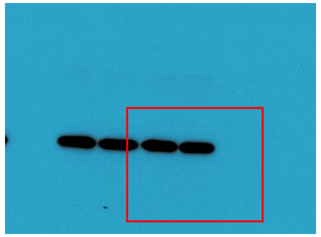

β-catenin
92kDa

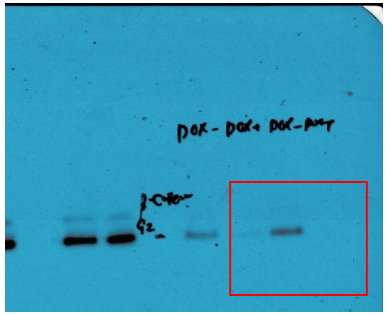

MYC
57kDa

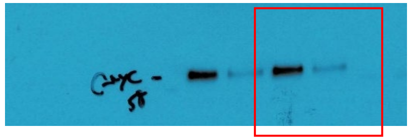

TCF4
58kDa

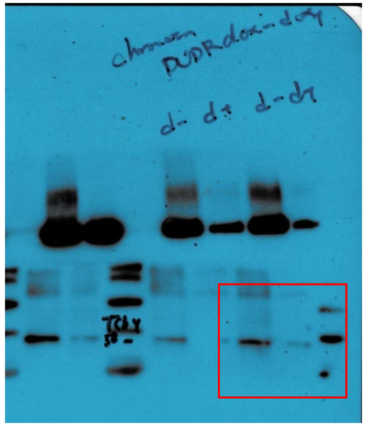

Histone H3
17kDa

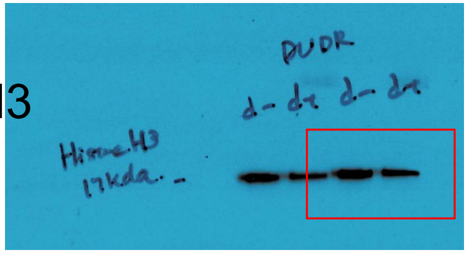

Figure 4F

LDHA
37kDa

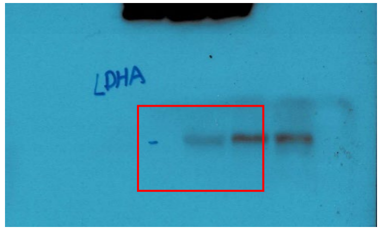

β-actin
42kDa

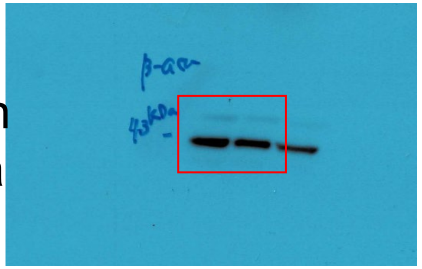

GLUT1
55kDa

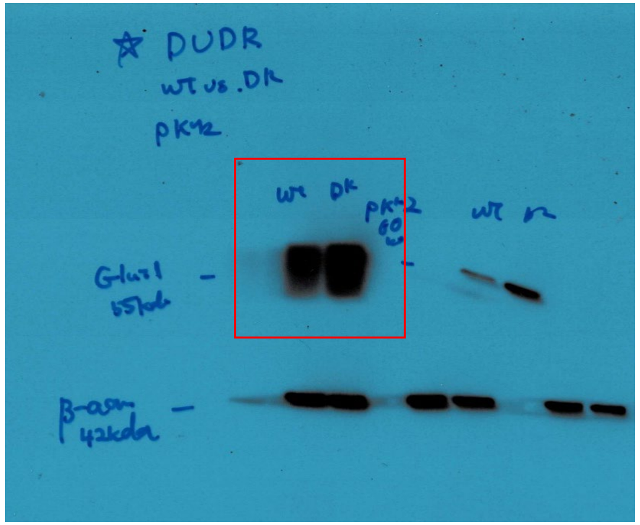

PKM2
60kDa

β-actin
42kDa

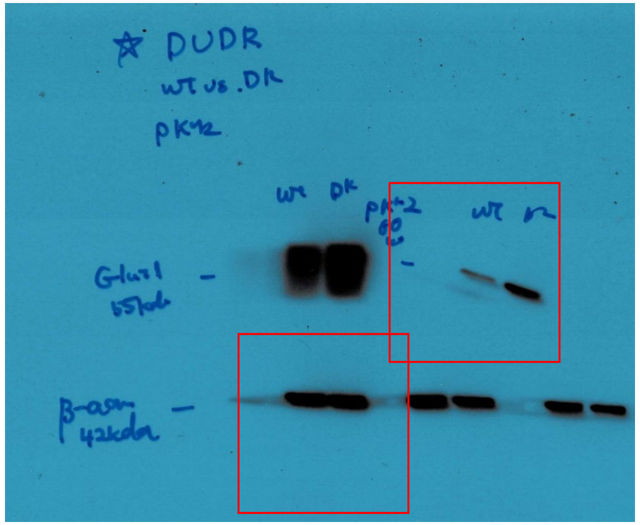

HK2
102kDa

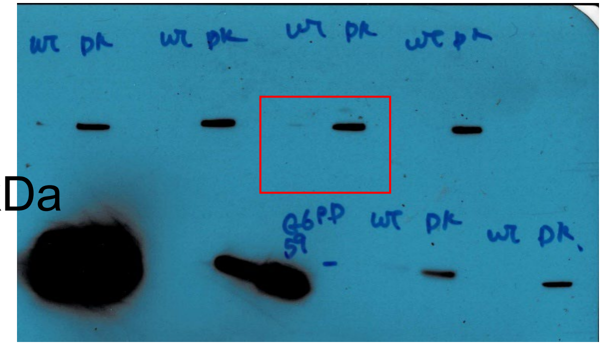

G6PD
59kDa

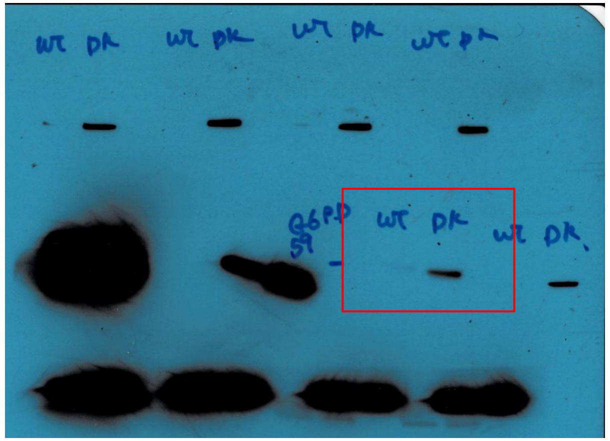

Figure 4G

G6PD
59kDa

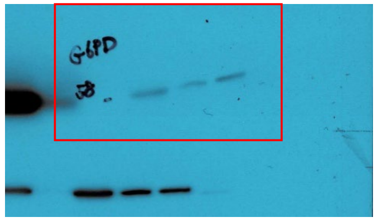

PKM2
60kDa

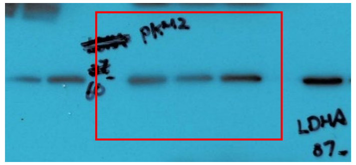

β-actin
42kDa

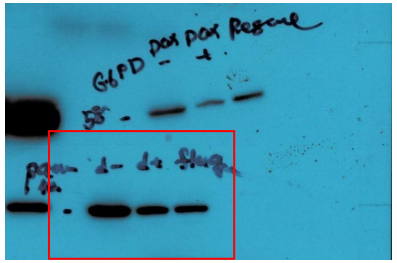

LDHA
37kDa

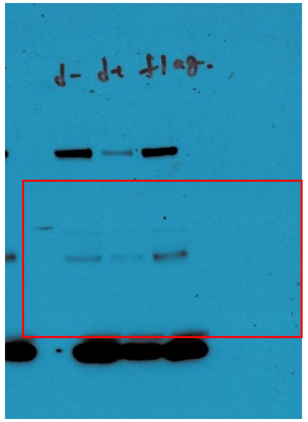

β-actin
42kDa

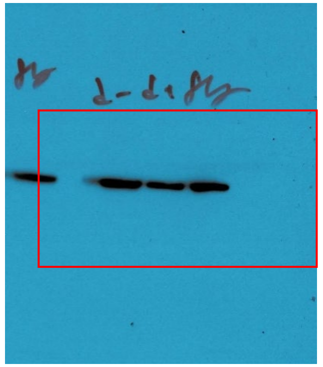

Figure 4I

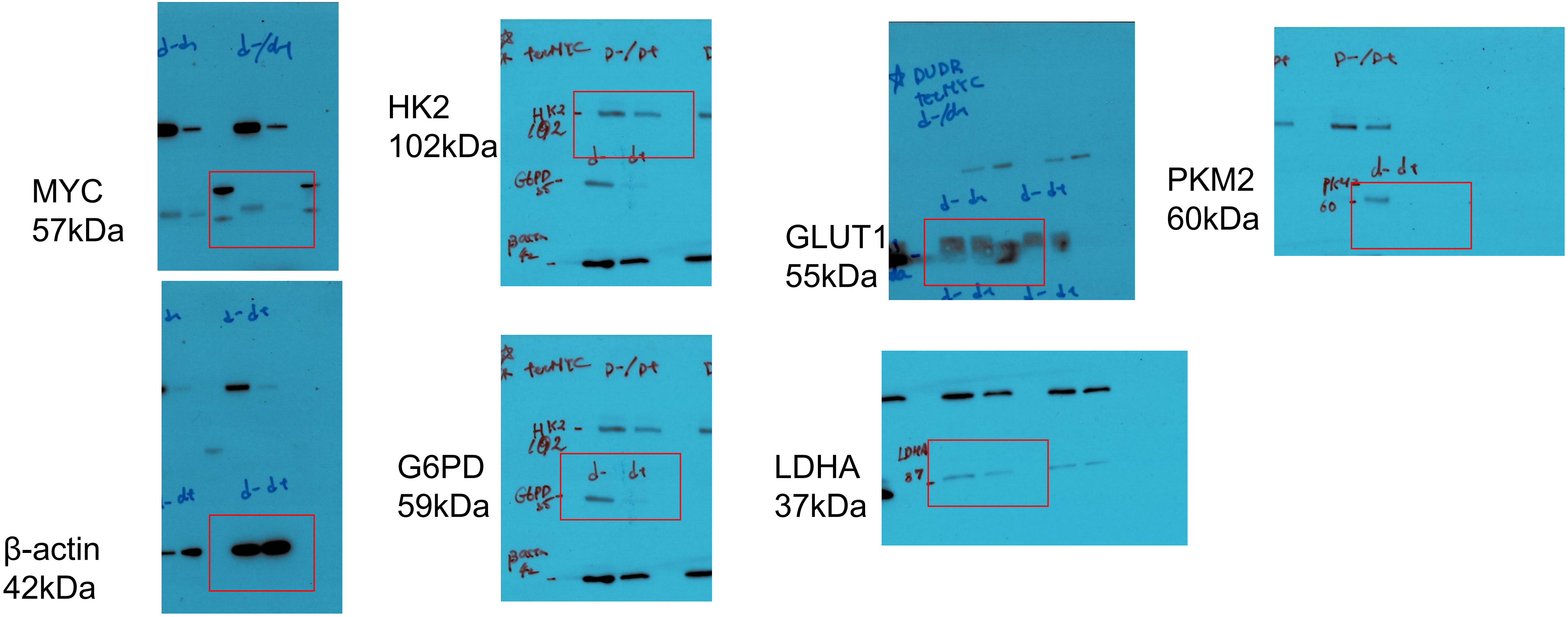
MYC
57kDa
HK2
102kDa
GLUT1
55kDa
PKM2
60kDa
β-actin
42kDa
G6PD
59kDa
LDHA
37kDa

Figure S4C

β-catenin
92kDa

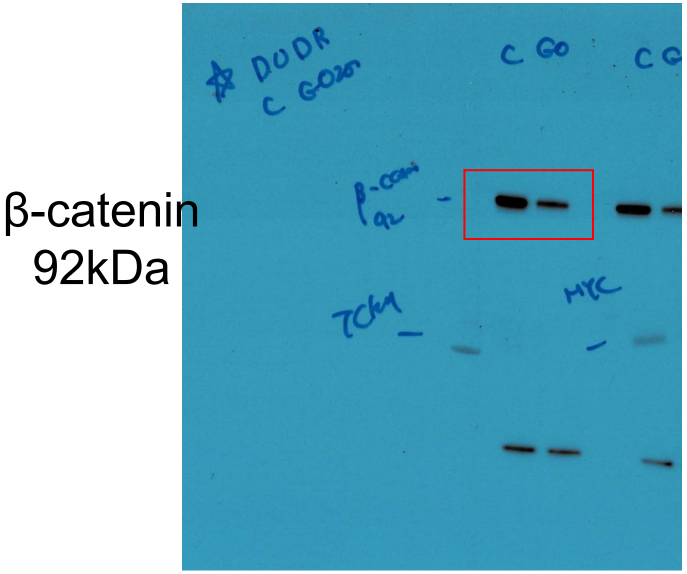

β-actin
42kDa

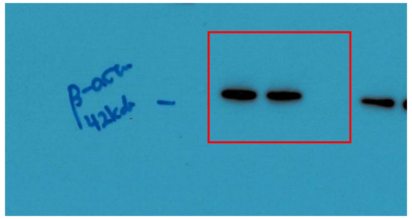

MYC
57kDa

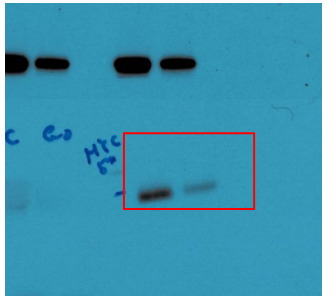

TCF4
58kDa

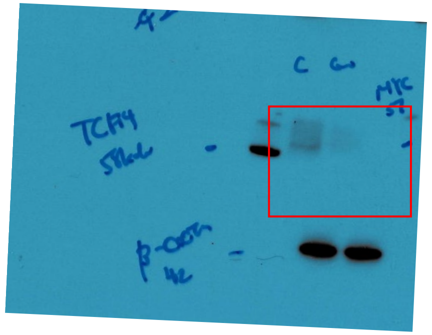

Figure S4D

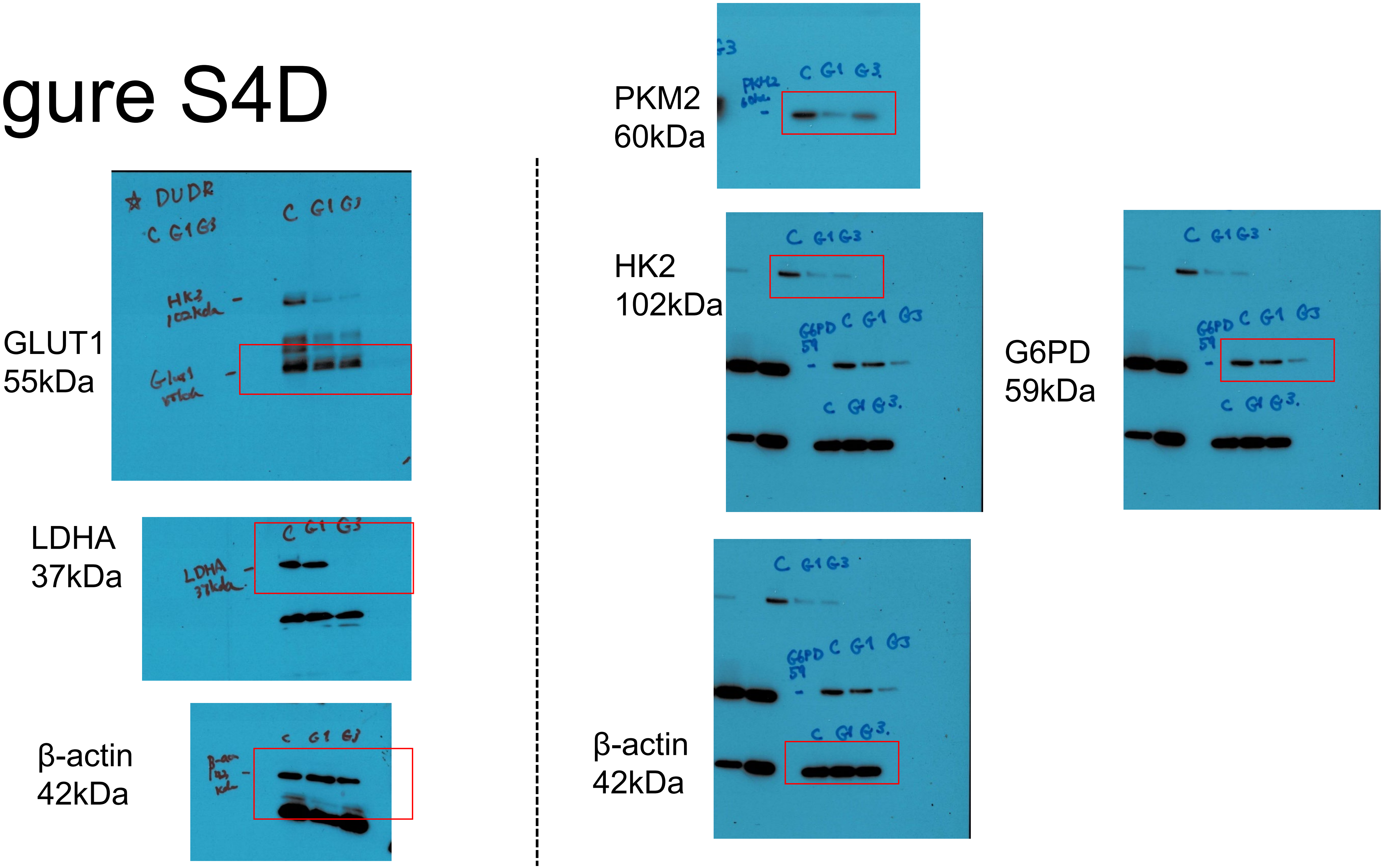
PKM2
60kDa
GLUT1
55kDa
HK2
102kDa
G6PD
59kDa
LDHA
37kDa
β-actin
42kDa
β-actin
42kDa

Figure 5E and 5F

MUC1-C
chromatin

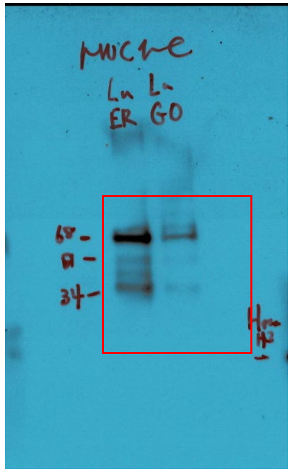

MYC
57kDa

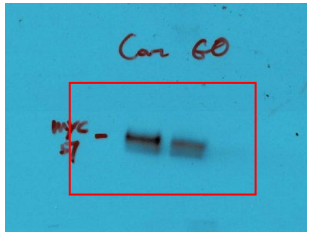

Histone H3
17kDa

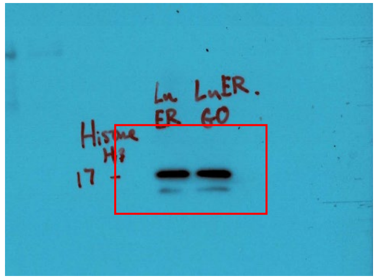

MUC1-C
chromatin

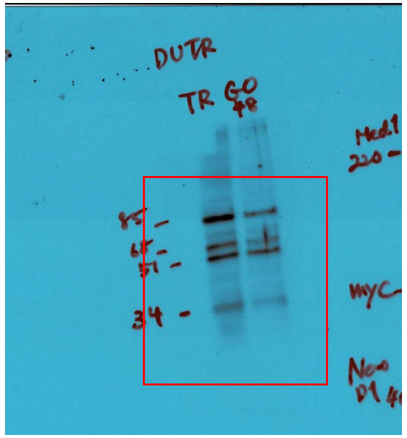

MYC
57kDa

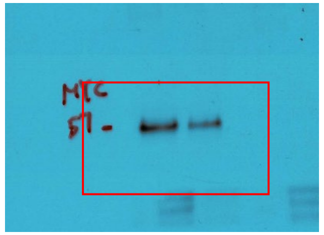

Histone H3
17kDa

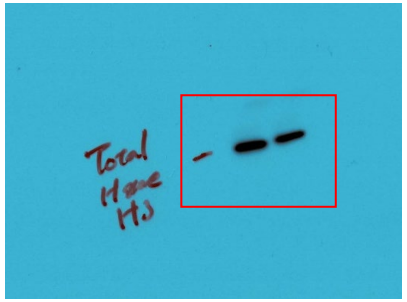

Figure 6B

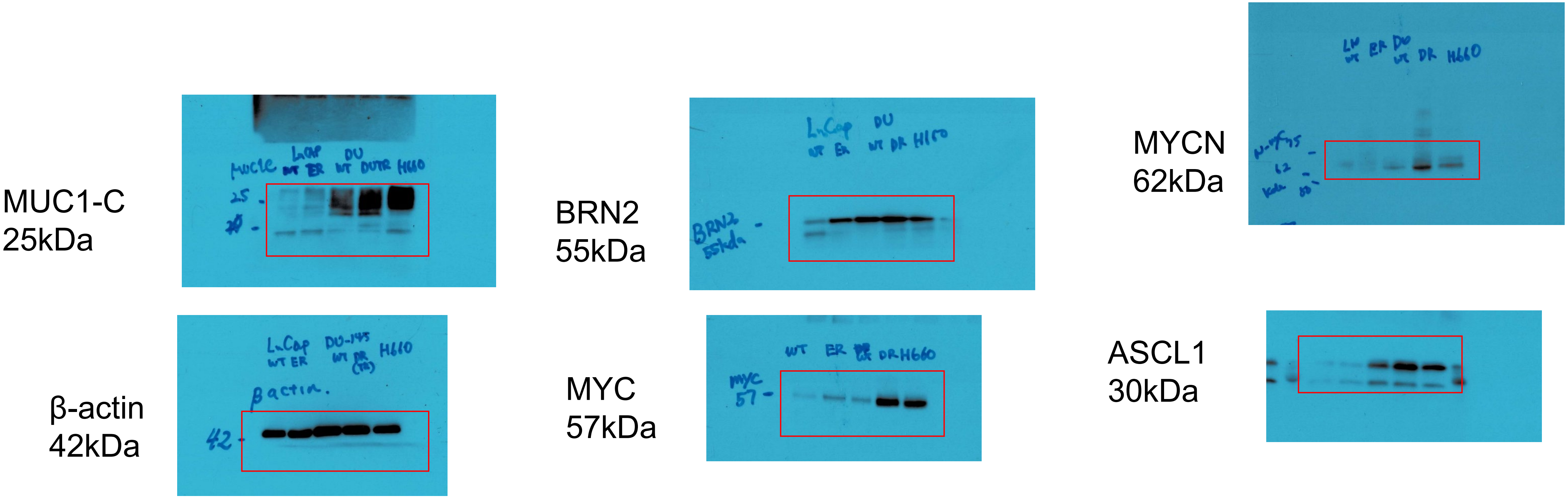
MUC1-C
25kDa
BRN2
55kDa
MYCN
62kDa
β-actin
42kDa
MYC
57kDa
ASCL1
30kDa

Figure 6C

MYC
57kDa

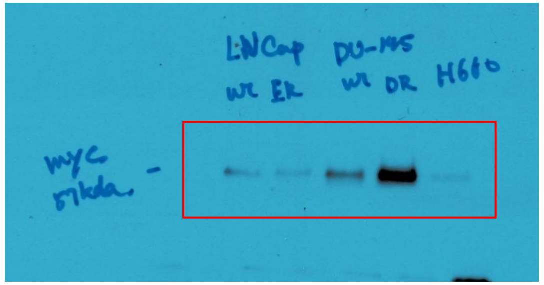

MYCN
62kDa

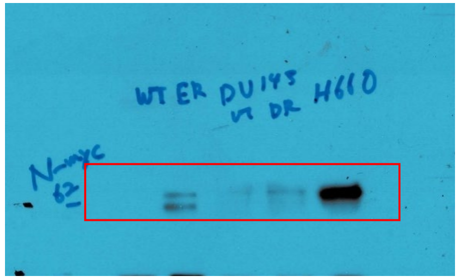

BRN2
55kDa

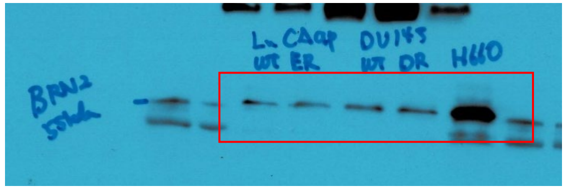

ASCL1
30kDa

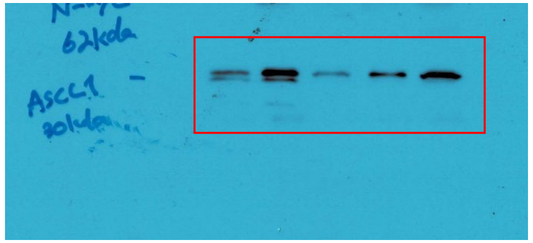

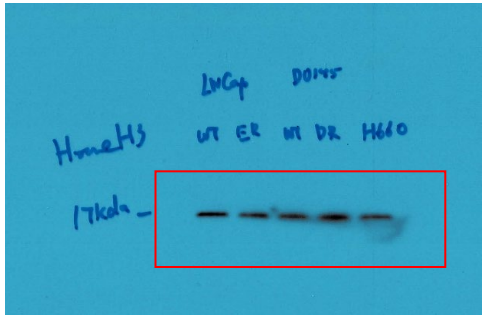

MUC1-C
Chromatin

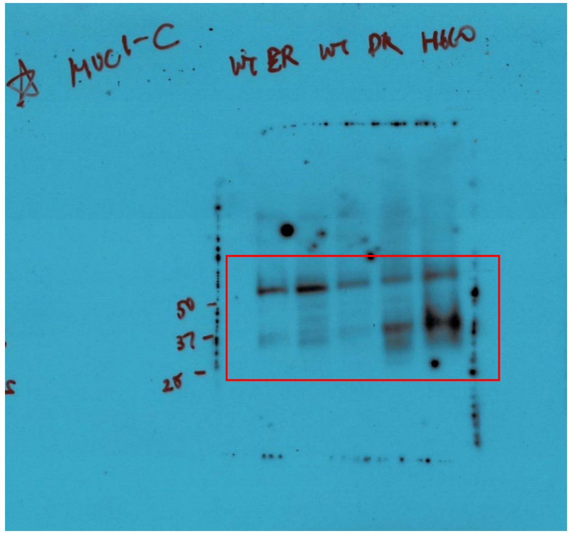

Histone H3
17kDa

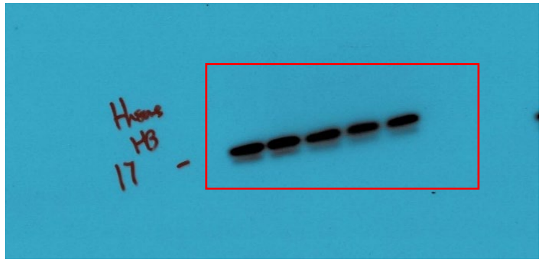

Figure 6E

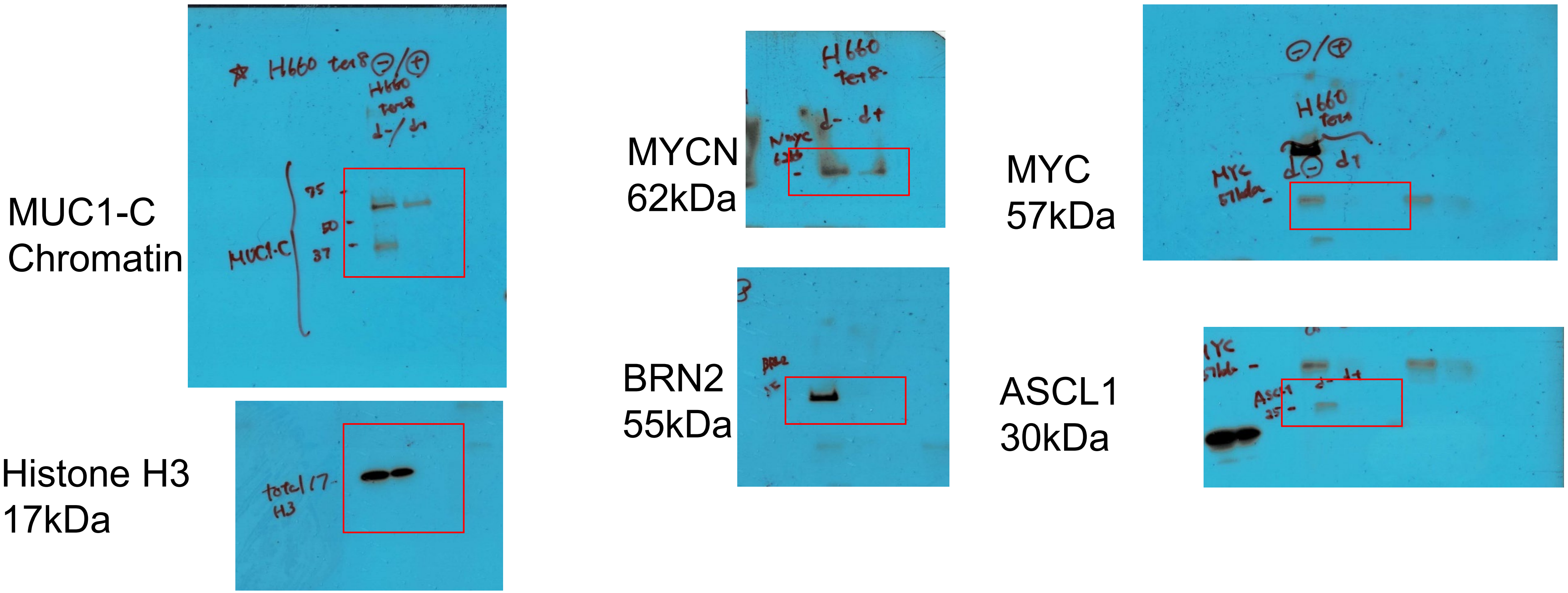
MUC1-C
Chromatin
Histone H3
17kDa
MYCN
62kDa
BRN2
55kDa
MYC
57kDa
ASCL1
30kDa

Figure 6G

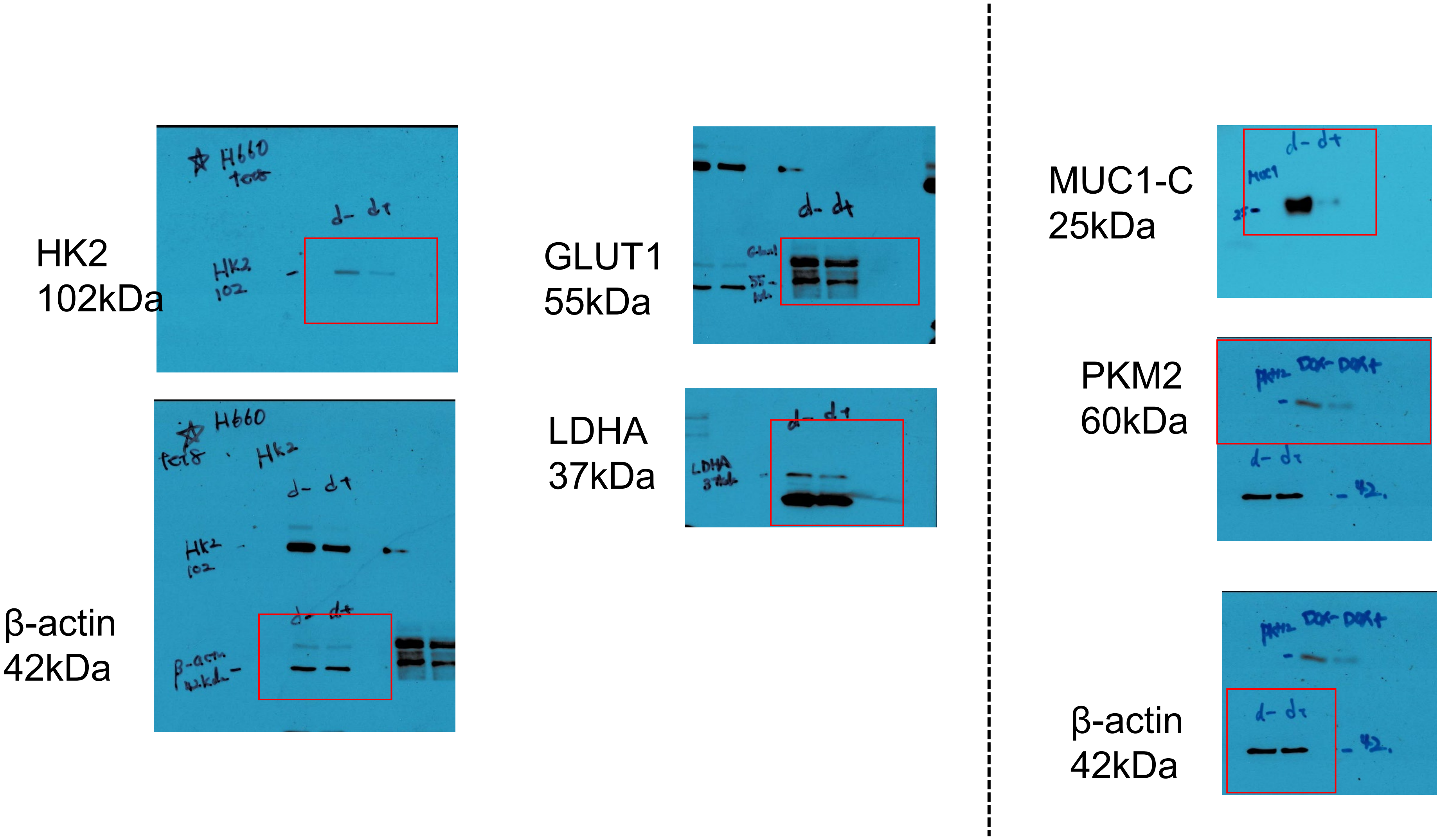
HK2
102kDa
β-actin
42kDa
GLUT1
55kDa
LDHA
37kDa
MUC1-C
25kDa
PKM2
60kDa
β-actin
42kDa

Figure 6H

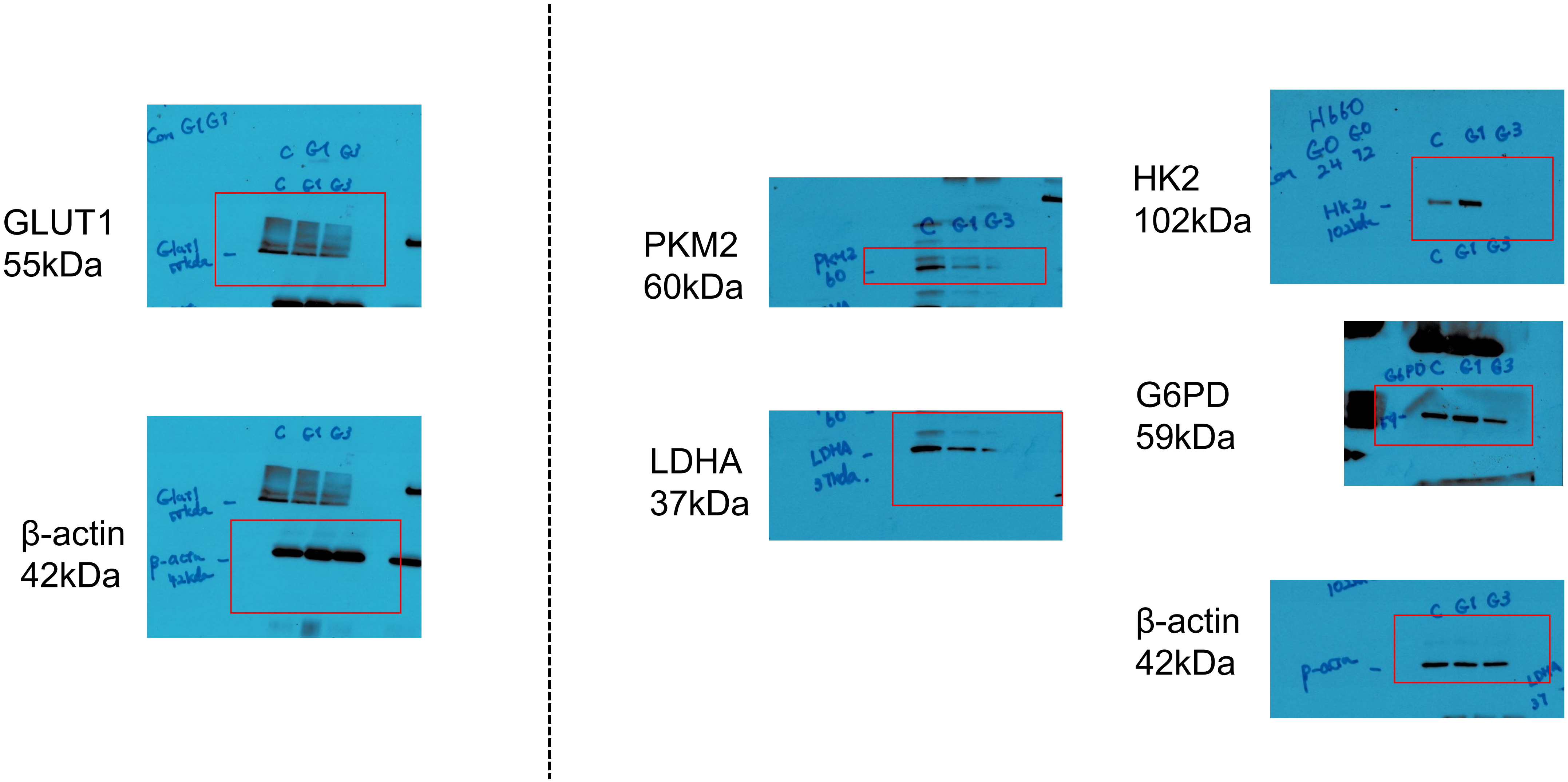
GLUT1
55kDa
β-actin
42kDa
PKM2
60kDa
LDHA
37kDa
HK2
102kDa
G6PD
59kDa
β-actin
42kDa

Figure 7B

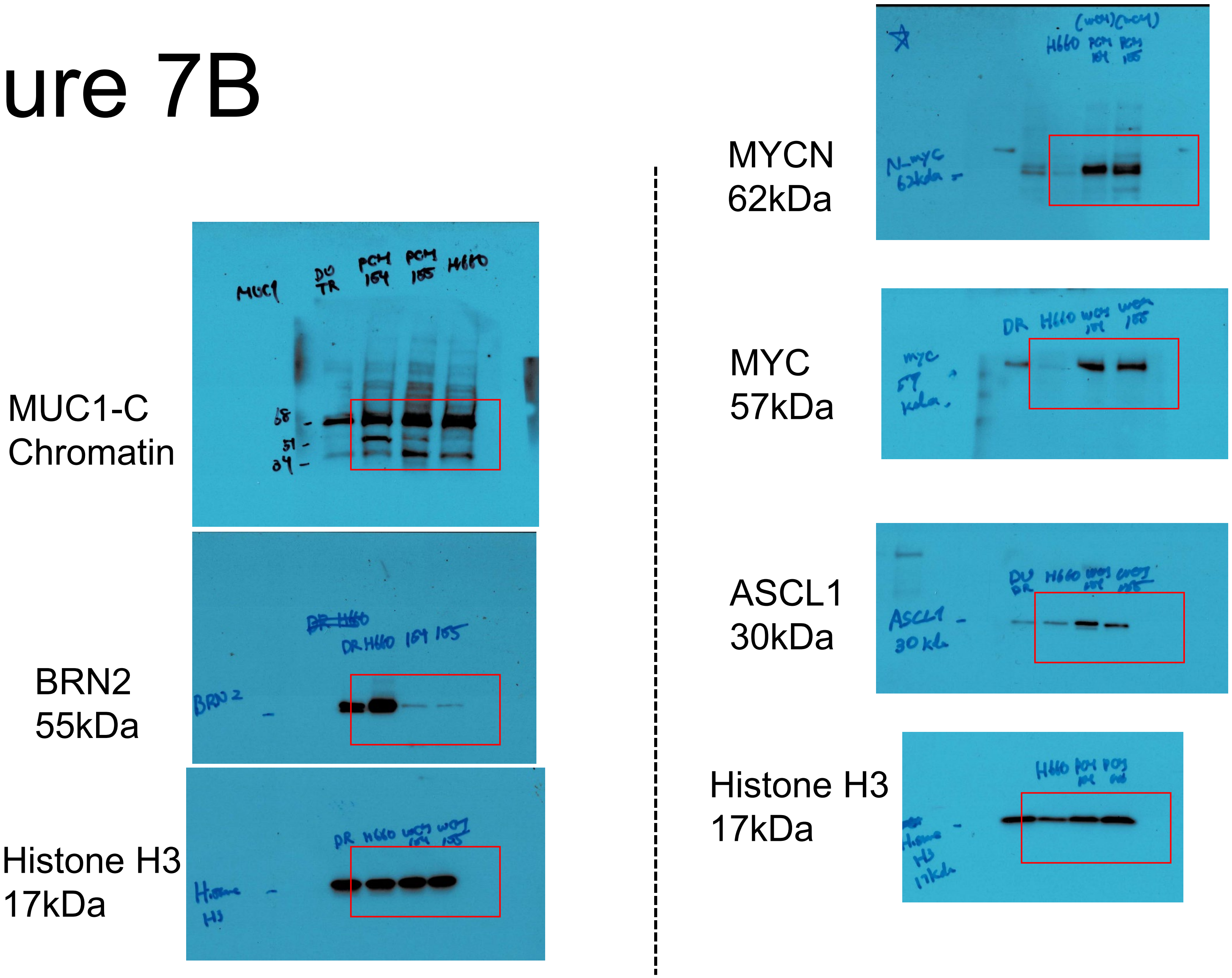

MUC1-C
Chromatin
BRN2
55kDa
Histone H3
17kDa
MYCN
62kDa
MYC
57kDa
ASCL1
30kDa
Histone H3
17kDa

Figure S6A

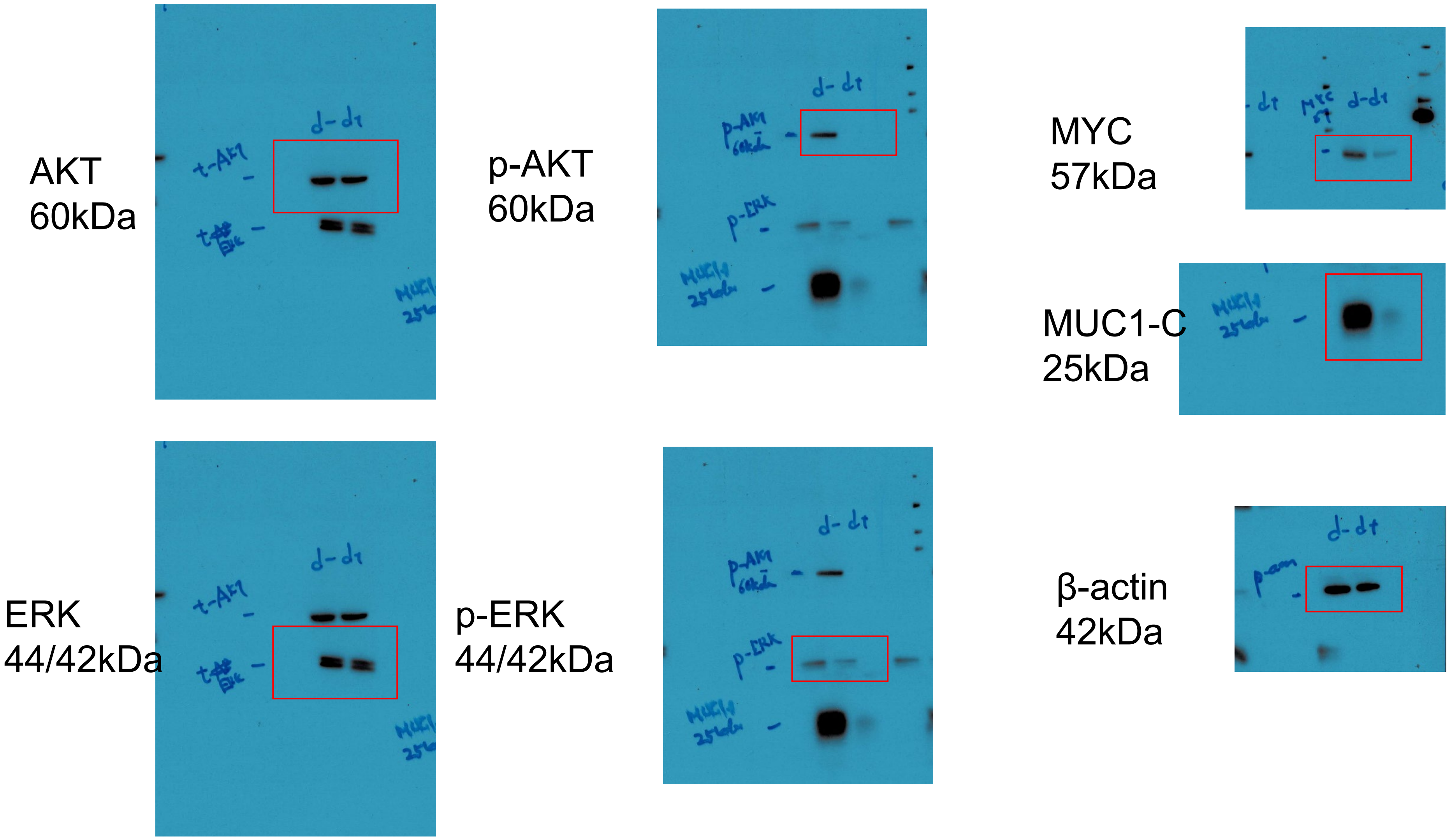
AKT
60kDa
p-AKT
60kDa
MYC
57kDa
MUC1-C
25kDa
ERK
44/42kDa
p-ERK
44/42kDa
β-actin
42kDa

Figure S6B

β-catenin
92kDa

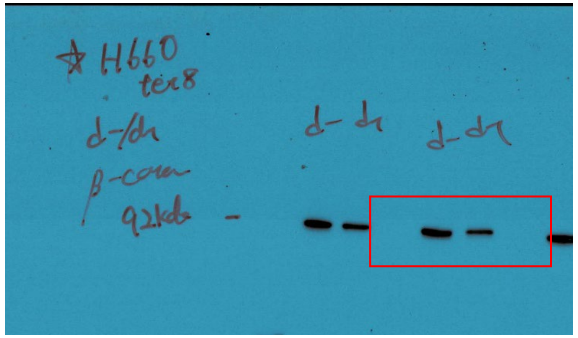

MYC
57kDa

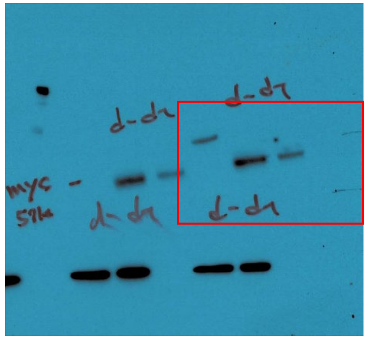

TCF4
58kDa

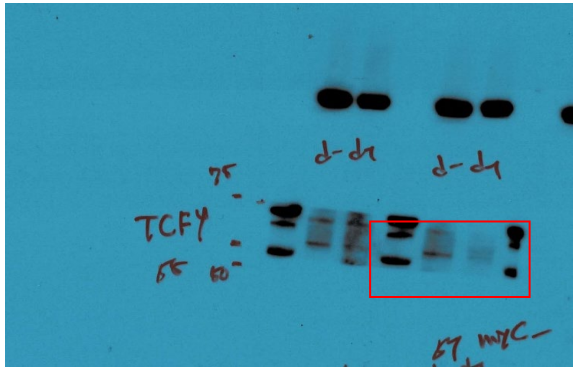

β-actin
42kDa

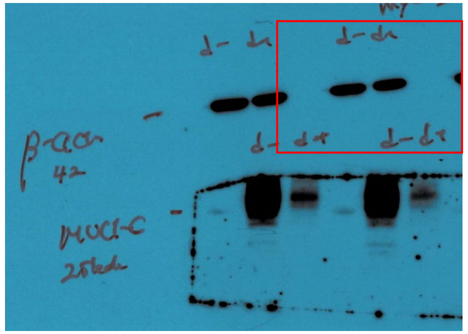

Supplement: Unedited blot and gel images [file jciinsight-10-190924-s081.pdf]
